# Spa visits carry measurable carbon footprints that vary with energy use and renewables

**DOI:** 10.1038/s44458-026-00122-x

**Published:** 2026-07-22

**Authors:** Robert Hanea, Finn McFall, Xavier Font, Jonathan Chenoweth, Ionut Corduneanu, Eduard Goean, Dabo Guan, Lorenzo Fioramonti, Jhuma Sadhukhan

**Affiliations:** 1 Therme Group RHTG AG, Vienna, Austria; 2 Centre for Environment and Sustainability, University of Surrey, Guildford, UK; 3 Surrey Hospitality and Tourism Management, University of Surrey, Guildford, UK; 4 Surrey Business School, University of Surrey, Guildford, UK; 5 Department of Earth System Sciences, Tsinghua University, Beijing, China; 6 The Bartlett School of Construction and Project Management, University College London, London, UK; 7 NATIVA, Rome, Italy

**Keywords:** Environmental impact, Technology, Sustainability

## Abstract

Spa and wellness centres constitute a fundamental aspect of contemporary health and lifestyle infrastructure, yet their environmental impacts remain under-assessed. This study presents a dynamic emissions calculator tailored to spa and wellness facilities, designed to generate transparent per-visitor emissions and support decarbonisation and benchmarking. By combining metered data such as energy and water with normalised non-metered sources including waste and staff travel, the model quantifies per-visitor emissions across Scopes 1, 2, and 3 and provides time-resolved profiles showing daily and seasonal patterns. Validation uses data from a large wellness resort and a small university sports park wellness zone. The results show that a typical visit results in average emissions of ~5 kilograms of carbon dioxide equivalent at the large facility and ~3.5 kilograms at the small facility, with on-site renewable energy in 2024 reducing emissions by ~33% and ~14% respectively.

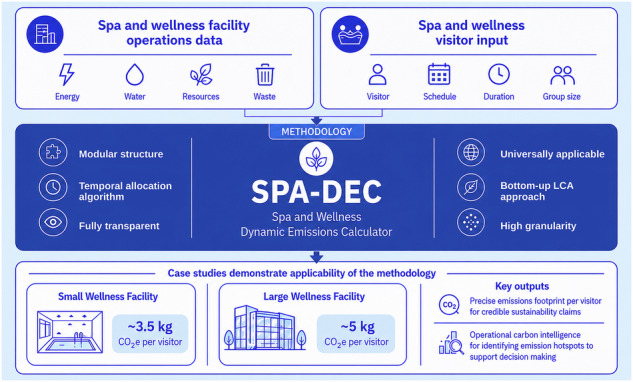

## Abstract (web summary)

SPA-DEC is a dynamic carbon calculator that translates a facility’s metered energy and water use, together with waste and other operational sources, into the footprint of a single spa or wellness visit. Across two case-study facilities in 2024 it found typical visits of about 3.5 to 5 kilograms carbon dioxide equivalent, with on-site renewables reducing per-visit emissions by roughly 14 to 33%

## Introduction

The spa and wellness industry is growing fast, its associated energy costs are substantial^1^ and most energy-reduction efforts are the result of cost-cutting activities^2,3^. Until recently, spas and wellness facilities rarely had sustainability strategies: from none in medical tourism properties surveyed in Hungary in 2017^2^, to half in the health tourism facilities in Poland in 2022^3^. There is a scarcity of dedicated studies on energy consumption and carbon accounting specifically within spas and wellness centres^4,5^. The spa and wellness industry currently lacks a standardised per-visit emission metric: without consistent boundaries and allocation methods, facility-level footprints cannot be compared or benchmarked, limiting the sector’s ability to measure or communicate progress on decarbonisation. This highlights a significant gap: the spa and wellness industry currently lacks both the data and tools needed to quantify and manage its climate impact in a rigorous way.

Spa, thermal and wellness leisure and tourism are different offerings within the broader category of the health visitor economy. However, the terms are often used interchangeably, because the business models of suppliers and motivations of users are blurred, with much innovation happening at the intersection of these^1,6^. Spas typically use thermal and mineral waters, often together with other services. Experts identify a range of sustainability issues that influence the design and delivery of spa and wellness services, ranging from energy and water demands to product sourcing and the positioning of spas as vehicles to re-connect to nature^6^. Cost-efficiency in purchasing and operations has historically been the priority; a reduction of greenhouse gas emissions may be a byproduct of such actions, but not the driver^6^. Only recently architects and operators have begun to place stronger emphasis on natural light, and a more efficient use of water and energy in facility design and operations^6,7^.

In parallel, many spa businesses have branded themselves as “eco-spas”, and there are multiple eco-labels and certification schemes specific for the spa industry that evaluate on as many as 300 specified criteria (for example, Green Globe Spa benchmarking and Green Spa Network guidelines)^8^. However, the extent to which these schemes provide quantified, comparable carbon metrics – such as emissions relative to facility size or emissions per visitor – remains unclear. Current methodologies rarely align with widely used international guidance and standards such as GHG Protocol, ISO 14064, and Life Cycle Assessment (LCA) under ISO 14040. This is particularly important given fast-changing requirements for Corporate Sustainability Reporting (CSR)^9,10^ and the strengthening of rules around environmental claims; for example, the EU Empowering Consumers for the Green Transition Directive, which restricts vague or generic environmental claims and increases expectations for clear justifications^10–12^.

Empirical evidence on spa-specific footprints remains limited and uneven in scope. Atalay and Demir ^13^ calculated the carbon footprint of 10 spa facilities in Turkey and 10 in Lithuania based on the direct electricity, gas and water consumption^13,14^. Using the reported facility sizes and daily user numbers, these results imply footprints of ~8.6 kg CO_2_e for the Turkish spas, and 8.0 kg CO_2_e for the Lithuanian spas. However, the study did not adopt a full LCA approach, as the study focused specifically on direct energy consumption, and they did not report methodological details such as emission factors and allocation choices in depth, which limits comparability and interpretability across facilities^13,14^. Earlier work has also considered spa-related impacts in adjacent contexts: Castellani and Sala^15^ used LCA to evaluate a hotel stay that included thermal spa facilities, estimating that electricity dominates the climate change impact, but their published results did not provide a clear carbon footprint for the overall stay or its thermal-spa component^15^. Because large spa and wellness centres frequently include aquatic facilities, relevant methodological insights also emerge from indoor swimming pool literature, where operational energy consumption often dominates life cycle impacts^16^. Rana et al.^16^ applied a process-based LCA framework to an aquatic centre comprising three pools and a hot tub with a maximum capacity of 961 people. Energy demands were estimated using component-level models for pool heating, pool pumps, the heating ventilation and air conditioning systems, lighting, and filtration systems. Across seven operating strategies, climate change impacts ranged from 39 to 49 kt CO_2_e over a 50-year service life, and hotspot analysis showed energy use contributed 87–94% of total environmental impacts^16^. Complementary studies using detailed building-services modelling (EnergyPlus, TRNSYS, OPENFOAM) show both the potential for sizable efficiency gains and the practical difficulty of modelling complex air and water equipment interactions without extensive calibration, with persistent uncertainty even in well-specified models^17–24^.

A further challenge is methodological consistency. Although a Product Environmental Footprint (PEF) Category Rule for spa services does not yet exist, the PEF methodology is already aligned with ISO 14040/44 and is expected to expand into tourism-related categories, demonstrated by the development of a PEF Category Rule for hotel accommodation services^25^. Carbon accounting encompasses the recognition, evaluation and monitoring of GHG emissions across different scales – national, organisational, project, and product levels^26^. ISO 14064 provides guidelines for organisational GHG emissions and verification processes^27^, while the GHG Protocol categorises emissions into three scopes: Scope 1 GHG emissions are direct emissions from owned or controlled sources, Scope 2 GHG emissions are indirect emissions from purchased electricity, heating, and cooling, and Scope 3 GHG emissions are all other indirect emissions within a company’s value chain^28^. LCA standards (ISO 14040/44) provide a framework for assessing impacts across a product or service life cycle^29^ and emissions are commonly expressed as CO_2_ equivalents (CO_2_e) based on Intergovernmental Panel on Climate Change (IPCC) global warming potential values^30^. In practice, reporting remains constrained by incomplete data, particularly for Scope 3 emissions^31^, and inconsistencies in boundary setting and allocation can reduce comparability across studies and facilities^32–34^. Emerging best practice also emphasises that Scope 3 emissions often represent the largest portion of a footprint^35^, reinforcing the need for approaches that can integrate both metered operational flows and less directly observed sources.

Per-visitor and per-guest-night carbon metrics are more established in adjacent visitor-economy sectors than in spas. In hotel accommodation, the EU Product Environmental Footprint Category Rule for hotel accommodation services^25^ and hotel-specific carbon tools such as the Hotel Carbon Measurement Initiative^36^ apportion whole-property emissions to a guest-night using floor area and occupancy-based allocation, but rely on annual input data rather than temporally resolved emission factors. Tourism LCA studies^15^ and aquatic-centre LCAs^16^ provide rigorous life cycle inventories but are facility or scenario-level rather than visit-level and are too data-intensive for routine operational use. For spas specifically, the only published footprints divide annual direct energy by user numbers^13^, without time resolution, without closed-hour treatment, and without documented emission-factor provenance. No existing method therefore combines visit-level temporal allocation, an explicit open and closed-hour distinction, and standardised, traceable LCA-aligned emission factors applied at hourly rather than annual resolution, with on-site renewable infrastructure represented through life cycle emission factors rather than assumed to be zero, yielding an auditable, reproducible per-visit footprint. This is the gap addressed here.

The dominance of indirect emissions, inconsistent boundary and allocation choices, and uneven data availability in practice mean that a spa-specific tool must combine scope-based reporting with LCA-aligned emission-factor selection, make allocation rules explicit and reproducible, and remain functional across data maturity levels. Within this context, three broad methodological approaches to per-visitor carbon accounting can be identified, each with distinct trade-offs. The simplest is a static annual ratio, which divides total annual facility emissions by total annual visitors to produce a single average per-visit value. While straightforward to compute, this approach ignores temporal variation entirely. It cannot distinguish between a visit during a quiet weekday morning and a peak weekend afternoon, nor can it capture the influence of seasonal renewable energy availability on emissions intensity. A more sophisticated approach applies dynamic hourly allocation without distinguishing between open and closed operational periods. Although this captures intra-day variation, it introduces a systematic distortion: during low-occupancy hours, a disproportionately large share of fixed operational emissions is allocated to each visitor, inflating the per-visit footprint in ways that do not reflect the marginal cost of an additional visit. The open and closed-hour distinction resolves this, separating emissions incurred during visitor-facing operations from the facility’s maintenance baseline – the overnight and preparatory load consumed regardless of visitor presence – so that closed-hour emissions are shared proportionally across all visitors rather than concentrated on those present during quiet periods.

This study adds to a suite of dynamic emission calculators^37^ by introducing SPA-DEC, a Dynamic Emissions Calculator for Spa and Wellness Centres, which offers a sector-specific method to calculate the carbon footprint of a visitor stay using a clearly defined per-visitor functional unit (kg CO_2_e per visitor per defined duration). SPA-DEC combines real-time metered data (for example, energy and water consumption) with annually aggregated emissions from non-metered activities (for example, waste, consumables, and staff travel). The calculator then allocates whole-facility emissions to individual visitor stays through time-based attribution and normalisation, producing an output broken down by emission scope and, where data allows, by spa feature. The method is modular and scalable, supporting facilities of different sizes and data sophistication while aligning with international standards (GHG Protocol, ISO 14064 and ISO 14040/44). By translating whole-facility data into per-visitor footprints and enabling time-based attribution to individual visitor stays, SPA-DEC supports more meaningful benchmarking, CSR reporting and operational sustainability efforts. It also improves transparency and comparability across facilities with varying data availability, empowering both businesses and consumers to make informed sustainability decisions. Real-time, per-visitor footprints also support immediate carbon offsetting, offering a practical and actionable use case. In doing so, SPA-DEC not only engages visitors directly but also positions itself as a future-ready tool aligned with emerging carbon offsetting or removal mechanisms, including the blockchain-based carbon tokenisation model proposed by Goean et al.^38^, which requires precisely the kind of verified, visit-level attribution that SPA-DEC’s dynamic allocation produces.

In this study, the results of SPA-DEC case studies on a large wellness resort and a small university sports park wellness zone are presented. Using high-resolution operational data, the emissions footprint per visitor is calculated and broken down by source. The output is analysed over time of day and season. The results show not only emissions per visit, but also how SPA-DEC can be used to assess how renewable energy integration can significantly reduce emission impacts. The general methodology is then explained for complete transparency.

## Results and discussion

### Impact assessment

#### Core model and total facility emissions

The main output of this study is a generic, universal SPA-DEC framework that can be applied across diverse facilities of certain characteristics, which merit their distinct presence in the governing (high-level) equations as follows, to calculate the time-resolved greenhouse gas (GHG) emissions through the SPA-DEC framework, detailed in the Methodology section.

At its core, the SPA-DEC model follows a fundamental emissions calculation formula:

1 $${{\rm{E}}}=A\times {EF}$$

where *A* is activity data, such as energy consumption, and ${EF}$ is the emissions factor in kg CO_2_e per unit of activity data.

The total carbon footprint of a visitor’s spa visit is calculated as:

2 $${{{{\rm{CO}}}}}_{2}{{{\rm{e per visitor}}}}={\sum}_{h\in {H}_{{visit}}}\frac{{E}_{h}}{{N}_{h}}+\frac{{E}_{d}^{{closed}}}{{N}_{d}}\,+{\sum}_{{{{\boldsymbol{V}}}}}\frac{{x}_{V}}{{N}_{A}}+{\sum}_{{{{\boldsymbol{B}}}}}\frac{{x}_{B}}{{N}_{A}}$$

Where ${E}_{h}$ is the total metered emissions from all sources $s$ during hour $h$, obtained using:

3 $${{{\rm{E}}}}_{h}={\sum}_{s}{A}_{s,h}\times {{EF}}_{s}$$

${H}_{{visit}}$ is the set of open hours that overlap with the visitor’s stay in the resort $\left[{t}_{0},{t}_{1}\right]$ on day $d$, and ${N}_{h}$ is the number of visitors present during hour $h$. The first term attributes open-hour emissions to the visitor in proportion to the occupancy of each hour.

${E}_{d}^{{closed}}$ are the total emissions accrued during closed hours on day $d$, allocated equally across ${N}_{d}$, the unique visitors recorded on that day.

${x}_{V},\,{x}_{B}$ represent non-metered visitor and business operation emissions for each category $V,B$. These emissions are measured annually and divided by the total number of annual visitors, ${N}_{A}$, to give the per-visitor impact.

Individual terms in Eq. (2) are comprehensively addressed incorporating full life cycle stages or embedded emissions for each foreground inventory and the nature of the time-series foreground inventory data is also discussed in the Methodology section.

The SPA-DEC framework was applied to two contrasting real-world facilities for the calendar year 2024: a university swimming-pool and wellness zone based in the UK (Small Wellness Facility) and a large wellness resort based in Romania (Large Wellness Facility).

The total annual global warming potential or GHG emission impacts (in CO_2_e) (referred as emission in the paper) for the Large Wellness Facility were ~7437 t CO_2_e across the reporting year, of which 4447 t CO_2_e arose from metered energy and water consumption and 2990 t CO_2_e from non-metered categories including fugitive refrigerant losses, inbound logistics, staff commuting, and spend-based procurement estimates. The Small Wellness Facility reported a total emission of 242 t CO_2_e, of which 225 t CO_2_e derived from metered electricity and natural-gas consumption and only 17 t CO_2_e from non-metered sources. The approximately thirty-fold difference in absolute emissions reflects the difference in facility scale: the Large Wellness Facility served 1,620,619 visitors in 2024, whereas the Small Wellness Facility hosted 74,973 visitors. As the size and capacity can vary widely, a functional unit per visitor is well justified to calculate via this developed robust dynamic emission calculator.

#### Scope breakdown

Table 1 summarises how the activity data used in the two case studies map onto Scopes 1–3 and how each parameter contributes to the emission totals reported in this section. Metered operational flows (purchased electricity, on-site gas, on-site geothermal, on-site photovoltaic and water) provide time-resolved activity data and therefore drive the hourly and seasonal variability explored in the subsequent figures. In contrast, several Scope 3 emissions are typically available only as annual measures derived from procurement records, contractor reporting or financial accounts (for example, waste, consumables, inbound logistics and additional goods and services).

**Table 1:** Mapping of SPA-DEC activity-data inputs to GHG Protocol scopes.

Scope	Emission source	Primary data source	Input parameter(s)
Scope 1	On-site natural gas combustion	Metered utility	Hourly gas consumption (kWh)
	Fugitive emissions (refrigerants)	Maintenance logs/estimates	Refrigerant type and annual leakage/top-up (kg)
	Company transport	Fleet logs/mileage	Fuel type and distance
Scope 2	Purchased electricity	Metered utility	Hourly purchased electricity consumption (kWh)
Scope 3	On-site photovoltaic	Metered utility	Hourly photovoltaic production/consumption (kWh)
	On-site geothermal	Tracked utility	Hourly geothermal (kWh)
	Water supply and wastewater treatment	Metered utility/contractor invoices	Hourly water consumption (m^3^) and proportion of wastewater
	Waste	Contractor invoices	Waste mass by type (kg)
	Consumables	Procurement	Mass purchased (kg)
	Additional goods and services	Financial accounts	Spend by cost centre/category (€)
	Inbound logistics	Supplier data/estimates	Mode of transport, distance and mass
	Staff commuting	Survey/estimates	Mode split and distances
	Business travel	Expense claims/travel bookings	Mode and distance
	Company accommodation	Expense claims/travel bookings	Location/duration/type

Each emission source within the framework is assigned to its GHG Protocol scope – Scope 1 (direct emissions), Scope 2 (purchased energy) and Scope 3 (value-chain emissions) – alongside its primary data source and the input parameter(s) required. Metered utilities provide time-resolved hourly activity data, whereas the remaining Scope 3 categories are typically available only as annual totals from procurement, contractor or financial records. On-site photovoltaic and geothermal generation appear under Scope 3 because their point-of-generation emissions are zero, with only the embodied life-cycle emissions of the infrastructure counted. The categories listed are those available to the framework; not all apply to every facility.

**Fig. 1:**
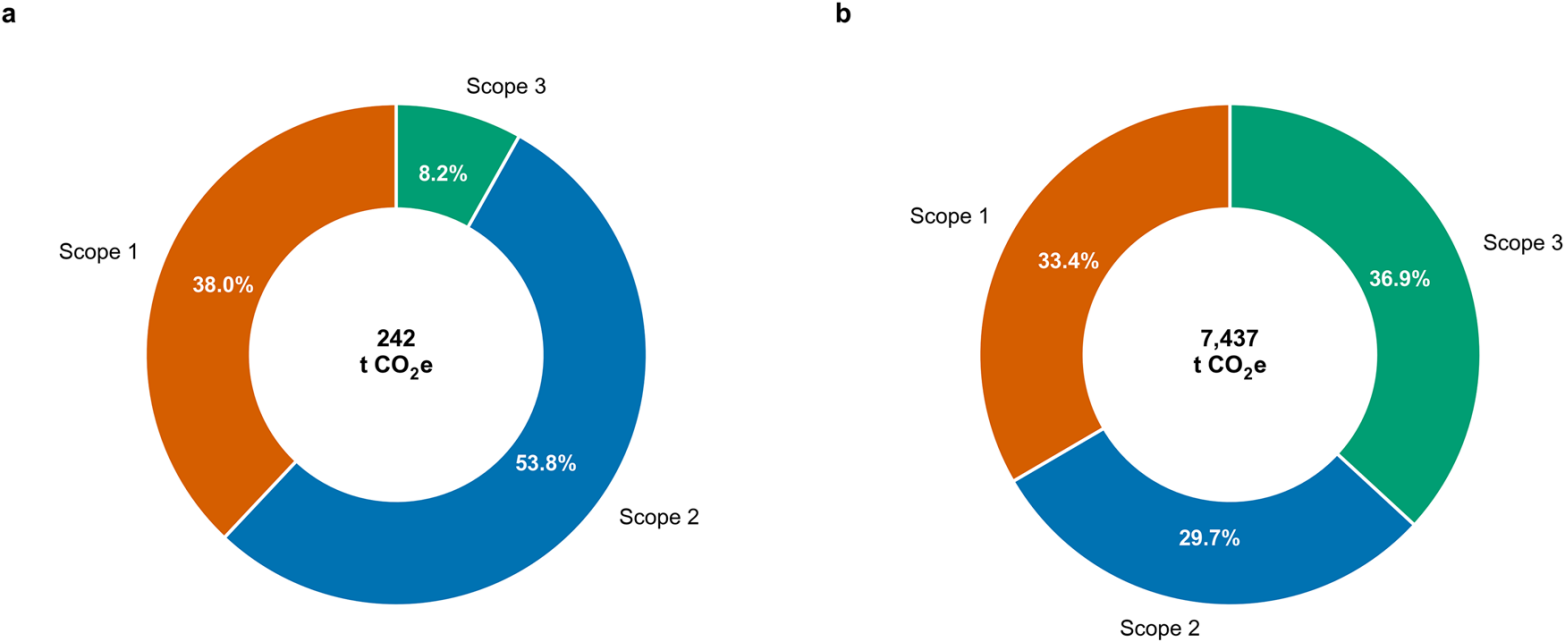
GHG protocol scope composition of annual facility emissions. Annual greenhouse-gas emissions for 2024 apportioned to Scopes 1, 2 and 3 as a percentage of each facility's total; central labels give the absolute annual total. **a** Small Wellness Facility (242 t CO_2_e). **b** Large Wellness Facility (7,437 t CO_2_e). Scope 1 (orange) covers direct combustion, fugitive refrigerants and company transport; Scope 2 (blue) is purchased electricity; Scope 3 (green) covers value-chain categories including water, waste, procurement, logistics and staff travel, together with the embodied emissions of on-site photovoltaic and geothermal infrastructure.

Figure 1 presents the GHG Protocol scope classification for each facility. The two sites exhibit markedly different scope profiles, reflecting their divergent energy mixes and operational structures.

At the Small Wellness Facility, Scope 1 accounts for 38.0% of the total emissions, driven predominantly by on-site natural-gas combustion for pool-zone heating, with smaller contributions from company transport and fugitive R410A losses from the pool air-handling unit. Scope 2 contributes 53.8%, while Scope 3 categories collectively represent 8.2%. The resulting profile shows Scope 1 and Scope 2 together accounting for over 90% of emissions.

The Large Wellness Facility presents a more balanced distribution: Scope 1 accounts for 33.4%, reflecting the material contributions of on-site natural-gas combustion, fugitive refrigerant losses, and company transport. Scope 2 contributes 29.7%, while Scope 3 categories account for 36.9%, including the life cycle embodied emissions from the geothermal heat and photovoltaic infrastructure. On-site photovoltaic and geothermal energy incurs no point of generation emissions. The embodied, upstream manufacturing and installation emissions of this infrastructure are accounted for separately as Scope 3, using life cycle emission factors from Ecoinvent. Reporting these two components separately keeps the inventory consistent with the GHG Protocol scope definitions while retaining the life cycle completeness of an LCA perspective; the two are complementary rather than alternative treatments of the same emissions^39^. The electricity used to operate the geothermal heat-pump system at the Large Wellness Facility is metered within the facility’s purchased electricity and accounted under Scope 2 via the time-resolved grid factor, distinct from the geothermal life cycle emission factor; operational and embodied emissions are therefore not double-counted. The relatively even split underscores the importance of full scope accounting for diversified thermal wellness facilities where supply-chain, life cycle infrastructure, and operational emissions are all material.

#### Metered consumption emissions

**Fig. 2:**
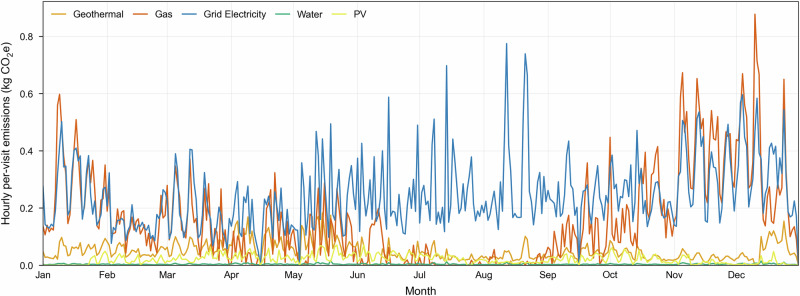
Hourly per-visit metered emissions at the Large Wellness Facility across 2024. Per-visit emissions (kg CO_2_e) from metered operational sources, resolved at hourly intervals and disaggregated by source: geothermal (orange), gas (rust), grid electricity (blue), water (green) and photovoltaic (yellow). Grid electricity and gas dominate and drive the pronounced winter peaks and day-to-day variability, while geothermal, water and photovoltaic contributions remain comparatively low and stable; photovoltaic and geothermal values represent embodied upstream emissions of the energy infrastructure rather than emissions at the point of generation.

Figure 2 presents the hourly per-visitor metered consumption emissions at the Large Wellness Facility during 2024, disaggregated by electricity, gas, geothermal, photovoltaic (PV), and water. The plot illustrates the pronounced day-to-day and seasonal variability in emissions, with the largest fluctuations driven by grid electricity and gas. Emission intensities rise sharply in the winter period and exhibit intermittent spikes, consistent with periods when demand is higher and a larger share of energy is met by grid and gas back-up rather than low-carbon supply.

The seasonal variation in grid electricity emissions is driven not only by changes in facility demand but by the composition of the national electricity grid itself, which varies materially across the year. Using hourly grid generation data sourced from the national transmission system operator (Supplementary Fig. S2 shows the monthly grid consumption, split by source), the purchased electricity mix was resolved at hourly resolution for each month of 2024. The data show that hydroelectric generation dominates supply during spring and summer months, suppressing the grid emission factor during the period of lowest facility demand. Conversely, coal and gas-fired generation represent a greater share of supply during winter months, coinciding with peak facility heating and electrical loads. Purchased electricity is characterised with the highest resolution grid factor available. By linking hourly grid composition data to hourly facility electricity consumption, SPA-DEC can apply a temporally resolved emission factor rather than a flat annual average. This is central to the framework’s advantage over static approaches. A single annual grid emission factor could systematically understate emissions intensity in winter and overstate it in summer, obscuring the true seasonal amplitude of per-visitor footprints.

By contrast, geothermal and water-related emissions remain relatively stable year-round, reflecting more consistent baseload provision and less sensitivity to short-term operating conditions. PV contributions appear as a small but increasingly visible contribution following commissioning in early 2024 and coincide with periods of reduced reliance on grid electricity. Emissions arising from PV and geothermal are embodied upstream manufacturing emissions of the energy infrastructure, rather than direct GHG releases. Figure 2 illustrates the central advantage of SPA-DEC for operational analysis. By combining high-frequency data handling with per-visitor normalisation, the method captures variability at the temporal scales that matter for both facility management and visitor-level attribution.

**Fig. 3:**
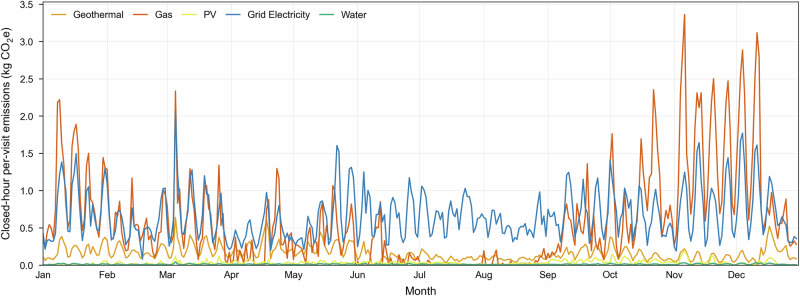
Closed-hour overhead emissions allocated per visit at the Large Wellness Facility across 2024. Per-visit emissions (kg CO_2_e) accrued during closed hours, disaggregated by metered source: geothermal (orange), gas (rust), photovoltaic (yellow), grid electricity (blue) and water (green). Closed-hour emissions represent the facility's maintenance baseline – overnight thermal storage, recirculation and preparatory loads – and are allocated equally across the unique visitors recorded on each calendar day and are therefore expressed per visitor rather than per visitor-hour. Gas and grid electricity dominate, with the highest overheads in winter; water and photovoltaic contributions remain small.

Figure 3 shows the closed-hour per-visitor emissions at the Large Wellness Facility for 2024, disaggregated by metered source. In contrast to the open-hour profile, these values represent overhead emissions accrued when the facility is not receiving visitors and are therefore expressed on a per-visitor basis rather than a per visitor-hour basis. As a result, the magnitude of the closed-hour signal is higher: the same pool of out-of-hours emissions is distributed across the visitors who attend, whereas open-hour emissions scale with the duration of each individual stay.

The temporal pattern remains dominated by gas and grid electricity, with markedly higher overhead intensities during winter months when baseline thermal and electrical loads are greatest. Geothermal and water-related contributions are comparatively stable, while PV remains small. This distinction between open-hour and closed-hour components is central to SPA-DEC’s visitor allocation logic: it separates shared facility overheads from time-dependent operational loads, ensuring that visitor footprints reflect both the timing of a visit and the facility’s baseline operating requirements.

### Renewable energy scenario modelling

**Fig. 4:**
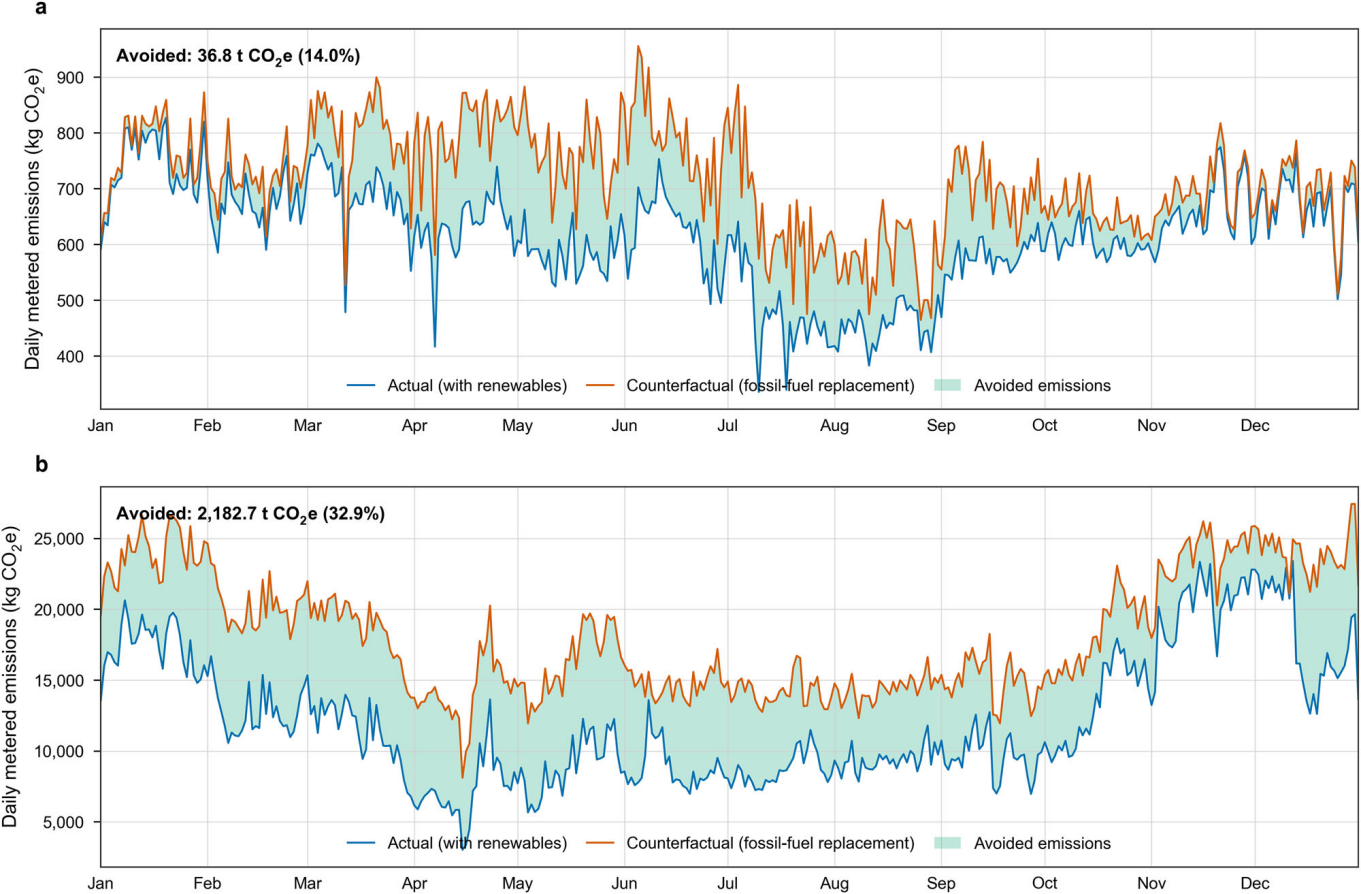
Avoided metered emissions from on-site renewable supply, observed versus counterfactual. Daily metered source emissions (kg CO_2_e) across 2024 under the observed 2024 energy configuration (actual, with renewables; blue) and a counterfactual in which on-site renewable supply is replaced by grid electricity and gas (orange); the shaded band marks the avoided emissions between the two. **a** Small Wellness Facility, where on-site photovoltaic generation avoids 36.8 t CO_2_e (14.0% of metered emissions). **b** Large Wellness Facility, where geothermal and photovoltaic supply avoid 2,182.7 t CO_2_e (32.9%). The avoided gap widens during periods of elevated energy demand.

Using SPA-DEC, a comparison can be made between each facility’s observed 2024 energy configuration, which integrates on-site geothermal supply and photovoltaic generation, with a counterfactual scenario in which thermal demand is met by on-site gas and electricity demand by the grid. Figure 4 shows that the counterfactual scenarios consistently yield higher daily emissions across the year, with the gap widening during periods of elevated energy demand.

For the Small Wellness Facility, the counterfactual replaces the 198 MWh of allocated on-site PV generation with grid electricity. Under this scenario, annual metered emissions would rise from 225 to 262 t CO_2_e, implying that the on-site PV array avoids ~36.8 t CO_2_e over 2024, a 14.0% reduction in metered source emissions. The timeseries reveals that the avoided-emission gap is widest during the summer months, when PV generation peaks and would otherwise displace the highest volume of grid electricity. During winter, reduced solar irradiance narrows the gap substantially, confirming that the decarbonisation benefit of on-site PV at the Small Wellness Facility is strongly seasonal.

The Large Wellness Facility’s 2024 energy-related emissions amounted to 4447 t CO_2_e, compared with 6629 t CO_2_e under the grid and gas scenario. This corresponds to an emission reduction of 2183 t of CO_2_e, ~32.9%, attributable to geothermal and PV supply. Beyond demonstrating mitigation potential of on-site renewables in facilities, this analysis illustrates how SPA-DEC can be used to evaluate operational decarbonisation options using the same metered consumption data framework that underpins the per-visitor results. The timeseries shows that the avoided-emission gap is persistent year-round owing to the continuous thermal load served by geothermal but widens markedly during the winter season (October–March) when the overall thermal consumption requirement is largest, and thus geothermal extraction volumes peak. This finding confirms that the geothermal and PV systems at the Large Wellness Facility are a substantial decarbonisation asset, with the geothermal component providing a baseload emission reduction that complements the seasonal PV contribution.

### Daily footprint overview

The standard visit durations used here – 4.0 h at the Large Wellness Facility and 1.5 h at the Small Wellness Facility – reflect the typical commercial entry period at each site. SPA-DEC is not constrained to these durations; the dynamic allocation framework produces a footprint for any visitor stay length, and the illustrative durations are used here solely to enable consistent cross-month and cross-facility comparison.

**Fig. 5:**
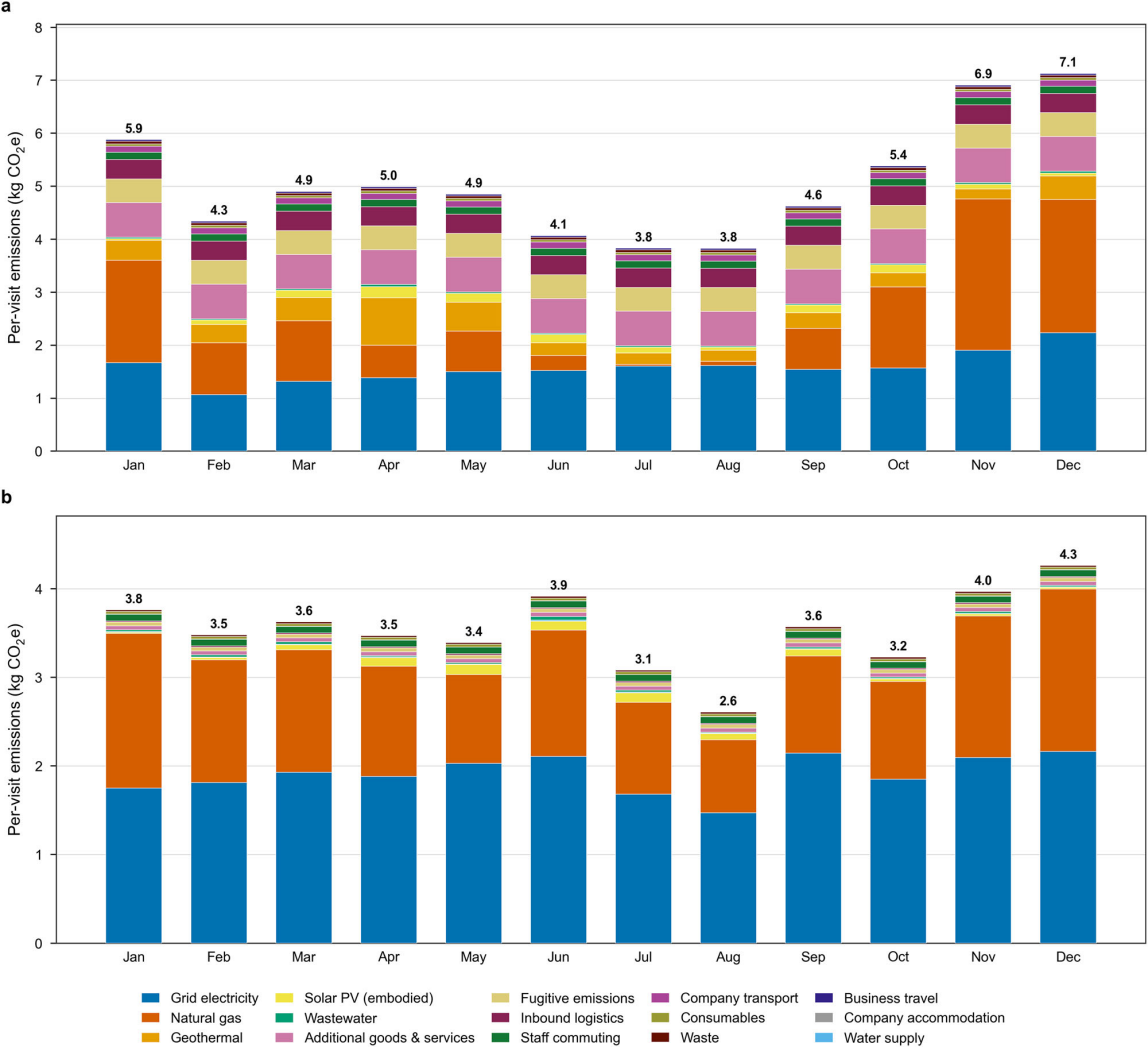
Monthly per-visit carbon footprint decomposed by emission source. Mean per-visit emissions (kg CO_2_e) for each calendar month of 2024, stacked by source, with the monthly total printed above each bar. **a** Large Wellness Facility for a standard 4.0-h visit. **b** Small Wellness Facility for a standard 1.5-h visit. Stack segments follow the order of the key; grid electricity and natural gas dominate in both facilities, with the remaining, mainly Scope 3, categories forming a thin and largely constant per-visit layer.

Figure 5 decomposes the per-visit footprint by individual emission source for each facility at its standard visit duration, presented as monthly averages to reveal how source-level contributions evolve across the year. The per-visit footprint is partially sensitive to visit duration. The open-hour metered component scales with stay length, while the closed-hour overhead and non-metered components are fixed per visitor regardless of duration. As a result, the total per-visit footprint scales sub-linearly with visit duration.

At the Large Wellness Facility, the monthly average per-visit footprint for a standard 4.0-h visit ranges from 3.83 kg CO_2_e in July and August to 7.13 kg CO_2_e in December, a seasonal amplitude of 3.30 kg CO_2_e. For context, Eurostat estimates the GHG footprint of goods and services consumed in the EU amounted to 29.31 kg CO_2_e per capita each day in 2022^40^. The largest contributors to the overall footprint are the facility’s grid electricity consumption used to back up the PV energy production, and on-site natural gas consumption used to supplement the geothermal thermal energy production. By contrast, other categories like water treatment, consumable goods, and waste management contribute comparatively small amounts per visitor. Monthly variation in the per-visit footprint reflects the interplay between seasonal energy demand and visitor throughput. October shows an elevated footprint relative to summer months, driven by declining PV contribution and lower visitor numbers, the latter increasing the overhead emissions allocated to each visit. December records the highest per-visit footprint, consistent with peak natural gas demand in winter months when geothermal backup requirements are greatest and PV generation is negligible (Supplementary Fig. S1).

At the Small Wellness Facility, a standard 1.5-h visit produces a monthly average emission ranging from 2.61 kg CO_2_e in August to 4.26 kg CO_2_e in December, a seasonal amplitude of 1.65 kg CO_2_e. The two metered energy sources, grid electricity and natural gas, dominate the per-visit footprint in every month, with non-metered categories contributing a thin, constant baseline layer.

Across both facilities, the monthly decomposition highlights two key features of the SPA-DEC model: first, the strong seasonal modulation of metered per-visit emissions, driven by the interplay of energy demand and occupancy; and second, the constant per-visitor non-metered contribution, which provides a full understanding of how non-metered, mainly Scope 3 emissions are allocated per visitor.

#### Temporal trends

**Fig. 6:**
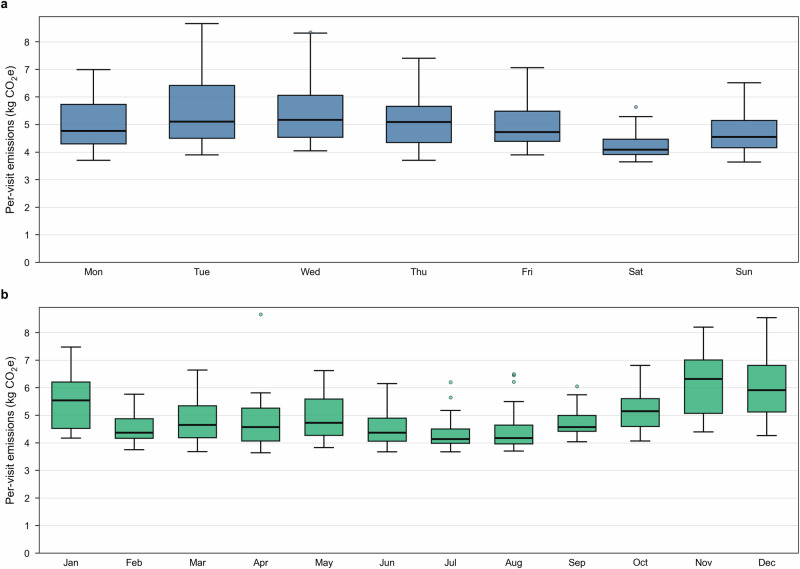
Distribution of per-visit emissions by day of week and by month at the Large Wellness Facility. Per-visit emissions (kg CO_2_e) for a standard 4.0-h visit in 2024, summarising the distribution of daily values within each group. **a** By day of week. **b** By calendar month. Boxes show the median (centre line) and interquartile range; whiskers extend to 1.5× the interquartile range and points beyond them are plotted individually. Per-visit emissions are lowest at weekends and in summer, when occupancy and the renewable share are highest, and highest on quieter weekdays and in winter.

SPA-DEC also reveals clear temporal patterns in visitor emission footprints. Figure 6 displays the distribution of per-visitor emissions for a four-hour visit at the Large Wellness Facility, segmented by weekday and month. Across the week, per-visit emissions are highest midweek and lowest on Saturdays, and their dispersion tracks their level: quieter weekdays show not only a higher median but a broader interquartile range and pronounced right skew, whereas Saturdays exhibit both the lowest median and the narrowest spread. Both patterns are consistent with an inverse relationship with visitor volume. When attendance is lower, a larger share of baseline and overhead emissions is allocated to each visitor, increasing the per-visit footprint. This dynamic is compounded by the facility’s energy demand profile. During opening hours, electricity and gas consumption rises sharply relative to the overnight baseline, reflecting the operational load of heating, filtration, lighting, and visitor-facing services. As visitor numbers fall on quieter weekdays, this elevated operational load is distributed across fewer visits, amplifying the per-visitor footprint effect.

The overnight period represents a structurally distinct emissions source. During closed hours, the facility maintains a continuous thermal baseline: excess heat is stored in boilers, and water is kept at temperature in preparation for the following day’s operations. This maintenance load is not attributable to any individual visit and is instead allocated proportionally across all visitors within the corresponding period. Bringing this closed-hour baseline into the emissions accounting is a methodological distinction of SPA-DEC. Rather than treating overnight consumption as negligible or excluding it from visitor-level calculations, the framework allocates it transparently, ensuring that the reported per-visit footprint reflects the full operational cost of delivering a visit, including the preparatory energy burden that precedes it.

Seasonality is also visible. Monthly medians decline through spring and reach their lowest levels in summer, before increasing again toward winter. This pattern aligns with the metered-consumption results, reflecting both weather-driven demand and the changing contribution of on-site renewables: during summer months, geothermal and PV systems meet a greater share of the facility’s energy needs and reduce reliance on gas and grid electricity, lowering the emissions intensity of a visit. These temporal trends indicate that the visitor footprint is shaped not only by what a facility consumes, but also by when a visit occurs and how fixed operational requirements are distributed across fluctuating attendance.

#### Sensitivity and uncertainty analysis

**Fig. 7:**
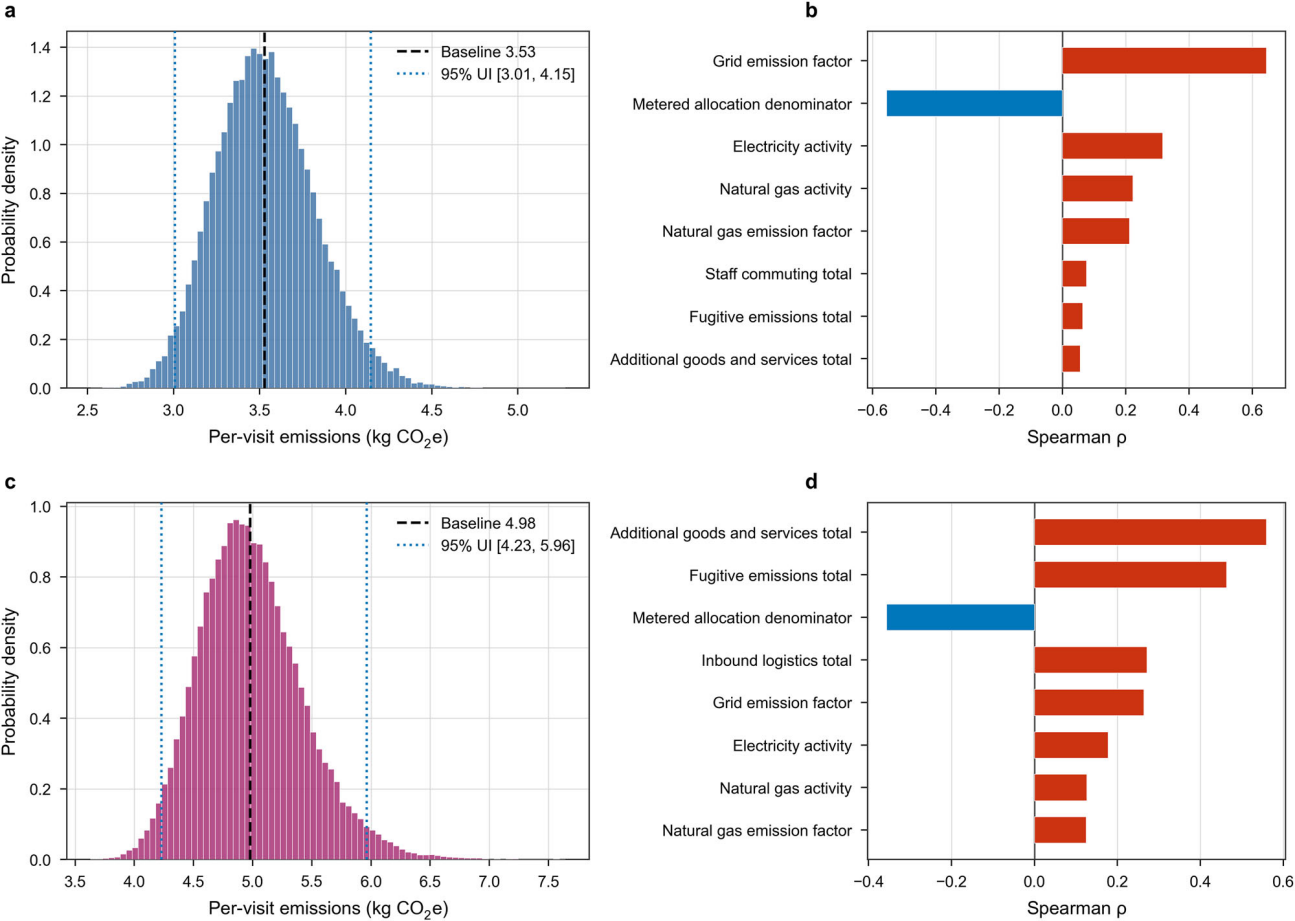
Monte Carlo uncertainty and global sensitivity of per-visit emissions. Parametric uncertainty propagated through the SPA-DEC allocation model using 50,000 draws per facility, with all uncertain inputs represented as unit-mean lognormal multipliers. Monte Carlo distributions of per-visit emissions (kg CO_2_e) for the Small Wellness Facility (**a**) and the Large Wellness Facility (**c**); the dashed line marks the deterministic baseline and the dotted lines the 95% uncertainty interval. Spearman rank correlation (ρ) between each input and the per-visit output for the Small (**b**) and Large (**d**) facilities, ordered by absolute correlation; positive correlations are red, negative correlations blue.

A 50,000-draw Monte Carlo analysis was performed independently for each facility to propagate parametric uncertainty through the SPA-DEC allocation model. All uncertain inputs were represented as standard unit-mean lognormal multipliers, with coefficients of variation (CVs) calibrated to reflect data quality: 5% for hourly metered activity data, 5–20% for secondary emission factors, 20–50% for annually estimated non-metered categories and fugitive-leakage terms. These coefficients of variation were assigned by structured expert judgement based on data provenance, following the principle that uncertainty scales inversely with the directness of measurement: directly logged hourly quantities (electricity, gas, water, and the occupancy denominator) are well constrained and receive low CVs; secondary emission factors drawn from literature or conversion tables are materially less precise; and annually estimated or spend-based categories are the least constrained and receive the highest CVs, reflecting their reliance on sparse records or input-output modelling. Per-input values and rationales are reported in Supplementary Table S1. The resulting per-visit distributions and global sensitivity rankings are presented in Fig. 7.

For the Small Wellness Facility, the Monte Carlo analysis yields a 95% uncertainty interval (UI) of 3.01–4.15 kg CO_2_e around the deterministic baseline of 3.53 kg CO_2_e per 1.5-h visit (Monte Carlo mean 3.54 kg CO_2_e, SD = 0.29 kg CO_2_e, CV = 8.2%). The coefficient of variation (CV), also known as relative standard deviation (RSD), is a statistical measure that quantifies the degree of variability in a dataset relative to its mean. It represents the standard deviation (SD) as a percentage of the mean. The distribution is moderately right-skewed, characteristic of lognormally propagated uncertainty. The relatively narrow interval reflects the high data quality of the Small Wellness Facility’s hourly metered electricity and gas consumption, which together constrain the two dominant emission sources.

For the Large Wellness Facility, the 95% UI spans 4.23–5.96 kg CO_2_e around the baseline of 4.98 kg CO_2_e per 4.0-h visit (Monte Carlo mean 4.99 kg CO_2_e, SD = 0.44 kg CO_2_e, CV = 8.8%). The two facilities exhibit similar coefficients of variation (Small Wellness Facility 8.2%, Large Wellness Facility 8.8%), despite their difference in size, complexities and occupancies. At the Small Wellness Facility, the narrower CV reflects the high data quality of the two dominant metered sources (electricity and gas), which together constrain the output tightly. At the Large Wellness Facility, the diversification across many independent inputs provides partial variance cancellation, offsetting the higher individual CVs assigned to spend-based and fugitive categories.

The Spearman rank-correlation tornado diagrams in Fig. 7 reveal qualitatively different sensitivity structures within each facility. It measures how well the relationship between two variables holds by a monotonic function (i.e., whether they increase together even if not at a constant rate). At the Small Wellness Facility, the grid electricity emission factor remains the dominant driver of output uncertainty (Spearman ρ = 0.644), followed by the metered allocation denominator (ρ = –0.555) and electricity activity (ρ = 0.317). The allocation denominator’s strong negative correlation reflects the inverse relationship between occupancy estimates and per-visitor emissions: higher assumed occupancy distributes the same total emissions across more visitors, reducing the individual footprint. Notably, the inclusion of natural-gas data has moderated the dominance of the grid emission factor relative to an electricity-only model, while elevating the importance of the allocation denominator, which now governs the per-visitor distribution of a larger aggregate emission total.

At the Large Wellness Facility, the sensitivity landscape is more distributed. The additional-goods-and-services total (ρ = 0.559) and fugitive-emissions total (ρ = 0.464) emerge as the two most influential inputs, reflecting the high uncertainty CVs (40% and 50% respectively) assigned to these spend-based and leakage-based categories. The metered allocation denominator ranks third (ρ = −0.356), while the grid emission factor, natural-gas activity, and inbound-logistics total all contribute moderate sensitivities. This diffuse sensitivity profile is consistent with the Large Wellness Facility’s more balanced emission portfolio: reducing uncertainty in any single input will yield only incremental improvements in total-footprint precision, and meaningful uncertainty reduction will require simultaneous improvement across multiple data streams.

These results highlight a broader methodological insight: for facilities where metered energy dominates the GHG emission contributions, investment in higher-resolution grid emission factors (e.g., hourly rather than flat annual) and accurate occupancy denominators delivers the greatest return on uncertainty reduction. For diversified wellness facilities with large non-metered categories, improving the quality of spend-based and fugitive emission estimates is the priority, as these poorly constrained inputs collectively drive the majority of output variance.

### Interpretation

#### Case studies as contrasting applications

The two case studies provide complementary scenarios of operational greenhouse-gas drivers across contrasting spa and wellness contexts. The Large Wellness Facility case study provides a high-resolution characterisation of operational GHG drivers in a large, complex spa and wellness centre. The Small Wellness Facility represents a simpler operational profile: two metered energy sources, a single zone, and a visitor base an order of magnitude smaller. Together, they demonstrate that SPA-DEC’s allocation logic is transferable across facility types without modification to the core framework.

The Large Wellness Facility is a commercial thermal resort, whereas the Small Wellness Facility is a university sports park wellness zone operating under a fundamentally different business model, visitor profile, and service scope. Because the per-visit functional unit is not normalised for the level of service delivered within a visit, the resulting footprints reflect both operational efficiency and the breadth of amenities provided and should not be interpreted as a direct ranking of relative sustainability. Robust cross-facility benchmarking with SPA-DEC requires aligned system boundaries and comparable service definitions; the most directly comparable analyses demonstrated here are within-facility across time and season, and against the renewable-energy counterfactual, where boundaries are held identical.

Both case studies reinforce the broader expectation that spa and wellness facilities are energy-intensive by design, requiring sustained demands for heating, water pumping, and air handling. At both sites, Scope 1 and Scope 2 together account for the dominant share of the total impact, consistent with the operational profile of water-heated, climate-controlled leisure infrastructure. However, the two facilities exhibit markedly different scope distributions, reflecting their divergent energy mixes: the Small Wellness Facility’s simpler profile concentrates over 90% of impacts in Scopes 1 and 2, while the Large Wellness Facility’s geothermal and photovoltaic integration, combined with substantial Scope 3 procurement and logistics, produces a substantially more balanced three-scope distribution.

Atalay and Demir^13^ reported energy-related per-visitor footprints of ~8 kg CO_2_e for spa facilities in Turkey and Lithuania^13,14^. This excludes other operational emission sources. In contrast, the Large Wellness Facility’s mean per-visit footprint of ~5 kg CO_2_e is lower despite including additional categories like waste and staff commuting. This suggests that renewable integration can offset a large share of the energy burden that typically dominates spa footprints. At the same time, the Large Wellness Facility case study makes clear that non-metered sources are not negligible: fugitive emissions, logistics, and spend-based additional goods and services all contribute materially to the facility footprint: at the Large Wellness Facility, Scope 3 categories collectively account for over 30% of the total emissions. This provides an important caution against calculating environmental impacts solely through metered utilities.

The renewable energy scenario analysis, which compares observed 2024 emissions against a counterfactual grid and gas-only baseline, reveals how the dynamic allocation framework enables this kind of operational evaluation. The 32.9% emissions reduction attributable to geothermal and photovoltaic supply at the Large Wellness Facility is derived from the same metered consumption data that underpins the per-visitor results: because SPA-DEC tracks emissions by hour and by source, it is straightforward to substitute alternative emission factors for each energy stream and recalculate the per-visitor and aggregate outcomes. The counterfactual is not a standalone analysis but a direct output of the framework, demonstrating how the same infrastructure used for visitor-level attribution can also support facility-level decarbonisation planning.

#### Dynamic emissions accounting

SPA-DEC is dynamic in two senses. First, it is time-resolved: where metering exists, emissions are calculated at high temporal granularity and can vary by hour, day and season as demand, supply mix and operating conditions change. The hourly profiles presented in the Results show that relying on annual averages would mask substantial variation in emissions intensity. This matters because spa operations are characterised by strong seasonality and pronounced weekly patterns in occupancy and demand, and because decarbonisation interventions (e.g., operational changes, demand management, renewable utilisation) often target specific periods rather than annual totals.

Second, SPA-DEC is dynamic in its allocation logic. Per-visitor footprints are not derived from a single annual ratio, but from a structured attribution of emissions to visitor stays, recognising that spas accrue emissions both during opening hours (when services are being delivered) and during closed hours (when baseline requirements persist). This distinction is central in large facilities with substantial thermal demand and continuous support systems. By separating open-hour emissions (allocated per visitor-hour) from closed-hour overhead emissions (allocated per visitor), SPA-DEC avoids a known failure mode of naive hourly allocation, where very low occupancy periods can produce implausibly high per-visitor intensities and closed periods can either be ignored or implicitly misattributed. The approach therefore improves both interpretability and fairness: each visitor’s footprint reflects the operational conditions that prevailed during their stay and a transparent share of the facility overhead that enables the service to exist.

The value of the per-visitor dimension lies in three areas. First, it produces a functional unit, kg CO_2_e per visit, that is comparable across facilities, time periods, and ticket types, enabling benchmarking that aggregate totals cannot support. Second, it enables visitor-facing communication and, where required by emerging carbon offsetting mechanisms, visitor-level attribution at the point of exit. Third, it connects the intensity of a visit to the conditions under which it occurred, which is actionable information for both facility operators and informed visitors.

A further implication of this dynamic allocation is that it provides a direct bridge between facility operations and visitor-facing indicators. In many organisations, GHG accounting remains an annual compliance exercise disconnected from operational decision-making and visitor communication. SPA-DEC is designed to connect these domains: it preserves the integrity of whole-facility totals while producing per-visit metrics that can support internal benchmarking, scenario testing, and consumer communication.

#### Alignment with standards

SPA-DEC is intended to be compatible with widely used carbon accounting and LCA standards without replicating them in full. At the organisational level, the scope structure follows the GHG Protocol’s Scope 1–3 framework and aligns with ISO 14064 principles for inventory and emissions reporting. This matters for spa operators because electricity and gas are typically straightforward to report, but a substantial share of emissions may lie in value-chain categories (e.g., goods and services, logistics, fugitive emissions management, staff travel), and reporting requirements and stakeholder expectations are increasingly moving beyond Scopes 1 and 2. SPA-DEC provides a practical structure to incorporate these sources while maintaining traceability of how each category contributes to facility totals and per-visit outputs.

At the method level, SPA-DEC is informed by life cycle thinking (ISO 14040/44) in the treatment of indirect emissions and emission factors. However, SPA-DEC is not presented as a full multi-impact LCA of spa services. Its primary objective is to quantify the climate-change footprint of visits using greenhouse gas emissions expressed as kg CO_2_e, and the results reported here focus on GWP100: the most common characterisation metric used in organisational GHG emissions. This enables comparability with prevailing reporting practice and with policy and corporate targets framed in CO_2_e. A wider LCA can be valuable for spa facilities but extending to multi-impact assessment requires additional impacts, modelling choices and uncertainty treatment that are beyond the scope of a footprinting tool intended for broad operational uptake. SPA-DEC therefore occupies a complementary position, providing a transparent, scope-consistent climate indicator that can be implemented with existing operational data.

#### SPA-DEC strengths

SPA-DEC’s main contribution is a sector-specific, implementable method to convert whole-facility activity data into time-resolved, per-visit footprints, while retaining a transparent connection to scope-based reporting. This is important in a sector where the evidence base remains sparse and inconsistent, and where existing eco-labels and certification schemes do not consistently provide quantified, comparable carbon metrics. By explicitly mapping data sources to scopes and emission categories and by reporting outputs in a clear per-visitor functional unit, SPA-DEC supports benchmarking over time and comparison across facilities, as well as clearer internal decision support.

SPA-DEC balances comprehensiveness and usability. The approach can incorporate detailed metered flows when available, but it can also remain functional when certain categories are only available as annual totals or spend-based records. In the Large Wellness Facility case study, this is particularly relevant for “additional goods and services”, which captures procurement and services where physical quantities are not recorded in the required units. Recognising these categories is preferable to omitting them, as omission would bias results towards metered utilities and understate the role of procurement, maintenance and services in the overall footprint. At the same time, the explicit identification of spend-based components makes data-quality priorities visible: facilities can see where upgrading from spend proxies to quantity-based emissions would most reduce uncertainty and improve comparability. SPA-DEC is inherently modular, allowing facilities to include or omit emission categories based on their data availability and reporting requirements. This ensures the tool is tolerant to all data maturities.

The granularity of the outputs enables analysis that is difficult to obtain from conventional annual emissions. Hourly profiles support identification of emission-intensive time windows and can be used to evaluate operational interventions (e.g., reducing peak grid reliance, managing back-up heat demand, optimising renewable utilisation). The counterfactual renewable analysis demonstrates how the same framework can quantify avoided emissions from specific supply configurations while remaining grounded in metered activity data. When linked to carbon offsetting mechanisms, this level of detail is required^38^.

The two-facility inclusion demonstrates that SPA-DEC is transferable beyond large-format thermal spas. The core allocation logic applies equally to the simpler operational profile of the Small Wellness Facility. This suggests applicability to a broader class of visitor-economy service facilities, including leisure centres and aquatic facilities, and potentially to other service-based infrastructure where operational emissions must be attributed to individual users. Full adaptation to other contexts would require sector-specific inventory development, but the methodological framework does not present barriers to extension.

#### Limitations and boundary considerations

Several limitations should be considered when interpreting the results and the broader applicability of SPA-DEC.

##### Temporal scope

Both case studies draw on a single calendar year of operational data (2024). While the within-year temporal resolution is high, multi-year profiles would strengthen conclusions regarding seasonal patterns, longer-term decarbonisation trajectories, and the stability of non-metered category estimates. For the Large Wellness Facility, 2024 represents the first full year of operation including photovoltaic commissioning, which makes it the most appropriate available baseline but limits comparison with prior configurations. Extending the analysis to multiple years is a clear priority for future work.

##### Data completeness and quality

While SPA-DEC is designed to remain usable under heterogeneous data maturity, incomplete sub-metering and limited inventory detail can introduce uncertainty, particularly for Scope 3. Spend-based categories (notably additional goods and services) are sensitive to the choice and granularity of emission factors and to accounting classifications. These limitations are not unique to SPA-DEC; they reflect structural constraints in organisational data systems. The Monte Carlo analysis quantifies their contribution to output uncertainty and provides a direct guide to where data improvement efforts would be most productive. Nonetheless, they should be treated explicitly in reporting, and future work should prioritise improving procurement and maintenance inventories, as these categories can be material and are often poorly characterised.

##### Allocation assumptions and representativeness

SPA-DEC allocates shared facility emissions to visitors based on transparent rules that separate open-hour operational loads from closed-hour overheads. This improves interpretability and avoids pathological per-visitor estimates during low occupancy, but it does not fully resolve the inherent challenge that visitors do not consume resources uniformly. The results presented here assume homogeneous visitor behaviour within the defined visit duration. In practice, usage varies across features (e.g., sauna use versus lounging), and these differences may matter when the objective is personalised footprinting. Where access-control or feature-occupancy data exist, SPA-DEC could be extended to allocate portions of energy and water to activity classes or zones.

##### Multi-impact assessment

As noted above, the current framework reports GHG emissions only. For water-intensive facilities in particular, the absence of water use, eutrophication, and particulate matter results represents a meaningful gap in the environmental profile. Future work should develop the inventory base required to support multi-impact characterisation alongside the GHG results. A related avenue for future work is the explicit analysis of the energy-water-waste nexus at the visitor level. Examining how carbon KPIs correlate with peak water demand and seasonal waste generation could enable a more integrated reporting framework that recognises the interdependence of these operational flows rather than treating carbon as a standalone variable.

#### Implementation

SPA-DEC is designed to be implementable within day-to-day spa operations and to generate outputs that are meaningful to both facility managers and visitors. At the Large Wellness Facility, the most direct deployment pathway is a near-real-time footprint issued to visitors at the point of exit. Because the method draws on high-frequency metered consumption data and uses a time-based allocation approach, the footprint associated with a given stay can be calculated once a visitor’s entry and exit times are known and the corresponding operational emissions profile is available. In practice, this enables a receipt-style output (digital or printed) that reports the estimated kg CO_2_e attributable to the visit, together with a breakdown by major sources or scopes. Importantly, the footprint is dynamic: it reflects not only the duration of the stay, but also the conditions under which the visit occurred. For example, seasonal demand, the degree of reliance on grid electricity and gas back-up, and baseline overheads that must be allocated across visitors.

An alternative implementation is to estimate footprints in advance of a visit. This is feasible where the expected length of stay or ticket duration is known or can be predicted using historical patterns. In such cases, SPA-DEC can provide indicative footprints at the point of booking or entry, enabling visitors to make informed choices and allowing facilities to communicate the likely emissions implications of different ticket types or time windows. For example, facilities may choose to present a range for a given ticket duration, updated periodically as operational conditions and energy supply mixes change. While pre-visit estimates necessarily rely on forecasted rather than realised conditions, the same framework can be used to update the estimate post-visit using actual entry/exit times and realised metered consumption, ensuring consistency between prospective and retrospective reporting.

Beyond visitor-level communication, SPA-DEC’s scenario modelling capability positions the framework as a strategic planning tool for facility managers. By substituting alternative energy configurations into the same metered consumption framework, managers can evaluate the emissions implications of prospective operational changes before they are implemented. This includes assessing the impact of phased renewable integration, fuel switching, demand management interventions, or changes to operating hours. Rather than functioning solely as a retrospective reporting tool, SPA-DEC can therefore support proactive decarbonisation planning by quantifying the emissions benefit of specific transition pathways under realistic operational conditions.

Alongside this article, this research will provide access to an interactive results dashboard that allows users to explore the outputs generated by SPA-DEC and to examine the implications of the method for benchmarking and decarbonisation.

## Conclusions

This study introduces SPA-DEC as a sector-specific approach for quantifying the climate footprint of spa and wellness visits using a transparent, time-resolved allocation of facility emissions to individual stays. The methodology integrates high-frequency metered flows (energy and water) with annually normalised non-metered and value-chain inventories, allocating emissions across GHG Protocol Scopes 1–3 in alignment with ISO 14064 and ISO 14040/44. It distinguishes open-hour emissions, allocated per visitor-hour in proportion to occupancy, and closed-hour baseline overheads, allocated equally across all daily visitors. This open/closed distinction, combined with a full-cost attribution principle, ensures that no energy within the system boundary is left unattributed and preserves comparability across facilities. Applying hourly, source-specific emission factors, including a temporally resolved grid factor, rather than a flat annual average, the framework captures seasonal and intra-day variability that static approaches obscure.

Evaluations across two contrasting facilities confirmed the transferability of the core allocation logic. For example, in 2024, a typical four-hour visit at the Large Wellness Facility was associated with ~5 kg CO_2_e, and scenario analysis indicates that geothermal and photovoltaic supply reduced energy-related emissions by ~33% relative to a grid and gas only scenario. The Small Wellness Facility demonstrates that the same framework applies equally to a simpler, smaller-scale context, with a typical 1.5-h visit associated with ~3.5 kg CO_2_e and on-site PV reducing emissions by ~14%. SPA-DEC thus provides the sector with its first robust, standards-aligned per-visit metric, supporting benchmarking, hotspot-based decarbonisation planning, and near-real-time visitor communication and offsetting. By offering facilities and visitors the tools to measure, forecast, and act, this study contributes to a more accountable and decarbonised wellness sector. A key motivation behind this research is to help restore trust in sustainability claims across the wellness sector by establishing a transparent, science-based accounting methodology. In an industry with little existing standardisation, SPA-DEC offers a robust adaptable framework capable of functioning as a gold standard for emissions measurement. By enabling precise, per-visitor, near real-time emissions tracking, it promotes a shift away from annual reporting cycles toward continuous emission awareness.

To encourage open science and reproducibility, the authors of this study are adopting the following strategies. In this paper, the detailed methodology is disclosed – a comprehensive breakdown of data sources, assumptions, emission factors, and normalisation techniques used in the study, and the full temporal allocation algorithm and modular structure logic. Users of SPA-DEC are encouraged to share data, helping build a richer emissions profile database across spa types and regions. To aid this standardised reporting templates are provided, to encourage academics and spa facilities managers to align outputs with international standards like the GHG Protocol, supporting comparability and reproducibility. Feedback is invited from other researchers, facilities, and carbon accounting experts through workshops, webinars, or online forums taking place alongside the process of publication. The authors are open to collaborate with industry and academia to develop further case studies using SPA-DEC across different spa contexts.

## Methods

### Goal and scope definition

To comprehensively assess the carbon footprint of a spa visit, this methodology adopts the GHG Protocol framework^28^, categorising emissions into:

- Scope 1 – direct emissions from owned or controlled sources (e.g. gas combustion for pool heating),
- Scope 2 – indirect emissions from the generation of purchased electricity, heat, or cooling,
- Scope 3 – all other indirect emissions that occur in the value chain (e.g. purchased goods and services, business operations)

In accordance with ISO 14040, the goal of this analysis is to quantify the carbon footprint of a spa and wellness visit at the visitor level, supporting four intended applications: facility-level benchmarking and CSR reporting; operational decarbonisation decision-making through the identification of emission hotspots by source and time of day; visitor-level carbon communication and offsetting; and the development of a scalable, sector-wide accounting framework capable of deployment across diverse spa and wellness typologies.

The functional unit is one visit to a spa and wellness facility from entry to exit. The output of the calculator is a per-visitor carbon footprint expressed in kilograms of carbon dioxide equivalent (kg CO_2_e).

SPA-DEC quantifies greenhouse-gas emissions associated with the operation and service delivery of a spa and wellness visit over the period from visitor entry to exit. The boundary includes operational energy and water use (Scopes 1–2) and relevant upstream and supporting activities required to deliver the service (selected Scope 3 categories; e.g., consumables, waste management, staff travel and commuting, inbound logistics, and purchased goods and services). Visitor transport to and from the facility is excluded due to high variability and limited operator control and should be assessed using standalone methods.

Capital goods such as construction, major refurbishment and equipment replacement are not calculated by SPA-DEC at present because facility-specific inventories are rarely available in a consistent format. However, SPA-DEC endorses their inclusion where emissions estimates are already known from an external building LCA or EPD-based assessment. Such values can be entered as an annualised quantity and allocated per visitor for benchmarking. In line with ISO 14040/44 cut-off practice, sources expected to contribute less than 3% of the total service footprint may be excluded where justified and documented. For large facilities with long design lives and high visitor throughput, amortised construction-related emissions may be small at the per-visit level; nonetheless, boundary choices must be reported transparently to ensure comparability across facilities.

Food and beverage emissions are excluded from this methodology. Given the diverse and regionally specific nature of food-related emissions, these are more appropriately addressed through a standalone calculator.

The model is designed to be holistic and modular, allowing for flexibility in emissions reporting. Users can customise the scope by adding or removing specific emission sources based on their data availability and reporting needs. For benchmarking, facilities should compare results using aligned boundaries and comparable service definitions, and report clearly whether capital goods have been included. Because the per-visit functional unit is not normalised for the level of service delivered within a visit, comparisons across facilities with materially different service models should be interpreted with corresponding caution.

### Inventory data

To accommodate diverse facility capabilities and maintain flexibility, data inputs are categorised as either metered or non-metered.

- Metered inputs directly measure operational data, typically recorded through utility sub meters. These values are updated regularly or in real time and enable fine-grained allocation of emissions to specific time periods and visitor volumes. Supplementary Table S2 gives examples of metered consumption sources in spa and wellness facilities.
- Non-metered inputs are collected at a coarser temporal resolution, often annually. They are normalised on a per-visitor basis. Supplementary Tables S3, S4 give examples of non-metered input sources, split into visitor-related sources and business operation sources.

Although nearly all emission sources vary with visitor behaviour and occupancy, metered inputs are easier to measure regularly and typically represent the largest share of total emissions. Whereas, non-metered source per-visitor contribution is smaller and can be treated as annually aggregated and normalised emissions. This approach balances accuracy with practicality.

### Model overview

At its core, the SPA-DEC model follows a fundamental emissions calculation formula:

$${{\rm{E}}}=A\times {EF}\left({{{\rm{Equation}}}}\,1\right)$$

where *A* is activity data, such as energy consumption, and ${EF}$ is the emissions factor in kg CO_2_e per unit of activity data.

The total carbon footprint of a visitor’s spa visit is calculated as:

${{{\rm{CO}}}}_{2}{{\rm{e per visitor}}}={\sum}_{h\in {H}_{{visit}}}\frac{{E}_{h}}{{N}_{h}}+\frac{{E}_{d}^{{closed}}}{{N}_{d}}\,+{\sum}_{{{{\boldsymbol{V}}}}}\frac{{x}_{V}}{{N}_{A}}+{\sum}_{{{{\boldsymbol{B}}}}}\frac{{x}_{B}}{{N}_{A}}\left({{{\rm{Equation}}}}\,2\right)$Where ${E}_{h}$ is the total metered emissions from all sources $s$ during hour $h$, obtained using:

$${{{\rm{E}}}}_{h}={\sum}_{s}{A}_{s,h}\times {{EF}}_{s}\left({{{\rm{Equation}}}}\,3\right)$$

${H}_{{visit}}$ is the set of open hours that overlap with the visitor’s stay in the resort $\left[{t}_{0},{t}_{1}\right]$ on day $d$, and ${N}_{h}$ is the number of visitors present during hour $h$. The first term attributes open-hour emissions to the visitor in proportion to the occupancy of each hour.

${E}_{d}^{{closed}}$ are the total emissions accrued during closed hours on day $d$, allocated equally across ${N}_{d}$, the unique visitors recorded on that day.

${x}_{V},\,{x}_{B}$ represent non-metered visitor and business operation emissions for each category $V,B$. These emissions are measured annually and divided by the total number of annual visitors, ${N}_{A}$, to give the per-visitor impact.

The one-year aggregation period for non-metered inputs aligns with standard organisational GHG reporting cycles under the GHG Protocol and ISO 14064, captures the full seasonal cycle of visitor throughput, and smooths short-term procurement anomalies that would otherwise distort per-visitor estimates.

**Fig. 8:**
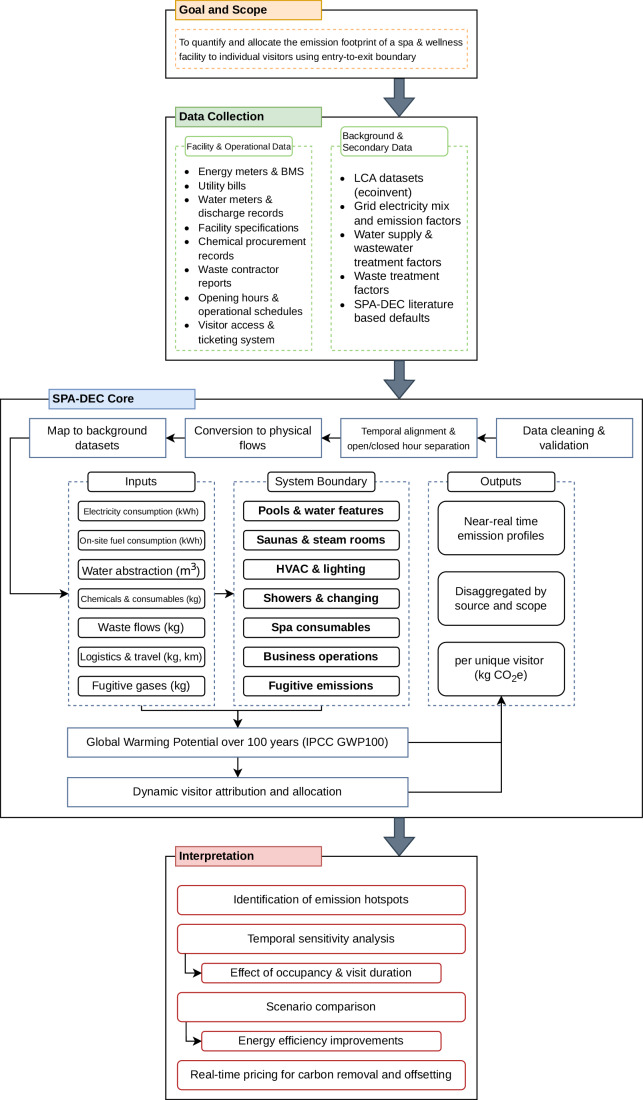
Workflow of the SPA-DEC methodology, from goal definition to interpretation. Schematic of the SPA-DEC framework organised as four sequential stages. The goal and scope stage sets the objective: to quantify and allocate a facility's emission footprint to individual visitors using an entry-to-exit boundary. The data collection stage assembles facility and operational data (for example, energy and water metering, chemical procurement and waste records, opening hours and ticketing) alongside background and secondary data (life cycle inventories, grid and treatment emission factors, and SPA-DEC literature-based defaults). The SPA-DEC core processes these inputs through data cleaning and validation, temporal alignment with open/closed-hour separation, conversion to physical flows, and mapping to background datasets; activity inputs are resolved within the system boundary (water features, saunas and steam rooms, HVAC and lighting, showers and changing, consumables, business operations and fugitive emissions), characterised using IPCC GWP100, and dynamically attributed to visitors to produce the per-visit outputs: near-real-time emission profiles, source and scope-disaggregated results, and a per-unique-visitor footprint (kg CO_2_e). The interpretation stage applies these outputs to emission hotspot identification, temporal sensitivity analysis, scenario comparison, and real-time pricing for carbon removal and offsetting. BMS building management system, GWP100 100-year global warming potential, PV photovoltaic.

Figure 8 is a flow diagram for the LCA process and core SPA-DEC model. The process begins with defining the goal and scope of the calculation: to quantify and allocate the emission footprint of a spa and wellness facility to individual visitors using a visitor entry-to-exit boundary. This is followed by the data collection (inventory analysis) stage, which feeds into the SPA-DEC core. The impact is assessed using IPCC GWP100 as the assessment method, followed by dynamic allocation to visitors. The results are then interpreted into per-visitor footprints with in-depth analysis for spa facilities to identify emission hotspots, understand temporal and seasonal variation, and conduct scenario analysis. Finally, the output can be linked to a carbon offset or removal mechanism. Because SPA-DEC attributes a specific, timestamped kg CO_2_e value to each individual visit, it provides the transaction-level granularity that offsetting mechanisms require^38^.

### Impact assessment: calculation methods

#### Energy and water consumption emissions

Where sub-meters are available, metered utilities are measured by feature. For each feature, energy and water-related emissions are calculated as follows:

4 $${E}_{{{\rm{energy}}}}={{\rm{kWh energy}}}\times {{EF}}_{{{\rm{energy}}},{{\rm{region}}}}$$

5 $${E}_{{{\rm{water}}}}={{\rm{litres used}}}\times {{EF}}_{{water}}$$

Where $E$ is the emissions and ${EF}$ is the emission factor for each source. Emission factors are region-specific and account for the local electricity grid’s energy mix. Each emission type is calculated over the duration of a visitor’s stay and then normalised by visitor count during that time.

For on-site renewable infrastructure such as photovoltaic and geothermal systems, ${EF}$ values represent lifecycle emission factors that amortise the embodied upstream emissions of the infrastructure over its expected energy output across the system lifetime. These factors are expressed in kg CO_2_e per kWh and are sourced from Ecoinvent.

#### Temporal allocation and visitor attribution

SPA-DEC allocates metered operational emissions to the functional unit by distinguishing open-hour service delivery from closed-hour baseline operation. For each day $d$ the facility schedule defines opening hours ${H}_{d}^{{open}}$ and closed hours ${H}_{d}^{{closed}}$.

**Step 1**. For each metered source $s$, hourly activity data ${A}_{s,h}$ are converted to emissions ${E}_{s,h}$ using source and region-specific emission factors.

Where metered data are available at a resolution coarser than hourly, SPA-DEC applies uniform temporal disaggregation, dividing interval totals equally across constituent hours. This is appropriate where the disaggregation window is short relative to the operational cycle, and the within-interval variation in consumption is small. Non-metered inputs, by contrast, are available only as annual aggregates and are treated as temporally uniform per-visitor constants rather than disaggregated to sub-annual resolution, for which no empirical basis exists.

**Step 2**. For $h\in {H}_{d}^{{open}}$, emissions are normalised by associated occupancy ${N}_{h}$ to obtain intensity in kg CO_2_e per visitor-hour:

6 $${I}_{h}=\frac{{E}_{h}}{{N}_{h}}$$

The visit component is obtained by summing ${I}_{h}$ across the hours of the visitor’s stay $[{t}_{0},{t}_{1}]$.

**Step 3**. Hours with anomalously high consumption during open-hours are identified using predefined outlier threshold: per-visitor consumption intensity exceeding three standard deviations above the facility mean for open-hour periods, consistent with standard practice for anomaly detection in environmental monitoring data. The emissions exceeding the threshold are capped and the excess is transferred into a daily closed-hour total, ${E}_{d}^{{closed}}$, which acts as an overhead. For skewed data, the threshold should be selected per facility. This prevents distortion of the open-hour visitor-hour intensity.

**Step 4**. For $h\in {H}_{d}^{{closed}}$, emissions are aggregated and added to the daily closed-hour total ${E}_{d}^{{closed}}$ and allocated equally across all unique visitors recorded on that calendar day. Hours straddling midnight are assigned to the calendar date on which they fall. For facilities where closed-hour energy and water consumption is demonstrably preparatory for the following day’s operation, allocation to the following day’s visitors is an optional alternative.

SPA-DEC adopts a full-cost approach to emissions attribution, consistent with the ISO 14040/44 principle that all inputs within the defined system boundary are attributed to the functional unit. Under this approach, 100% of open-hour energy consumption is attributed to visitors, as all energy consumed during opening hours is directly in service of delivering the visitor experience: pool heating, filtration, HVAC, lighting, and visitor-facing operations. There is no meaningful baseline to subtract during open hours: the facility’s operational state during opening hours is the service itself, and any attempt to separate a ‘baseline’ from ‘visitor-driven’ demand would be arbitrary and non-reproducible across facilities.

For closed hours, the same full-cost principle applies: all energy consumed outside opening hours is attributed to the visitors of that calendar day rather than left unattributed. The alternative marginal approach – treating closed-hour consumption as a fixed baseline and attributing only the excess to visitors – would leave baseline energy attributed to no functional unit, systematically understating the total service footprint and undermining comparability across facilities.

The decision to allocate closed-hour emissions equally across all visitors of the day, rather than weighted by visit duration, reflects the nature of the costs involved. Closed-hour overheads, such as pool heating, overnight water recirculation, and thermal storage maintenance, are fixed costs incurred independently of how long any individual visitor stays. Equal per-visitor allocation is therefore the methodologically appropriate choice: weighting by visit duration would imply that longer-staying visitors are responsible for a greater share of costs that may not, in practice, vary with their presence or duration.

#### Consumable, spa treatment, and cleaning & chemicals emissions

Non-metered inputs such as consumables or treatments are estimated per visitor using average quantities and product specific emissions factors obtained through Product Carbon Footprint (PCF) analyses (ISO 14040/44). For example, to estimate the per visitor emissions for a category of consumables (e.g. spa slippers), ${E}_{{{\rm{consumable}}}}$, the total quantity over the previous full year, ${Q}_{{{\rm{consumable}}}}$, is gathered or estimated and multiplied by the emissions factor per item, ${{EF}}_{{{\rm{consumable}}}}$ (kg CO_2_e/unit), which includes manufacturing, packaging, and end-of-life disposal. This value is normalised by dividing by total number of visitors over the same period, ${N}_{A}$ (Eq. (7)).

7 $${E}_{{{\rm{consumable}}}}=\frac{{Q}_{{{\rm{consumable}}}}\times {{EF}}_{{{\rm{consumable}}}}}{{N}_{A}}$$

The method is similar for treatments and cleaning. The quantity of treatment materials and cleaning chemicals are identified or estimated and multiplied by the emission factor per gram or litre and normalised to get to per-visitor emissions.

PCFs for items typically associated with a visit to a spa and wellness centre were compiled into an inventory^41–48^. The inventory was compiled using a structured, hierarchical approach:

1. Ecoinvent^49^ used as initial point of reference.
2. Peer-reviewed academic literature specific to the item.
3. Similar products with verified carbon footprint data.
4. Custom LCA models developed using Ecoinvent and SimaPro^50^.

Ecoinvent is a high-quality, consistent life cycle inventory consisting of the environmental impacts of products and services across various sectors^51^. The impact method used is IPCC GWP100 ^52^. It should be noted that these values are generalised estimates. Where item-specific carbon footprint data is available, it should be used in preference for increased accuracy. For some examples of common spa and wellness consumable item emission factors, see Supplementary Table S5.

#### Waste emissions

Waste-related emissions are attributed to each visitor based on normalised historical waste generation data. The emissions footprint from waste per visitor is calculated using region-specific emission factors corresponding to the type of waste, adhering to the GHG Protocol.

Where facilities maintain records of waste categories and treatment methods applied to each type, specific emission factors are applied^53^. Emission factors should primarily be drawn from regionally or nationally recognised inventories such as those of DEFRA^54^. If specific waste categories are unknown, then a general mixed-waste emission factor should be used as a proxy. If local background life cycle inventory data are unknown, then data should be used as default. Supplementary Table S6 shows the emission factors for waste categories, to be used when region-specific factors are unknown. When waste categories are unknown, facilities should use the value for municipal waste, incineration (0.516 kg CO_2_e per kg waste)^55^.

In any case, the total emissions from spa-generated waste in the previous full year are divided by the total number of visitors during that same period (Eq. (8)). This per-visitor emission estimate is then treated as a static value and added as a constant to the overall footprint calculation. Treating the per-visitor waste footprint as an annually normalised constant balances methodological rigour with practical data requirements and accommodates facilities where waste data are available only at annual resolution.

8 $${E}_{{{\rm{waste}}}}=\frac{\sum \left({Q}_{{{\rm{waste category}}}}\times {{EF}}_{{{\rm{waste category}}}}\right)}{{N}_{A}}$$

Where data availability improves over time, the waste footprint component can be calculated over extended time periods to reflect more precise visitor level allocation by category.

#### Business operation emissions

Business operation emissions encompass a range of indirect (Scope 3) activities that support the delivery of spa and wellness services^35^. The SPA-DEC is modular, allowing facilities to include or exclude business operation categories depending on their data availability and reporting requirements. However, for facilities seeking to align with LCA ISO 14040/44, GHG Protocol Scope 3 guidance, or upcoming regulatory instruments such as the EU GCD, the inclusion of business operation emissions is recommended. SPA-DEC incorporates a methodology for business operations for ease of use for facilities, avoiding the need for more than one calculation platform.

However, relevance is a critical principle when accounting for business operations. Only emissions directly or indirectly linked to the delivery of the spa and wellness experience should be included. Business activities unrelated to spa operation – such as those included in Supplementary Table S7 – must be excluded. SPA-DEC is a visitor-focused emissions tool, and as such, it does not function as a full organisational inventory.

As a guiding principle: if the emission source would not exist without the spa facility operating and hosting visitors, it is likely to be within the scope of SPA-DEC. Facilities are encouraged to document boundary decisions and assumptions clearly when completing their SPA-DEC input.

The calculation method for each business operation emission category is outlined below. For all categories, annual emissions are converted into a per-visitor footprint as:

9 $${E}_{{{\rm{per visitor}}}}=\frac{{A}_{B}\times {{{\rm{EF}}}}_{{{\rm{B}}}}}{{N}_{A}}$$

Where ${A}_{B}$ is the annual business operation activity, ${{EF}}_{B}$ is the related emissions factor, and ${N}_{A}$ is the annual number of visitors.

##### Company transport

This is scope 1 fuel consumption from any spa-owned vehicles used in the delivery of visitor services. Emissions are calculated using fuel consumption records or mileage data multiplied by fuel emissions factors. For air travel, a robust flight emissions calculator is used. For remaining forms of transport, UK BEIS emission factors are used^56^.

##### Inbound logistics

Covers the scope 3 transport of goods into the facility by third-party suppliers, such as delivery of consumables. Calculations are based on frequency, distance, and transport mode, or on weight-distance emission factors from UK BEIS^56^.

##### Business travel

Includes scope 3 employee travel for operational purposes, based on travel logs or expense reports, using distance-based factors for rail or car travel. The emissions calculation and factors are similar to company transport^56^.

##### Company accommodation

Scope 3 overnight stays for staff conducting relevant business travel. The emissions are typically estimated using nights stayed and a relevant emissions factor per room night, based on hotel region and standard^56^.

##### Staff commuting

This item refers to scope 3 emissions from employees travelling to and from the facility. Where possible, data should be collected via staff surveys identifying the average commute distance, mode of transport, and frequency. If survey data is unavailable, standard assumptions may be applied based on location and employee count. Travel emission calculation and factors similar to company transport.

##### Fugitive emissions

These are scope 1 emissions from refrigerant leaks and gas losses from HVAC or spa equipment, calculated based on type, quantity, and GWP. Leakage rates can be estimated from equipment specifications or maintenance logs^57^. Emission factors come from IPCC AR5 GWP100^52^.

##### Additional goods and services

Includes emissions that haven’t already counted from the procurement of services and non-durable goods that support operations – such as third-party cleaners or digital services. These additions are estimated using spend-based emission factors drawn from environmentally extended input-output databases, such as EXIOBASE^58^. This section can also be used for above categories when the required units are unknown, but the cost is. Although this is a fallback option, it ensures that no emissions go uncounted.

## Supplementary information

Transparent Peer Review file

Supplementary Materials

## Data Availability

The aggregated data underlying all figures in this study are openly available on Figshare at 10.6084/m9.figshare.32825969^59^. These data are also bundled in the software archive^60^ (see Code Availability) so that the figures can be regenerated directly. Source Data for the main figures are provided with this paper. The raw inputs to the model are commercially sensitive; they are therefore not publicly available. Facility identities are not disclosed, and the released tables are aggregated to a level from which the underlying hourly series cannot be reconstructed (see the data dictionary for what is and is not included).

## Materials availability

SPA-DEC is a computational tool. This study did not generate new biological materials or reagents. For the purpose of open access, the authors have applied a Creative Commons Attribution (CC BY) licence to any Author Accepted Manuscript version arising from this submission.

## Code availability

The SPA-DEC model, the figure-generation scripts, and the interactive dashboard are written in Python (3.11+; NumPy, pandas, Matplotlib, Plotly, Streamlit) and released as open-source software under the GNU Affero General Public License v3.0. The source code is archived at Zenodo with the version-specific 10.5281/zenodo.21038901^60^. The repository includes the model library, the scripts that regenerate the figures from the aggregated outputs, a fully synthetic example dataset that allows the complete pipeline to be run without access to the proprietary facility data, a results visualisation dashboard, and the per-facility configuration choices used for the two case studies and the reasons for them. Running the released code with the case-study configurations and the underlying facility data reproduces the per-visit results reported here.
