# Supplementary material for: Spa visits carry measurable carbon footprints that vary with energy use and renewables
Source: Commun Sustain. 2026 Jul 22;1(1):116. doi: 10.1038/s44458-026-00122-x (PMC13391376; doi:10.1038/s44458-026-00122-x)
Supplement: Supplementary file 2 — Supplementary Materials [file 44458_2026_122_MOESM2_ESM.docx]

**Spa visits carry measurable carbon footprints that vary with energy use and renewables**

***Supplementary Materials***

Robert Hanea, Finn McFall, Xavier Font, Jonathan Chenoweth, Ionut Corduneanu, Eduard Goean, Dabo Guan, Lorenzo Fioramonti, Jhuma Sadhukhan

# Supplementary Tables

Table S1: Per-input coefficients of variation (CVs) used in the Monte Carlo uncertainty analysis, assigned by structured expert judgement based on data provenance. Each input is modelled as a unit-mean lognormal multiplier.

| Emission input | Tier | Small Facility | Large Facility | Basis |
| --- | --- | --- | --- | --- |
| Occupancy / allocation denominator | Metered activity | 5% | 5% | Visitor counts logged hourly; denominator well constrained |
| Electricity activity | Metered activity | 5% | 5% | Purchased electricity metered hourly |
| Natural gas activity | Metered activity | 5% | 5% | Consumption logged hourly |
| Water supply activity | Metered activity | 5% | - | Volumes metered hourly |
| Wastewater activity | Metered activity | 5% | 5% | Volumes metered hourly |
| Photovoltaic generation (allocated) | Metered activity | 5% | 5% | Allocated PV generation modelled hourly |
| Geothermal activity | Metered activity | - | 5% | Geothermal energy recorded hourly |
| Grid electricity emission factor | Secondary EF | 10% | 7.5% | Secondary carbon intensity data; Large facility uses hourly national factors (tighter), Small uses a flat annual factor |
| Natural gas emission factor | Secondary EF | 5% | 5% | Combustion factors comparatively standardised |
| Water supply emission factor | Secondary EF | 10% | - | Secondary conversion factor |
| Wastewater emission factor | Secondary EF | 10% | 10% | Secondary treatment conversion factor |
| Photovoltaic embodied emission factor | Secondary EF | 20% | 20% | Lifecycle embodied factor from secondary literature; less precise than operational measurement |
| Geothermal emission factor | Secondary EF | - | 15% | Secondary lifecycle intensity, more uncertain than the activity |
| Waste | Annual estimate | 20% | 20% | Annual aggregates with default disposal factors |
| Consumables | Annual estimate | 30% | 30% | Annual engineering estimates, blended default factors |
| Company transport | Annual estimate | 30% | 30% | Annual use estimate, not continuous monitoring |
| Staff commuting | Annual estimate | 30% | 30% | Inferred annual travel pattern / assumed distances |
| Inbound logistics | Annual estimate | 35% | 35% | Delivery counts, distances and modes from sparse annual records |
| Business travel | Annual estimate | - | 30% | Annual estimate with mode/trip assumptions, not trip-level records |
| Company accommodation | Annual estimate | - | 30% | Estimated from annual aggregates and generic factors |
| Additional goods & services | Spend-based | 40% | 40% | Spend-based modelling using EXIOBASE input–output factors on procurement spend |
| Fugitive refrigerant emissions | Fugitive | 50% | 50% | Small leakage-mass errors translate to large CO_2_e changes; refrigerant losses inherently uncertain |

*Table S2: Metered consumption sources in spa and wellness facilities.*

| Feature ($\boldsymbol{f}$) | Description | Consumption Types |
| --- | --- | --- |
| Heated pools and whirlpool baths | Water heating, pumps, filtration | Electricity, gas, geothermal, water |
| Cold plunge pools | Cooling and filtration | Electricity, water |
| Saunas (infrared, dry) | Electrical or wood-burned heating | Electricity, wood |
| Steam rooms | Steam generation, ventilation | Electricity, gas, water |
| Showers and changing facilities | Water heating, ventilation | Electricity, gas, water |
| Heating, ventilation, and air conditioning (HVAC) systems | Climate control | Electricity, gas |
| Lighting and audio systems | LED, halogen, decorative lighting | Electricity |
| Interactive water features | Slides, jets, wave pools | Electricity, water |
| Laundry and towel services | Washing, drying, pressing | Electricity, gas, water |
| Standby energy | Energy consumption outside of facility operating hours | Sub-metered or estimated from utility data |
| Standby water | Water consumption outside of facility operating hours | Sub-metered or estimated from utility data |

*Table S3: Non-metered visitor-related sources.*

| Category ($\boldsymbol{f}$) | Description |
| --- | --- |
| Consumables | Slippers, robes, disposable cups |
| Spa treatments | Massage oils, wax, aromatherapy |
| Cleaning and chemicals | Disinfectants, soaps, room cleaning |
| Waste | Packaging, food waste, hygiene products |

*Table S4: Non-metered business activities crucial to the running of the facility.*

| Category ($\boldsymbol{B}$) | Description | Data Required |
| --- | --- | --- |
| Company transport | Fuel use for owned delivery or service vehicles | Vehicle mileage data, common route estimates |
| Inbound logistics | Delivery of goods by third party | Supplier data, delivery frequency |
| Business travel | Travel to relevant operational meetings or trainings directly related to spa management or service delivery | Expense reports |
| Company accommodation | Hotel stays during staff training or spa-related site visits | Expense reports |
| Staff commuting | Employee transport to and from work to deliver spa services | Staff surveys or estimates |
| Fugitive emissions | Refrigerant or gas leaks | Equipment labels and maintenance logs |
| Additional goods and services | One-off purchases, services that support spa operations | Procurement data |

Table S5: Emission factors of common spa and wellness consumable items.

| Product | Category | Unit | Emission factor (kg CO₂e) | Source |
| --- | --- | --- | --- | --- |
| Aromatic oils | Treatment | per use | 15.15 | CarbonCloud 2025 ^1^ |
| Massage oil | Treatment | per use | 3.1 | Schmidt 2015 ^2^ |
| Facial oil | Treatment | per use | 10.375 | Glew 2014 ^3^ |
| Body exfoliating scrub | Treatment | per use | 2.4 | Natural salt scrub LCA |
| Hand soap | Cleaning | per use | 0.314 | Villota-Paz 2023 ^4^ |
| Toilet cleaner | Cleaning | per kg | 0.03 | Ecoinvent ^5^ |
| Reusable cleaning cloths | Cleaning | per kg | 4.49 | Ecoinvent ^5^ |
| Surface disinfectant | Cleaning | per kg | 0.327 | Ecoinvent ^5^ |
| Glass cleaner | Cleaning | per kg | 0.327 | Ecoinvent ^5^ |
| Laundry detergent | Cleaning | per kg | 1.86 | Powder detergent LCA |
| Pool cleaning chemicals | Cleaning | per kg | 0.974 | Chlorine LCA |
| Salt pool concentrates | Cleaning | per kg | 1.5 | Ecoinvent |
| pH balancing agent - acid | Cleaning | per kg | 0.910 | Muriatic acid LCA |
| pH balancing agent - base | Cleaning | per kg | 0.408 | Soda ash LCA |
| COVID-19 Test kits | Cleaning | per use | 0.09 | Courdier et al. ^6^ |
| Slippers | Single use | per item | 0.176 | Slippers LCA |
| Tissues | Single use | per kg | 2.78 | Tissue paper LCA |
| Gloves | Single use | per pair | 0.068 | Hartalega 2024 ^7^ |
| Bathrobe | Reusable textile | per item | 14.68 | Gungor 2009 ^8^ |
| Towel | Reusable textile | per kg | 9.43 | Towel LCA |

Table S6: Emission factors for the treatment of waste categories, taken from Ecoinvent v3.1.1 ^5^.

| Waste Category | Waste Treatment | Emission Factor (kg CO_2_e per kg) |
| --- | --- | --- |
| Municipal waste | Incineration | 0.516 |
|  | Landfill | 0.0307 |
| Paper and cardboard | Incineration | 0.0209 |
|  | Landfill | 0.00943 |
|  | Recycling | 0.0 |
| Plastic | Landfill | 0.0453 |
|  | Recycling | 0 |
| Organic | Composting | 0.0293 |
|  | Incineration | 0.035 |
| Glass | Landfill | 0.0045 |
| Hazardous waste | Incineration | 2.43 |
| Wastewater (greywater) | n/a | 0.0005 |

Table S7: Emissions unrelated to the delivery of visitor services and excluded from the SPA-DEC scope.

| Category ($\boldsymbol{B}$) | Examples |
| --- | --- |
| Corporate travel unrelated to spa operations | Executive flights for investor meetings, non-operational strategy workshops |
| Expansion and development activities | Site visits for planning new branches, emissions from architectural design work |
| Emissions from separate business divisions | Back-office admin offices, R&D centres |
| Marketing activities | Emissions from marketing campaigns |
| Office or admin energy not linked to spa experience | Headquarters emissions unless serving the facility directly |
| Outbound logistics | Shipment of products to external visitors |

# Large Wellness Facility Case Study Set up

The Large Wellness Facility, situated in Romania, stands as one of the world’s largest thermal wellness facilities, encompassing over 30,000 square metres of indoor space and accommodating a substantial number of visitors annually. The resort boasts extensive amenities, including multiple thermal pools, saunas, and the country’s most expansive indoor botanical garden, featuring over 800,000 plants. The resort is a LEED Platinum certified building meaning they have implemented advanced sustainability measures. Geothermal heating is the primary thermal energy source, supplemented by gas boilers during peak loads and cold spells. Waste heat is recovered via heat exchangers. On the electrical side, the Large Wellness Facility has invested in on-site photovoltaic generation, which supplies a significant share of the resort’s power demand. The remaining electricity is procured from the grid. In terms of water, the pools operate on a closed-loop water cycle with state-of-the-art treatment. Natural thermal water is purified and ozone-filtrated to spa quality. After use, the water is rigorously reprocessed – again including ozone filtration – and pH is naturally optimised without the use of chemical additives. The entire pool volume is recirculated about four times a day and as a result, net water losses are minimal with 90.1% of water recycled.

## Data Collection and SPA-DEC Implementation

A comprehensive data inventory covering the Large Wellness Facility’s operations was gathered to serve as input to the SPA-DEC. The key data points required are visitor numbers, energy, water, waste, and consumables & operations.

**Visitor Numbers**

Visitor count is recorded on an hourly basis via the entry turnstile and electronic wristband system. This provided a highly granular profile of occupancy and facility usage over time. Summer months see the highest average visitor counts, peaking in August. More prominent is the weekly differences – weekends see far more average visitors than weekdays. This temporal change is captured by SPA-DEC, which allows emissions from energy and water use to be allocated per hour and per visitor dynamically rather than assuming a flat average load.

**Energy Data**


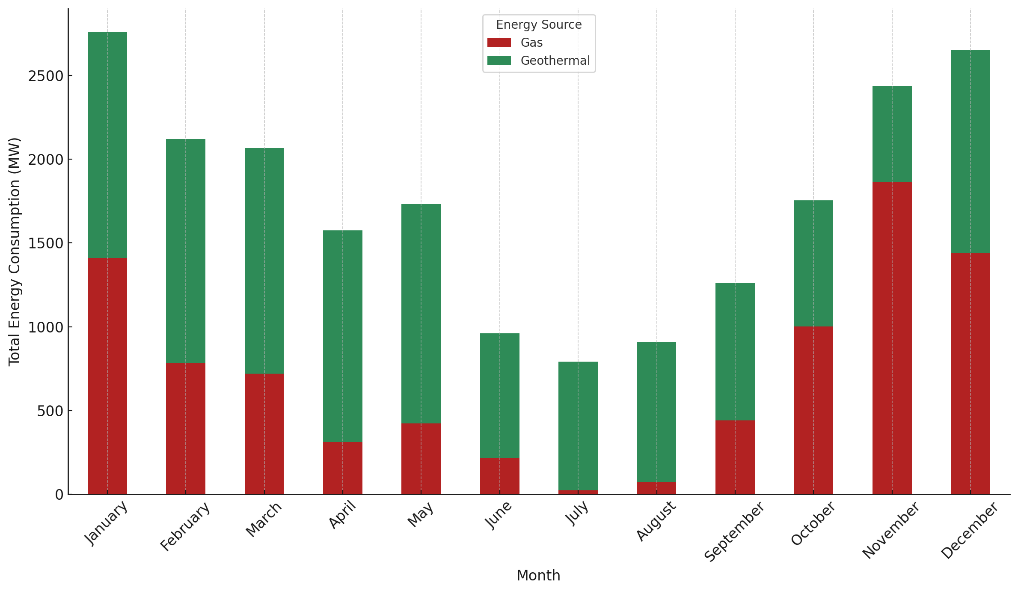
Energy consumption data was collected from facility meters and management records. The Large Wellness Facility’s geothermal system and gas boilers are sub metered, providing hourly thermal energy delivery. As expected, gas use is highly seasonal, rising in winter and near-zero in summer, whereas geothermal provided a steady baseload year-round. Figure S1 shows the monthly breakdown of geothermal and gas consumption in 2024.

Figure S1: Monthly geothermal and gas consumption in 2024.


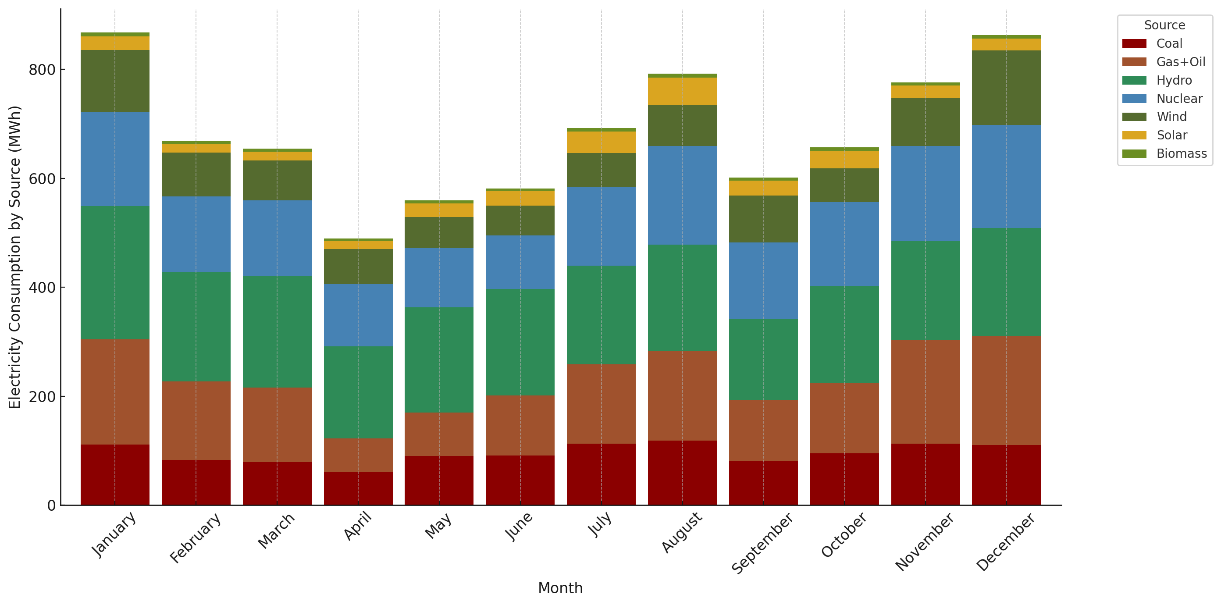
Electricity is also tracked, with the PV becoming fully operational in early 2024. During day-light hours the PV production can occasionally surpass the facility consumption. At this point the Large Wellness Facility will either store the energy locally or provide this energy to the national grid. The grid is used in return, when the PV production does not cover the facility’s consumption. Grid energy data is sourced from Transelectrica ^9^. The energy split of the Large Wellness Facility’s grid energy usage in 2024 can be seen in Figure S2. Hydropower makes up the largest portion of grid energy data, followed by hydrocarbon and nuclear. For the Large Wellness Facility case study, the most accurate energy split, and relevant emission factors were established using a combination of Transelectrica and Ecoinvent. Transelectrica provides a granular hourly energy split, while Ecoinvent provides Romanian-specific emission factors for each source.

Figure S2: The Large Wellness Facility electricity consumption from grid, split by source, over 2024.

**Water Consumption**


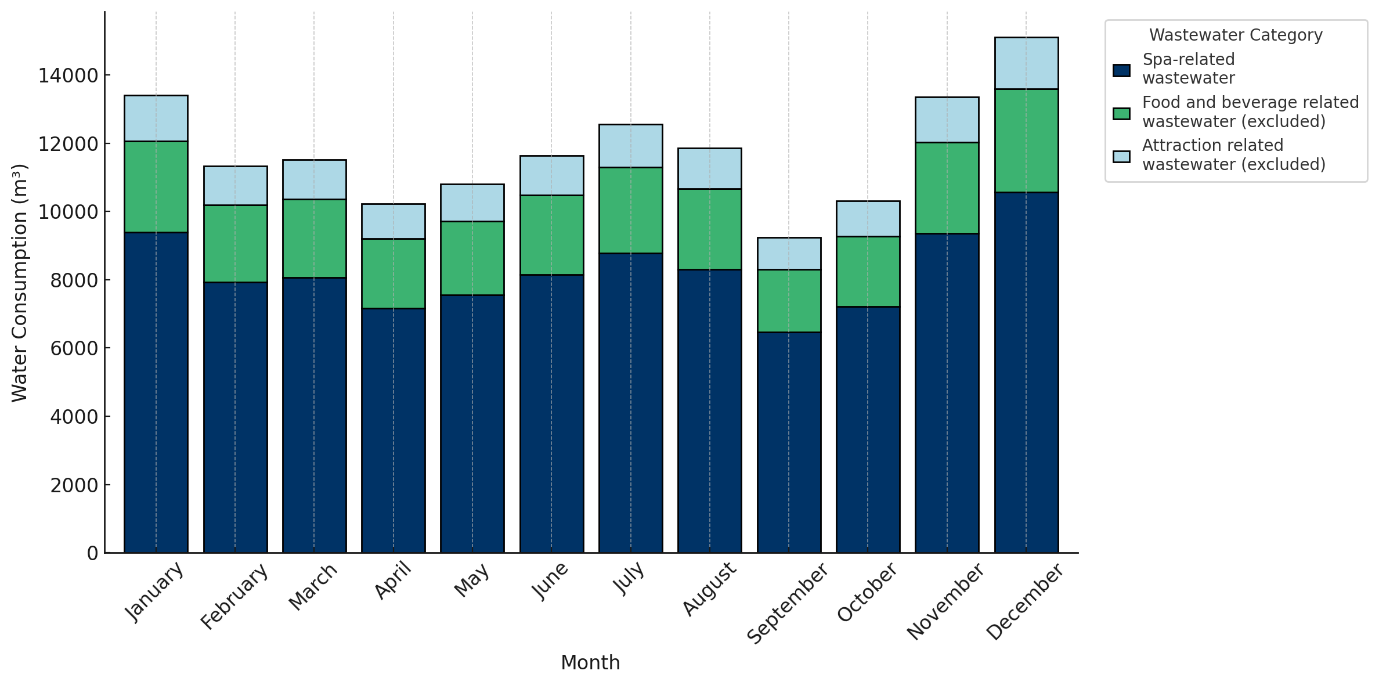
The Large Wellness Facility’s water extraction and filtration system is state of the art. They have their own on site bore holes supplying geothermal and fresh water, with 90.1% of water recycled, cleaned at their water plant and eventually deposited in their onsite lakes, warranting no associated water-related emissions footprint. The energy consumption from the bore holes and water plant is accounted for in the metered energy consumption. The remaining 9.9% of wastewater is sent to the city wastewater treatment centre. A Romanian municipal wastewater emissions factor from Ecoinvent is used to calculate any emissions associated with this treatment. For the sake of this case study, it is split into three categories, shown in Figure S3. Roughly 70% of wastewater is related to spa operation.

Figure S3: Wastewater at the Large Wellness Facility in 2024.

**Overnight and Anomalous Energy and Water Consumption**

Excess heat overnight is stored in boilers, and the water is constantly heated. This allows for a lower extraction of geothermal water and less energy required to power the geothermal pumps. Furthermore, it negates the need to heat the vast amount of water from ambient temperature. To account for baseline facility operations outside public hours, SPA-DEC includes a dedicated treatment of overnight energy and water consumption. These closed-hour emissions cover the periods from 00:00 to 08:00 each day. Hourly consumption data is used to isolate these overnight values. These numbers are normalised across the total number of visitors that day.

Anomalous spikes in open-hour energy or water use were also identified using outlier detection thresholds. The excess emissions were then reclassified into the closed-hour emissions.

**Waste Generation**

Waste data was collected as aggregated values for 2024. In 2024, the facility generated a total of 805 tonnes of solid waste related to spa facility operation, including 392.1t of mixed municipal waste, 58.6t of recyclable paper and cardboard, 24.5t of medical and hygiene waste, and 311.3t of construction debris. Each waste stream was entered into SPA-DEC with an appropriate disposal method and emission factor. Where exact emission factors were unknown, SPA-DEC’s default emission factors sourced from Ecoinvent were used ^10^.

**Consumables and Operations**

All data inputs relevant to the visit of a guest to the resort were accounted for. Operational categories like staff commuting and business travel were estimated based on company records and assumptions – for instance, an employee survey provided the modal split and average distance of staff commutes, which SPA-DEC converted to emissions using DEFRA and UK BEIS emission factors ^11,12^. The Large Wellness Facility management was able to provide extensive data on the quantities of all spa-related consumables used in 2024, such as essential oils and sauna aromas. A few consumable quantities in terms of mass were unknown. However, the SPA-DEC “Additional Goods & Services” module allowed expenditure-based inputting. Staff business travel related to spa operation was measured using an internal emissions calculator for air travel passengers ^13^. The dynamic calculator provides highly accurate, comprehensive emission footprints for flights. It covers the full LCA of a flight, includes non-CO_2_ impacts such as NO_x_, H_2_O, and contrail-induced cirrus clouds, and uses historical adjustment factors to improve precision on pre-flight estimates ^13^.

The process of data collection was eased by the SPA-DEC Master Spreadsheet, which simplifies the categorisation process and aligns the data correctly to run the calculator. With the dataset completed, SPA-DEC was applied to compute the Large Wellness Facility visitor emissions. The Master Spreadsheet is imported into the SPA-DEC Python and Streamlit-based dashboard. The tool then allows the user to input their date and time of entry and exit and outputs an emissions footprint.

# Small Wellness Facility Case Study Set Up

The Small Wellness Facility is a university-operated sports and leisure complex located in the south of England, offering a broad range of fitness, aquatic, and wellness amenities to students, staff, and the local community. The wellness zone – comprising a 50-metre Olympic-standard swimming pool, sauna, steam room, and changing facilities – forms the system boundary for this case study. The facility is actively progressing towards net zero in line with its host institution's 2030 decarbonisation commitment and holds a FISU Platinum Healthy Campus certification in recognition of its contribution to student and community wellbeing. On the energy side, the facility recently commissioned a large rooftop photovoltaic array, generating over 728 MWh of electricity annually and supplying a significant share of the building's energy needs. The university's energy team has also deployed a sub-metering platform across the building, providing zone-level energy monitoring that supported the data collection for this study.

## Data Collection and SPA-DEC Implementation

A data inventory covering the wellness zone's operations was assembled to serve as input to SPA-DEC. The full framework was applied across Scopes 1, 2, and 3. Given the smaller scale and lower operational complexity of this facility relative to the Large Wellness Facility, several annual aggregate inputs required estimation with the support of facility management, and some Scope 3 category values were allocated proportionally to the wellness zone from whole-facility records.

**Visitor Numbers**

Visitor counts for the wellness zone were derived from a combination of membership records, booking system data, and poolside manual counts recorded by facility staff. Hourly visitor profiles were estimated from these aggregated figures using knowledge of facility opening hours, seasonal attendance patterns, and scheduled closures. While less granular than the turnstile-and-wristband system used at the Large Wellness Facility, this approach provided a sufficient basis for dynamic hourly allocation and is representative of the data infrastructure available at mid-scale leisure facilities.

**Energy Data**

Energy consumption data were drawn from the facility's sub-metering system, which records electricity and gas consumption at zone level, including dedicated meters for the pool, changing facilities, and PV generation. This zone-level sub-metering, supported by the university energy team's monitoring platform, enabled the wellness zone's energy profile to be isolated from the broader building. Grid electricity and on-site gas constitute the two primary metered energy sources. The PV system, commissioned in 2024, supplies a material share of the facility's electricity demand, with surplus generation exported to the grid. UK grid emission factors from DEFRA and UK BEIS were applied to purchased electricity, and the PV system's embodied lifecycle emissions were accounted for using an Ecoinvent emission factor.

**Water Consumption**

Water consumption was sub-metered across grey water and pool water circuits. All wastewater is directed to the municipal treatment network, and a UK-specific wastewater emission factor from Ecoinvent was applied to calculate associated emissions. Unlike the Large Wellness Facility, the Small Wellness Facility does not operate an on-site water treatment plant; pool water is managed through standard chemical treatment and filtration systems, and net water losses are characteristic of a conventionally operated public swimming facility.

**Waste Generation**

Waste data were collected as annual aggregated totals for the facility as a whole. Where waste streams were not disaggregated by zone, the wellness zone's share was estimated in consultation with facility management based on relative usage and staffing levels. Waste categories and treatment methods were entered into SPA-DEC with appropriate emission factors drawn from Ecoinvent and DEFRA guidance.

**Consumables and Operations**

Non-metered operational data, including staff commuting, business travel, consumables quantities, and additional goods and services, were collected as annual totals, with some values estimated with the assistance of facility management where precise records were unavailable. DEFRA and UK BEIS emission factors were used for transport-related categories. The SPA-DEC Master Spreadsheet was used to structure the data collection and ensure consistent categorisation across all Scope 3 inputs, enabling direct comparison with the Large Wellness Facility outputs.

# References

1 CarbonCloud. Essential oil from peppermint, Europe. (2025).

2 Schmidt, J. H. Life cycle assessment of five vegetable oils. *Journal of Cleaner Production* **87**, 130–138 (2015).

3 Glew, D. & Lovett, P. N. Life cycle analysis of shea butter use in cosmetics: from parklands to product, low carbon opportunities. *Journal of Cleaner Production* **68**, 73–80 (2014).

4 Villota-Paz, J. M., Osorio-Tejada, J. L. & Morales-Pinzón, T. Comparative life cycle assessment for the manufacture of bio-detergents. *Environmental Science and Pollution Research* **30**, 34243–34254 (2023).

5 Wernet, G. *et al.* The ecoinvent database version 3 (part I): overview and methodology. *The International Journal of Life Cycle Assessment* (2016).

6 Courdier, S. *et al.* The direct emissions related to Global Warming Potential of different types of diagnostic tests at different phases of the COVID pandemic: A climate-focused life-cycle assessment. *PLOS Climate* **4**, e0000561 (2025).

7 Murugiah, S. Hartalega discloses carbon footprint of glove products. *The Edge Malaysia* (2024).

8 Güngör, A., Palamutçu, S. & İkiz, Y. Cotton textiles and the environment: Life cycle assessment of a bathrobe. *Textile and Apparel* **19**, 197–205 (2009).

9 Transelectrica, S. A. SEN‑Grafic: CARB. (2025).

10 Frischknecht, R. *et al.* The ecoinvent database: overview and methodological framework (7 pp). *The international journal of life cycle assessment* **10**, 3–9 (2005).

11 Department for Environment Food & Rural Affairs & Department of Energy and Climate Change. Guidelines to Defra/DECC's GHG conversion factors for company reporting. *DEFRA, London* (2011).

12 Department for Energy Security and Net Zero. Greenhouse gas reporting: conversion factors 2024. (2024).

13 McFall, F. *et al.* Aviation passenger carbon footprint calculator with comprehensive emissions, life cycle coverage, and historical adjustment. *Communications earth & environment* **6**, 855 (2025).
