# Supplementary material for: Spa visits carry measurable carbon footprints that vary with energy use and renewables
Source: Commun Sustain. 2026 Jul 22;1(1):116. doi: 10.1038/s44458-026-00122-x (PMC13391376; doi:10.1038/s44458-026-00122-x)
Supplement: Supplementary file 1 — Transparent Peer Review file [file 44458_2026_122_MOESM1_ESM.pdf]

nature portfolio

Peer Review File

Spa visits carry measurable carbon footprints that vary with energy use and renewables

Corresponding Author: Mr Finn McFall

This file contains all reviewer reports in order by version, followed by all author rebuttals in order by version.

Version 0:

Reviewer comments:

Reviewer #1

(Remarks to the Author)
This manuscript introduces SPA-DEC, a dynamic emissions calculator applied to spa and wellness facilities, and represents one of the first sector-specific operational LCA frameworks for this industry. The allocation approach — dividing emissions between visitor-hours and closed-hour maintenance — is methodologically intuitive and a genuine contribution to the field. The case study is well-executed, and the writing is clear throughout.

However, several concerns should be addressed before the work can be considered for publication.

- Major Comments

The manuscript contains notable inconsistencies in greenhouse gas scope classification that undermine the methodological rigour of the framework. Fugitive emissions are misclassified: these constitute Scope 1 emissions, not Scope 2. On-site photovoltaic systems, where equipment is purchased rather than leased, are more appropriately categorised under Scope 3 (capital goods) rather than Scope 2. Similarly, company transport is consistent with Scope 3 commuting. These misclassifications recur in the discussion of Figure 2 and should be corrected systematically throughout.

The novelty of SPA-DEC lies primarily in its allocation criteria and dynamic temporal resolution; however, this methodological contribution is underexplored relative to the case study results. The manuscript would benefit from a more explicit discussion of how the dynamic electricity grid mix varies seasonally across the study geography, as this is central to the framework's claimed advantage over static approaches. Furthermore, the analysis is limited to a single year; multi-year profiles (four or more years) would substantially strengthen the conclusions regarding seasonal patterns and decarbonisation trajectories.

No sensitivity analysis is presented. Given the reliance on normalised non-metered inputs (waste, staff travel), a formal uncertainty or sensitivity assessment is essential to substantiate the robustness of the reported results.

The full life cycle inventory is not made available, limiting reproducibility. Authors should provide the inventory data as supplementary material in accordance with open science principles.

- Minor Comments

Total emissions figures (39,000,000–49,000,000 kg CO_2e) should be expressed in kt CO_2e for readability and consistency with reporting conventions under the GHG Protocol and ISO 14064.

Figure 6 presents per-visit footprint data for the eighth day of each month only. The rationale for this choice is not explained; monthly averages would be more representative and should be justified or replaced accordingly. Similarly, the choice of a 4-hour functional unit warrants explicit justification in the Methods section.

Results would benefit from greater quantitative comparison across emission categories, enabling the reader to assess relative contributions more precisely. The current presentation is predominantly descriptive.

The validation of SPA-DEC on a single facility — albeit a large and well-documented one — limits the generalisability of the framework. The inclusion of additional case studies, whether across distinct spa typologies or contrasting geographies with different grid mixes, climatic profiles, and operational scales, would substantially strengthen the claim that SPA-DEC is scalable and transferable. In its current form, the framework's broader applicability remains an assertion rather than a demonstrated outcome.

The repeated and prominent framing of Therme Bucharest throughout the manuscript risks lending a promotional tone to what should remain a methodologically focused scientific contribution. The case study should be introduced once and thereafter treated as an illustrative vehicle for the framework, rather than a subject of sustained attention.

- Overall Assessment

The work addresses a genuine gap in sectoral LCA coverage and the core allocation methodology is novel within its context. However, the scope misclassifications, absence of sensitivity analysis, limited temporal scope, and underdeveloped methodological narrative currently constrain the manuscript's contribution. Revision addressing these points would substantially improve its suitability for publication.

Reviewer #2

(Remarks to the Author)
The authors provide a timely and essential contribution to an under-researched sector. The SPA-DEC framework is commendable for its scalability and its ability to translate complex Scope 1, 2, and 3 emissions into a "per-visitor" metric. By normalizing non-metered sources like staff travel and waste alongside real-time energy data, the tool offers a transparent benchmarking system that is highly supportive of industry-wide decarbonization.

While the validation at Therme Bucharest provides a strong proof-of-concept, the transition from a footprinting tool to rigorous science requires a deeper dive into the data already captured. How do emissions profiles shift during peak social hours versus maintenance cycles, etc.? Providing this kind of granular data analysis (hourly, daily, and annual patterns) would transform the paper from a case study of one facility into a data set for the wellness industry.

Because this study is validated on a single, unique facility (Therme Bucharest), its scientific credibility relies on the depth of the data rather than its breadth. By analyzing these high-resolution temporal patterns, the authors could reveal how the Climate Change KPIs on Scope 1, 2 and 3 fluctuates in relation to other critical reporting points like peak water demand or seasonal waste spikes. This would allow for a more transparent sustainability report that recognizes the energy-water-waste nexus rather than treating carbon as a standalone variable.The scenario modeling (geothermal and PV) provides appropriate best practices for the industry. However, to elevate this framework from a reporting tool to a strategic transition roadmap, the framework should help facility managers navigate the proactive transition.

Reviewer #3

(Remarks to the Author)
The authors address the lack of sector-wide carbon accounting methods for the spa and wellness industry, which is known to have high energy consumption but limited sustainability measurement frameworks. To fill this gap, they develop and demonstrate a sector-specific emissions calculator. Further, they present detailed the results for Therme Bucharest, one of the worlds largest spa and wellness centers, which provides insights on the main sources for GHG emissions for spa and wellness centers. While I believe this study presents useful information, I suggest major revisions prior to publication to improve the relevance of the study.
1. Consider broadening the scope and usability of the tool: the authors discuss how the tool can be used across spa centers, but I think it would be interesting if there was some applicability to any other service-based infrastructure such as other leisure facilities, theme parks, etc. Therefore, the authors should clarify the broader scientific contribution of the work, and make modifications to the tool, if possible, to enable wider usage.
2. Need for uncertainty analysis: Given that a portion of impacts (14%) come from the consumption of products, and that this data appears to come from a variety of sources, an uncertainty analysis of these impacts is necessary. As a result, the authors could incorporate discussion of uncertainty in input data, error propagation through the allocation model, and confidence intervals for per-visitor emissions.
3. Additional environmental impact discussions: The authors only report GHG emissions, with the reasoning that this is the most common metric reported in LCA. While this is true, I believe presenting results for other impact categories such as water use, particulate matter emissions, eutrophication impacts etc., would be very valuable. For example, I would expect spas to be water-intensive due to the large heated pools and thermal baths, continuous filtration and water replacement, intensive laundry operations, and extensive cleaning and chemical treatment. Based on the description of the methods, reporting these additional impacts should be straightforward, assuming that all of the inventories were built out in an LCA database such as SimaPro.

Reviewer #4

(Remarks to the Author)
This paper presents a carbon-footprinting approach intended for use by spa facilities, together with a case study of the

Thermae Bucharest spa. The approach uses partial dynamic allocation of carbon emissions to specific visits (based on energy and water use metered to be consumed within hourly periods matching the visit) together with annual allocation of overheads and closed-hours emissions to all visitors.

I am unsure where the authors intend the main significance of the paper to lie. The approach seems to be an interesting application of attributional carbon accounting to a particular type of organisation, which is nontrivial due to the different types of energy and purchases involved, but it's not clear in the way it's presented what is novel about this. If the intended contribution is about establishing the methodology that spas should use, there should be greater clarity on the need for this (why are spas different from other patterns that could be copied?), what they key choices are, and why the proposed method is better than alternative approaches that could have been proposed (see e.g. the questions listed below). If the intended contribution is about the findings from the case study, there should be greater clarity on what the implications of this are and how they can be used to make decisions that were not possible before.

The details of the methods can be made more consistent and explicit. Some of the case study results would benefit from more explanation. As per LCA in general, uncertainty and sensitivity to assumptions should be discussed.

While (with some corrections/clarifications, as below) the paper clearly represents a solid piece of publishable work, I am not sure that it meets the criteria for publication in Communications Earth & Environment, namely that the conclusions are novel (as above, this should be made more explicit what they are and why they are novel), and that "the underlying data are generated and processed at a state-of-the-art level, including tracking of uncertainties" (some level of discussion of uncertainties and sensitivity is essential in this type of analysis, but currently only mentioned in passing).

Specific comments:

Line 40: the lack of estimates of *global* GHG footprints of the spa industry doesn't seem relevant to this study

Line 111: this cited study seems to be about cities, and doesn't obviously demonstrate that scope 3 emissions often represent the largest portion of an organisation's footprint?

Line 132: should explain how the tool is "aligned with emerging carbon offsetting frameworks"

Results:

Figure 1: What does "on-site geothermal" represent that leads to it being the 6th largest category of annual emissions? This seems surprising and worth explaining.

Table 1: Why is geothermal four-hourly rather than hourly like the others? How is this accounted for in the methods, which only mention hourly energy consumption?

Figure 3: What do the "geothermal" and "PV" emissions represent here? The methods do not explain how they are calculated.

Line 189: Presumably the time period over which this is done is an important modelling choice, but it's not discussed?

Line 197: The justification for the open/closed hours method is also not clear -- how do you know that you shouldn't assume the closed-hours energy consumption represents a baseline with the marginal per-visitor footprint being the excess above this, for example, rather than 100% of the open-hours energy consumption?

Line 215: Why is a 4-hour visit used? Are the results sensitive to this?

Line 229: If individual days are so variable in visitor numbers, why is it relevant to present results from arbitrary individual days? The suggestion is that we should infer seasonal patterns from Figure 6, but this is already shown more clearly in the later figures? Why are October and December so high?

Discussion:

Line 260: Isn't the 32% figure based simply on the total annual energy consumption and emissions of the different energy sources? Can you elaborate how the dynamic allocation enables this analysis?

Line 278: Currently, the results do not prove that the approach can "accurately" do this

Line 280: "very straightforward" -- is it? If less real-time occupancy or metered data was available, wouldn't that make it difficult to apply your dynamic allocation method?

Line 296: Rationale for why closed-hour overheads should be allocated per-visitor (not accounting for their duration of visit) isn't clear

Line 353: These operational examples all seem to be good examples where having hourly energy consumption/emissions data is indeed useful -- but it's not obvious that the dynamic allocation per-visitor helps? e.g. reducing peak grid reliance will

help reduce emissions regardless of how many visitors are present at the time. The significance of the allocated footprints for different decisions is not fully clear...

Line 391: ... this scenario is switching from retrospective accounting of per-visit footprints, to trying to support a decision (presumably) about when or whether a visitor will come to the spa. It's not obvious that the metric being reported is appropriate for both; if you are recommending its use for this decision can you elaborate on this?

Methods:

Line 439: the goal and scope should also discuss the goal with respect to the decisions that this analysis aims to support (e.g. in relation to the question about different decisions above)

Line 449: "gate-to-gate" -- isn't this confusing as it's not the same sense as in "cradle-to-gate"?

Line 450: Elsewhere is it not linked to visit duration?

Line 499 and following: The equations are not consistent - the later ones seem to overlap with the first ones but using different notation. Open/closed hours are not reflected in Equation 2.

Line 514: This should be elaborated if included.

Figure 8: How are "per unique visitor" outputs obtained separately from "normalisation and dynamic emissions allocation"? What does "normalisation" mean here -- it doesn't seem to be referring to LCIA normalisation?

Line 527: This is inconsistent with equation 2

Line 538: Wrong H

Line 540: Unclear what "the visitors associated with day d" means - it sounds like it should be the calendar day but then you say "added to the next N_d visits". Does it make any difference?

Line 542: How are the outlier thresholds set? Why is it right to reallocate 100% of outliers?

Line 584: "This approach ensures simplicity while methodological rigour," - unclear what this is referring to

Line 611-645: This all seems like generic Scope 3 GHG accounting; is there intended to be anything specific to spas here?

Version 1:

Reviewer comments:

Reviewer #1

(Remarks to the Author)
The authors have addressed all major concerns raised in the first review in a thorough and convincing manner. The revised manuscript is substantially stronger.

The Monte Carlo uncertainty analysis is a welcome addition, the scope classifications have been corrected throughout, and the anonymisation of the primary facility resolves the promotional tone flagged previously. The shift to monthly averages and the more explicit methodological framing also improve the overall clarity of the work.

The second case study demonstrates transferability effectively, though it is worth briefly acknowledging that a university wellness facility operates under a fundamentally different business model to a commercial spa, which may carry implications.

On reproducibility, the commitment to release the full inventory and codebase via GitHub upon acceptance is appreciated. Making anonymized category-level totals available at the review stage would have been preferable, but this does not affect the overall assessment.

I recommend acceptance.

Reviewer #3

(Remarks to the Author)
Thank you for the revised manuscript and for the detailed point-by-point responses. I appreciate the authors’ efforts in addressing the reviewer comments. In general, I feel that my previous concerns have been satisfactorily addressed, and the revisions have improved the clarity and overall quality of the manuscript. Based on the changes made, I have no further major comments and would support the manuscript for publication in its current form.

Reviewer #4

(Remarks to the Author)
Thank you to the authors for making revisions which substantially address the reviewers' comments about clarity of the methodology and application of the presented tool, including the uncertainty analysis which is an important addition to the proposed approach.

The authors clarified that their focus is the paper's methodological contribution, rather than the case studies. With this, I would expect that the method be situated explicitly against the literature/state-of-the-art approaches, and to have specific conclusions about the merits of the proposed method, which I still find is not the case. By this I mean, for example:

(a) alternative methods -- these are added in a new paragraph in general terms but with no references or specifics. There are relevant approaches/examples, perhaps outside of the very narrow spa application, of per-visitor carbon accounting methods e.g. for hotels -- if this is the methodological problem being solved then it deserves explicitness and references. Alternatively if, say, it's the "LCA-aligned emissions-factor selection" suitable for spas which was previously missing, what data is currently available to spa analysts and how should they know that your figures are better?

(b) findings -- if proposing a new method for calculating per-visitor footprints, one might expect comparisons to alternative methods that could have been used -- either to show an improvement over simpler methods, or to show validation against a more sophisticated method. Currently the conclusions focus on the case study specific footprints, with general comments about the need for specific accounting.

Separately, two specific points:

While it's good to see uncertainty analysis, there is no explanation for the uncertainty values chosen -- this could refer to a standardised method like the pedigree matrix, reference other sources for why these are appropriate uncertainty levels, or give an explanation of how they were estimated.

Thank for you for clarifying about PV and geothermal representing embodied emissions amortised over expected system lifetime; I would suggest checking the GHG Protocol definitions for this as I believe the scope 2 guidance includes only emissions at the point of generation, which is in tension with the LCA perspective.

Open Access This Peer Review File is licensed under a Creative Commons Attribution 4.0 International License, which permits use, sharing, adaptation, distribution and reproduction in any medium or format, as long as you give appropriate credit to the original author(s) and the source, provide a link to the Creative Commons license, and indicate if changes were made.
In cases where reviewers are anonymous, credit should be given to 'Anonymous Referee' and the source.
The images or other third party material in this Peer Review File are included in the article’s Creative Commons license, unless indicated otherwise in a credit line to the material. If material is not included in the article’s Creative Commons

license and your intended use is not permitted by statutory regulation or exceeds the permitted use, you will need to obtain permission directly from the copyright holder.
To view a copy of this license, visit https://creativecommons.org/licenses/by/4.0/

SPA-DEC Responses to Reviewer Comments

<u>Reviewer #1 (Remarks to the Author):</u>

This manuscript introduces SPA-DEC, a dynamic emissions calculator applied to spa and wellness facilities, and represents one of the first sector-specific operational LCA frameworks for this industry. The allocation approach — dividing emissions between visitor-hours and closed-hour maintenance — is methodologically intuitive and a genuine contribution to the field. The case study is well-executed, and the writing is clear throughout. However, several concerns should be addressed before the work can be considered for publication.

We thank the reviewer for their comments and welcome their positive assessment of the allocation methodology and case study. We have addressed each of the concerns raised below in full.

The manuscript contains notable inconsistencies in greenhouse gas scope classification that undermine the methodological rigour of the framework. Fugitive emissions are misclassified: these constitute Scope 1 emissions, not Scope 2. On-site photovoltaic systems, where equipment is purchased rather than leased, are more appropriately categorised under Scope 3 (capital goods) rather than Scope 2. Similarly, company transport is consistent with Scope 3 commuting. These misclassifications recur in the discussion of Figure 2 and should be corrected systematically throughout.

The scope misclassifications have been corrected systematically throughout the revised manuscript, including the discussion of Figure 2 (now Figure 1) and Table 1. Fugitive refrigerant emissions are now correctly classified as Scope 1, reflecting direct releases from owned or controlled equipment. On-site photovoltaic infrastructure, where equipment is purchased rather than leased, has been reclassified as Scope 3 (capital goods), reflecting its embodied upstream manufacturing emissions.

In addition to on-site photovoltaic, we have reclassified the on-site geothermal impact as Scope 3. This is because these emissions relate to the upstream embodied emissions of the energy infrastructure. The Large Wellness Facility uses a binary/closed-loop heat extraction. The geothermal fluid circulates underground, and heat is transferred via a heat exchanger, with no direct venting of geothermal gases. The electricity required for the pump is counted within the electricity consumption (purchased electricity and photovoltaic).

Regarding company transport: we respectfully maintain its classification as Scope 1. Under the GHG Protocol Corporate Standard, fuel combustion from spa-owned

vehicles used in the delivery of visitor services constitutes a direct emission from a controlled source and is therefore Scope 1. Staff commuting is correctly classified as Scope 3 and is reported separately throughout.

The novelty of SPA-DEC lies primarily in its allocation criteria and dynamic temporal resolution; however, this methodological contribution is underexplored relative to the case study results. The manuscript would benefit from a more explicit discussion of how the dynamic electricity grid mix varies seasonally across the study geography, as this is central to the framework's claimed advantage over static approaches.

The methodological contribution of SPA-DEC has been substantially strengthened in the revised manuscript. The Introduction now includes a dedicated paragraph explicitly positioning SPA-DEC's allocation approach relative to two alternative methodologies: the static annual ratio and dynamic hourly allocation without open/closed hour distinction. Then we demonstrate why neither adequately resolves the challenges of visitor-level attribution in spa facilities.

Regarding seasonal grid mix variation, this is now addressed explicitly in the Results. Using hourly grid generation data sourced from the national transmission system operator, the purchased electricity mix was resolved at hourly resolution for each month of 2024. By linking this hourly grid composition data to hourly facility electricity consumption, SPA-DEC applies a temporally resolved emission factor.

Furthermore, the analysis is limited to a single year; multi-year profiles (four or more years) would substantially strengthen the conclusions regarding seasonal patterns and decarbonisation trajectories.

The analysis is validated across two contrasting facilities for the full 2024 calendar year. The inclusion of a second, structurally distinct facility strengthens conclusions regarding seasonal patterns and demonstrates that SPA-DEC's capability is not confined to a single operational context. We acknowledge that multi-year profiles would further strengthen decarbonisation trajectory conclusions, and this is identified as a priority for future work in the revised Limitations section.

No sensitivity analysis is presented. Given the reliance on normalised non-metered inputs (waste, staff travel), a formal uncertainty or sensitivity assessment is essential to substantiate the robustness of the reported results.

A full Monte Carlo uncertainty and sensitivity analysis has been added to the Results. Uncertain inputs are represented as lognormal multipliers with coefficients of variation calibrated by data quality tier, with higher uncertainty assigned to non-metered categories including waste, staff commuting, and fugitive losses. The

analysis yields 95% uncertainty intervals and Spearman rank-correlation tornado diagrams for both facilities, identifying the dominant uncertainty drivers at each site and confirming the robustness of the reported per-visitor footprints.

The full life cycle inventory is not made available, limiting reproducibility. Authors should provide the inventory data as supplementary material in accordance with open science principles.

The full results and figure data underlying the case studies will be provided upon acceptance, enabling direct verification of the reported outputs. The supplementary includes the full methodological inventory: emission factor sources for consumables, treatments, and cleaning chemicals (Table S4); waste treatment emission factors (Table S5); the structure of metered and non-metered input categories (Tables S1–S3); and full case study setup descriptions for both facilities. The underlying facility operational activity data are commercially sensitive and cannot be disclosed in full; however, a more comprehensive inventory containing facility-level emission factors and category-level totals will be made available in full upon acceptance to support open science and reproducibility. The complete SPA-DEC codebase and spreadsheet template will be made publicly available via GitHub upon acceptance.

Total emissions figures (39,000,000–49,000,000 kg CO□e) should be expressed in kt CO□e for readability and consistency with reporting conventions under the GHG Protocol and ISO 14064.

The total emissions figures in the Introduction have been converted from kg to kt CO_2e for readability and consistency with GHG Protocol and ISO 14064 reporting conventions.

Figure 6 presents per-visit footprint data for the eighth day of each month only. The rationale for this choice is not explained; monthly averages would be more representative and should be justified or replaced accordingly.

Figure 6 (now Figure 5) has been revised to present monthly averages across all days of each calendar month for both facilities, providing a more representative picture of seasonal variation in per-visit footprints.

Similarly, the choice of a 4-hour functional unit warrants explicit justification in the Methods section.

We note that the 4-hour and 1.5-hour durations are not the functional unit of the study, which is defined as one visit from entry to exit regardless of duration. These

durations are illustrative standard values reflecting the typical commercial entry period at each facility, used to enable consistent temporal and cross-facility comparison in the figures. A clarifying statement to this effect has been added to the Results. SPA-DEC is not constrained to any fixed duration. The dynamic allocation framework produces a per-visitor footprint for any stay length.

Results would benefit from greater quantitative comparison across emission categories, enabling the reader to assess relative contributions more precisely. The current presentation is predominantly descriptive.

The revised manuscript presents category-level contributions across multiple figures. Figure 1 provides scope-level percentage breakdowns for both facilities. Figure 5 decomposes the per-visit footprint by individual emission source across all months, enabling direct quantitative comparison of category contributions and their seasonal variation. Figure 7 provides Spearman rank-correlation coefficients quantifying the relative influence of each input on output uncertainty. Underlying data will be made publicly available, enabling readers to interrogate category-level contributions in full.

The validation of SPA-DEC on a single facility — albeit a large and well-documented one — limits the generalisability of the framework. The inclusion of additional case studies, whether across distinct spa typologies or contrasting geographies with different grid mixes, climatic profiles, and operational scales, would substantially strengthen the claim that SPA-DEC is scalable and transferable. In its current form, the framework's broader applicability remains an assertion rather than a demonstrated outcome.

The revised manuscript includes a second case study: a university-based swimming pool and wellness zone. This provides contrasting validation context across facility scale, operational complexity, energy mix, and geography. The two facilities differ substantially. The Large Wellness Facility is a large thermal resort with geothermal and photovoltaic integration, a diversified three-scope emission profile, and over 1.6 million annual visitors; the Small Wellness Facility is a compact, two-source facility with a visitor base an order of magnitude smaller. SPA-DEC's core allocation logic is applied without modification across both, demonstrating transferability across contrasting spa typologies and data maturities. The claim of scalability is therefore a demonstrated outcome of the revised manuscript rather than an assertion.

The repeated and prominent framing of Therme Bucharest throughout the manuscript risks lending a promotional tone to what should remain a methodologically focused scientific contribution. The case study should be

introduced once and thereafter treated as an illustrative vehicle for the framework, rather than a subject of sustained attention.

The primary case study facility has been anonymised throughout the revised manuscript and is referred to consistently as "Large Wellness Facility." The facility is introduced once and thereafter treated as an illustrative vehicle for the framework, with the methodological contribution remaining the primary focus throughout.

The work addresses a genuine gap in sectoral LCA coverage and the core allocation methodology is novel within its context. However, the scope misclassifications, absence of sensitivity analysis, limited temporal scope, and underdeveloped methodological narrative currently constrain the manuscript's contribution. Revision addressing these points would substantially improve its suitability for publication.

We thank Reviewer 1 for the constructive and thorough assessment. Each of the concerns (scope misclassifications, absence of sensitivity analysis, limited temporal scope, and underdeveloped methodological narrative) have been addressed in the revised manuscript, as detailed above. We are confident that the revisions substantially strengthen the contribution and its suitability for publication.

Reviewer #2 (Remarks to the Author):

The authors provide a timely and essential contribution to an under-researched sector. The SPA-DEC framework is commendable for its scalability and its ability to translate complex Scope 1, 2, and 3 emissions into a "per-visitor" metric. By normalizing non-metered sources like staff travel and waste alongside real-time energy data, the tool offers a transparent benchmarking system that is highly supportive of industry-wide decarbonization.

We thank the reviewer for their comments and welcome their positive assessment of the scalable and transparent methodology. We have addressed each of the concerns raised below in full.

While the validation at Therme Bucharest provides a strong proof-of-concept, the transition from a footprinting tool to rigorous science requires a deeper dive into the data already captured. How do emissions profiles shift during peak social hours versus maintenance cycles, etc.? Providing this kind of granular data analysis (hourly, daily, and annual patterns) would transform the paper from a case study of one facility into a data set for the wellness industry.

The revised manuscript provides granular temporal analysis across multiple resolutions. Figure 2 presents hourly per-visitor metered consumption emissions across the full year, Figure 3 disaggregates closed-hour maintenance emissions by

source, Figure 5 provides monthly per-visit footprint decompositions by emission category, and Figure 6 presents average daily and monthly patterns by weekday and season. Together these figures distinguish peak operational hours from maintenance cycles and reveal how emissions profiles shift across daily, weekly, and seasonal timescales. The addition of a second case study further extends the dataset beyond a single facility, providing a comparative reference point for the wellness industry.

Because this study is validated on a single, unique facility (Therme Bucharest), its scientific credibility relies on the depth of the data rather than its breadth. By analyzing these high-resolution temporal patterns, the authors could reveal how the Climate Change KPIs on Scope 1, 2 and 3 fluctuates in relation to other critical reporting points like peak water demand or seasonal waste spikes.

The revised manuscript now validates SPA-DEC across two contrasting facilities, addressing the concern about single-facility credibility.

This would allow for a more transparent sustainability report that recognizes the energy-water-waste nexus rather than treating carbon as a standalone variable.

We thank the reviewer for this contribution. We agree this would be a valuable extension of the framework. SPA-DEC's primary scope is GHG accounting aligned with organisational reporting requirements under the GHG Protocol and ISO 14064. Extending to a full nexus analysis incorporating water demand and waste intensity alongside carbon would require additional inventory development and impact characterisation beyond the current framework. Multi-impact assessment is now acknowledged explicitly in the revised Limitations section as a priority for future work.

The scenario modeling (geothermal and PV) provides appropriate best practices for the industry. However, to elevate this framework from a reporting tool to a strategic transition roadmap, the framework should help facility managers navigate the proactive transition.

The positive assessment of the scenario modelling is appreciated. The revised Implementation section now explicitly frames SPA-DEC as a strategic planning tool alongside its reporting function.

Reviewer #3 (Remarks to the Author):

The authors address the lack of sector-wide carbon accounting methods for the spa and wellness industry, which is known to have high energy consumption but limited sustainability measurement frameworks. To fill this gap, they develop and

demonstrate a sector-specific emissions calculator. Further, they present detailed the results for Therme Bucharest, one of the worlds largest spa and wellness centers, which provides insights on the main sources for GHG emissions for spa and wellness centers. While I believe this study presents useful information, I suggest major revisions prior to publication to improve the relevance of the study.

We thank the reviewer for their comments and welcome their positive assessment of the case study. We have addressed each of the concerns raised below in full.

1. Consider broadening the scope and usability of the tool: the authors discuss how the tool can be used across spa centers, but I think it would be interesting if there was some applicability to any other service-based infrastructure such as other leisure facilities, theme parks, etc. Therefore, the authors should clarify the broader scientific contribution of the work, and make modifications to the tool, if possible, to enable wider usage.

The broader applicability of SPA-DEC's allocation framework is now explicitly addressed in the revised manuscript. The Strengths section discusses the transferability of the core allocation logic to a wider class of visitor-economy service facilities, including leisure centres, aquatic facilities, and other service-based infrastructure where operational emissions must be attributed to individual users. This is demonstrated in practice through the inclusion of a second case study, which applies SPA-DEC without modification to the core framework across a fundamentally different operational context. Full adaptation to other sectors would require sector-specific inventory development, but the methodological framework does not present barriers to extension and is designed with this modularity in mind.

2. Need for uncertainty analysis: Given that a portion of impacts (14%) come from the consumption of products, and that this data appears to come from a variety of sources, an uncertainty analysis of these impacts is necessary. As a result, the authors could incorporate discussion of uncertainty in input data, error propagation through the allocation model, and confidence intervals for per-visitor emissions.

A full Monte Carlo uncertainty and sensitivity analysis has been added to the Results (Figure 7). The analysis propagates uncertainty through the allocation model across all input categories, with coefficients of variation calibrated by data quality tier, including higher uncertainty assigned to product consumption and other non-metered categories. The results include 95% confidence intervals for per-visitor emissions and Spearman rank-correlation sensitivity rankings for both facilities, directly identifying the contribution of each input category to total output variance.

3. Additional environmental impact discussions: The authors only report GHG emissions, with the reasoning that this is the most common metric reported in LCA. While this is true, I believe presenting results for other impact categories such as water use, particulate matter emissions, eutrophication impacts etc., would be very valuable. For example, I would expect spas to be water-intensive due to the large heated pools and thermal baths, continuous filtration and water replacement, intensive laundry operations, and extensive cleaning and chemical treatment. Based on the description of the methods, reporting these additional impacts should be straightforward, assuming that all of the inventories were built out in an LCA database such as SimaPro.

We thank the reviewer for this contribution. We agree that multi-impact assessment would be valuable for water-intensive facilities. However, SPA-DEC is designed as an accessible, sector-specific emissions calculator intended for use by facility operators or visitors without specialist LCA software. Conducting the case study analysis within SimaPro or equivalent platforms would undermine this design principle and defeat the purpose of developing a standalone tool. Emission factors for relevant inventory items were derived from Ecoinvent datasets characterised using the IPCC GWP100 method and implemented within SPA-DEC. Extending the framework to additional impact categories such as water use and eutrophication is a clear priority for future development and is now acknowledged in the revised Limitations section.

Reviewer #4 (Remarks to the Author):

This paper presents a carbon-footprinting approach intended for use by spa facilities, together with a case study of the Thermae Bucharest spa. The approach uses partial dynamic allocation of carbon emissions to specific visits (based on energy and water use metered to be consumed within hourly periods matching the visit) together with annual allocation of overheads and closed-hours emissions to all visitors.

We thank the reviewer for their comments. We have addressed each of the concerns raised below in full.

I am unsure where the authors intend the main significance of the paper to lie. The approach seems to be an interesting application of attributional carbon accounting to a particular type of organisation, which is nontrivial due to the different types of energy and purchases involved, but it's not clear in the way it's presented what is novel about this. If the intended contribution is about establishing the methodology that spas should use, there should be greater clarity on the need for this (why are spas different from other patterns that could be copied?), what they key choices are, and why the proposed method is better than alternative approaches that could have been proposed (see e.g. the questions listed below). If the intended contribution is

about the findings from the case study, there should be greater clarity on what the implications of this are and how they can be used to make decisions that were not possible before.

The revised manuscript addresses this directly on two fronts. First, the Introduction now includes a dedicated paragraph explicitly positioning SPA-DEC's methodological contribution relative to two alternative approaches: the static annual ratio and dynamic hourly allocation without open/closed hour distinction. We explain the limitations of each and demonstrate why SPA-DEC's approach is methodologically superior. This clarifies both why spas require a sector-specific tool and why the proposed allocation logic is preferable to simpler alternatives. Second, the Goal and Scope section now states four explicit intended applications of the analysis in accordance with ISO 14040, establishing that the primary contribution is methodological: the framework itself, rather than the findings of any individual case study. The case study results are presented as a demonstration of what the framework makes possible: temporal hotspot identification, renewable scenario evaluation, and visitor-level attribution – none of which are achievable with conventional annual inventory approaches.

The details of the methods can be made more consistent and explicit. Some of the case study results would benefit from more explanation. As per LCA in general, uncertainty and sensitivity to assumptions should be discussed.

The revised manuscript addresses each of these points in full. Methodological consistency and explicitness have been improved throughout the Methods section, case study results are accompanied by expanded quantitative and interpretive discussion, and a full Monte Carlo uncertainty and sensitivity analysis has been added to the Results. Each specific concern is addressed in detail below.

While (with some corrections/clarifications, as below) the paper clearly represents a solid piece of publishable work, I am not sure that it meets the criteria for publication in Communications Earth & Environment, namely that the conclusions are novel (as above, this should be made more explicit what they are and why they are novel), and that "the underlying data are generated and processed at a state-of-the-art level, including tracking of uncertainties" (some level of discussion of uncertainties and sensitivity is essential in this type of analysis, but currently only mentioned in passing).

Both criteria are addressed in the revised manuscript. The novelty of SPA-DEC's methodological contribution is now explicitly established in the Introduction through a direct comparison with alternative approaches, and the Goal and Scope section frames the four intended applications of the analysis in accordance with ISO 14040. A full Monte Carlo uncertainty and sensitivity analysis has been added to the

Results, with uncertainty propagated through the allocation model and reported at state-of-the-art level across both case study facilities.

Line 40: the lack of estimates of *global* GHG footprints of the spa industry doesn't seem relevant to this study

The reference to the absence of global GHG footprint estimates for the spa industry has been removed from the Introduction. The revised text instead focuses directly on the lack of a standardised per-visit emission metric and the absence of consistent boundaries and allocation methods, which is directly relevant to the contribution of this study.

Line 111: this cited study seems to be about cities, and doesn't obviously demonstrate that scope 3 emissions often represent the largest portion of an organisation's footprint?

Reference 34 has been replaced with a more directly applicable source. The revised manuscript cites Ducoulombier (2021), which addresses the importance and scale of Scope 3 emissions specifically in the context of organisational reporting, directly supporting the claim made at this point in the Introduction.

Line 132: should explain how the tool is "aligned with emerging carbon offsetting frameworks"

The revised manuscript now explains the basis of alignment: the mechanism requires verified, visit-level carbon attribution, which is precisely what SPA-DEC's dynamic per-visitor allocation produces.

Figure 1: What does "on-site geothermal" represent that leads to it being the 6th largest category of annual emissions? This seems surprising and worth explaining.

The original Figure 1 has been removed in the revised manuscript; the explanation of the geothermal contribution is now incorporated into the Scope Breakdown section. On-site geothermal appears as the sixth largest emission category despite carrying one of the lowest life cycle emission factors of any energy source. This figure represented the embodied emissions of the geothermal infrastructure amortised over the system lifetime, rather than direct release of geothermal gases. Its material contribution to the annual total reflects the scale of geothermal heat consumption: as the facility's primary thermal energy source, the volume of heat extracted is very large, and even at low emission intensity, the absolute annual total is significant. An explanatory note to this effect has been added to the Results Section 2.2.

Table 1: Why is geothermal four-hourly rather than hourly like the others? How is this accounted for in the methods, which only mention hourly energy consumption?

Table 1 was originally specific to the Large Wellness Facility but has been updated to reflect typical SPA-DEC inputs. This includes hourly resolution for geothermal, consistent with the other metered sources. In the Large Wellness Facility case study, geothermal consumption was recorded at four-hourly intervals. SPA-DEC handles sub-hourly data gaps through uniform temporal disaggregation: four-hourly totals are divided equally across the constituent hours, preserving the overall energy balance while aligning with the hourly allocation framework. This is consistent with SPA-DEC's broader approach to data maturity. The framework is designed to accommodate varying input resolutions without compromising the integrity of the per-visitor allocation, and higher-resolution metering can be substituted directly where available. A clarifying note on this approach has been added to the Temporal Allocation section of the Methods, which also explains where disaggregation is and is not appropriate.

Figure 3: What do the "geothermal" and "PV" emissions represent here? The methods do not explain how they are calculated.

The revised manuscript now explains what geothermal and PV emissions represent in the Results Section 2.2. Both are lifecycle emission factors sourced from Ecoinvent, representing the embodied upstream emissions of the on-site infrastructure, amortised over the system lifetime and expressed per kWh of energy produced or consumed. Neither represents direct combustion emissions.

Line 189: Presumably the time period over which this is done is an important modelling choice, but it's not discussed?

The choice of a one-year aggregation period for non-metered inputs is now explicitly justified in the revised Methods. Annual aggregation aligns with standard GHG reporting cycles under the GHG Protocol and ISO 14064, ensures the normalisation denominator captures the full seasonal range of visitor throughput, and smooths short-term procurement variation that would otherwise introduce noise into per-visitor estimates.

Line 197: The justification for the open/closed hours method is also not clear -- how do you know that you shouldn't assume the closed-hours energy consumption represents a baseline with the marginal per-visitor footprint being the excess above this, for example, rather than 100% of the open-hours energy consumption?

An allocation rationale paragraph has been added to the Methods following the temporal allocation steps. Regarding open-hour allocation: 100% of open-hour energy is attributed to visitors because all energy consumed during opening hours is directly in service of delivering the visitor experience. Regarding closed-hour allocation: SPA-DEC adopts a full-cost approach consistent with the ISO 14040/44 principle that all inputs within the defined system boundary are attributed to the functional unit. The marginal approach of leaving closed-hour baseline energy unattributed would systematically understate the total service footprint and undermine cross-facility comparability. Closed-hour overheads are additionally allocated equally per visitor rather than weighted by duration, as these costs are fixed and independent of stay length.

Line 215: Why is a 4-hour visit used? Are the results sensitive to this?

We note that the 4-hour and 1.5-hour durations are not the functional unit of the study, which is defined as one visit from entry to exit regardless of duration. These durations are illustrative standard values reflecting the typical commercial entry period at each facility, used to enable consistent temporal and cross-facility comparison in the figures. A clarifying statement to this effect has been added to the Results. SPA-DEC is not constrained to any fixed duration. The dynamic allocation framework produces a per-visitor footprint for any stay length. Regarding sensitivity: the per-visit footprint is partially sensitive to duration, as the open-hour metered component scales with stay length, while closed-hour overhead and non-metered components are fixed per visitor regardless of duration.

Line 229: If individual days are so variable in visitor numbers, why is it relevant to present results from arbitrary individual days? The suggestion is that we should infer seasonal patterns from Figure 6, but this is already shown more clearly in the later figures? Why are October and December so high?

Figure 6 (now Figure 5) in the revised manuscript now presents monthly averages across all days of each calendar month for both facilities, replacing the previous single-day snapshots. The elevated footprints in October and December are now explicitly explained in the Results: October reflects declining PV contribution combined with lower visitor numbers, which increases the overhead emissions allocated to each visit; December records the highest per-visit footprint due to peak natural gas demand when geothermal backup requirements are greatest and PV generation is negligible.

Line 260: Isn't the 32% figure based simply on the total annual energy consumption and emissions of the different energy sources? Can you elaborate how the dynamic allocation enables this analysis?

While the reduction could be derived from annual energy totals alone, the dynamic framework resolves this avoided-emissions benefit at daily-aggregated resolution across the year, as shown in Figure 4, revealing that the renewable contribution is most material during summer months when PV generation peaks for the Small Wellness Facility, and during the winter months when the thermal consumption requirement and therefore geothermal contribution is higher for the Large Wellness Facility.

Line 278: Currently, the results do not prove that the approach can "accurately" do this

The revised manuscript no longer contains this claim. The section in which it appeared has been substantially rewritten, and the language now describes what the results demonstrably show: that SPA-DEC's allocation logic is transferable across facility types and yields interpretable outputs at multiple temporal resolutions.

Line 280: "very straightforward" -- is it? If less real-time occupancy or metered data was available, wouldn't that make it difficult to apply your dynamic allocation method?

The revised manuscript no longer contains this claim. The section in which it appeared has been substantially rewritten, and the language now reflects the data requirements of the dynamic allocation method without overstating its ease of application.

Line 296: Rationale for why closed-hour overheads should be allocated per-visitor (not accounting for their duration of visit) isn't clear

This is addressed in the allocation rationale paragraph added to the Methods following the temporal allocation steps. Closed-hour overheads are fixed costs incurred independently of how long any individual visitor stays. Equal per-visitor allocation is therefore the methodologically appropriate choice: weighting by visit duration would imply that longer-staying visitors bear responsibility for a greater share of costs that do not, in practice, vary with their presence or duration.

Line 353: These operational examples all seem to be good examples where having hourly energy consumption/emissions data is indeed useful -- but it's not obvious that

the dynamic allocation per-visitor helps? e.g. reducing peak grid reliance will help reduce emissions regardless of how many visitors are present at the time. The significance of the allocated footprints for different decisions is not fully clear...

We thank the reviewer for this contribution. The significance of the per-visitor dimension for different decisions is addressed in the Dynamic Emissions Accounting section of the Interpretation. The reviewer is correct that hourly energy data supports operational decisions such as peak grid management independently of visitor allocation. The per-visitor dimension adds three things that aggregate facility data cannot provide: a functional unit that is directly comparable across facilities, time periods, and ticket types; visitor-level attribution required for carbon communication and offsetting mechanisms; and the demonstration that the footprint of an individual visit varies materially depending on when it occurs, which is actionable for demand management, visitor incentives, and transparency. These are decisions that require knowing not just total facility emissions but how they are distributed across visits.

Line 391: ... this scenario is switching from retrospective accounting of per-visit footprints, to trying to support a decision (presumably) about when or whether a visitor will come to the spa. It's not obvious that the metric being reported is appropriate for both; if you are recommending its use for this decision can you elaborate on this?

The pre-visit and post-visit uses of SPA-DEC apply the same functional unit and allocation logic — the only difference is that pre-visit estimates use forecast inputs rather than realised metered data. This consistency makes the metric appropriate for both purposes: a visitor comparing footprints for different visit windows is working from the same methodological basis as the post-visit receipt they would receive.

Line 439: the goal and scope should also discuss the goal with respect to the decisions that this analysis aims to support (e.g. in relation to the question about different decisions above)

The Goal and Scope section has been revised to explicitly state four intended applications of the analysis, covering facility-level benchmarking and CSR reporting, operational decarbonisation decision-making, visitor-level carbon communication and offsetting, and the development of a scalable sector-wide framework. These directly correspond to the decisions the analysis aims to support.

Line 449: "gate-to-gate" -- isn't this confusing as it's not the same sense as in "cradle-to-gate"?

The term "gate-to-gate" has been removed from the revised manuscript. The system boundary is described throughout as "from visitor entry to exit," avoiding any confusion with the cradle-to-gate convention used in product LCA.

Line 450: Elsewhere is it not linked to visit duration?

The functional unit is defined as one visit from entry to exit, independent of duration. A clarifying statement has been added to the Results noting that the 4-hour and 1.5-hour durations used in the figures are illustrative values reflecting typical visit lengths at each facility, used solely to enable consistent temporal and cross-facility comparison. SPA-DEC is not constrained to any fixed duration. The dynamic allocation framework produces a per-visitor footprint for any stay length.

Line 499 and following: The equations are not consistent - the later ones seem to overlap with the first ones but using different notation. Open/closed hours are not reflected in Equation 2.

Equation 2 has been revised to explicitly reflect the open/closed hour distinction, and the notation has been harmonised across the equation set and Steps 1–4 to ensure consistency throughout the Methods.

Line 514: This should be elaborated if included.

The sentence has been elaborated in the revised manuscript.

Figure 8: How are "per unique visitor" outputs obtained separately from "normalisation and dynamic emissions allocation"? What does "normalisation" mean here -- it doesn't seem to be referring to LCIA normalisation?

The flow diagram has been revised. "Normalisation and dynamic emissions allocation" has been replaced with "Dynamic visitor attribution and allocation" to avoid confusion with LCIA normalisation. The per-visitor output is now clearly positioned within the Outputs box as the direct result of the allocation step, clarifying that it is not obtained separately from the attribution process but is its primary output.

Line 527: This is inconsistent with equation 2

Equation 2 has been revised to explicitly reflect the open/closed hour distinction, and the notation has been harmonised across the equation set and Steps 1–4 to ensure consistency throughout the Methods.

Line 538: Wrong H

The incorrect H notation has been corrected.

Line 540: Unclear what "the visitors associated with day d" means - it sounds like it should be the calendar day but then you say "added to the next N_d visits". Does it make any difference?

Step 3 (now Step 4) has been written explicitly in the revised manuscript. Closed-hour emissions are aggregated and allocated equally across all unique visitors of the same calendar day. This provides a transparent and physically meaningful allocation rule that is consistent with the full-cost attribution principle applied throughout the framework.

Line 542: How are the outlier thresholds set? Why is it right to reallocate 100% of outliers?

Step 4 (now Step 3) has been clarified in the revised manuscript. Outlier hours are identified using a statistically grounded threshold: open-hour consumption values exceeding three standard deviations above the facility mean. This is consistent with standard anomaly detection practice in environmental monitoring. Excess emissions are capped and transferred to the closed-hour overhead pool rather than fully reclassified, preserving the total emissions balance.

Line 584: "This approach ensures simplicity while methodological rigour," - unclear what this is referring to

The sentence has been rewritten for clarity. The revised text explicitly identifies the approach of treating the per-visitor waste footprint as an annually normalised constant and explains that this balances methodological rigour with practical data requirements, accommodating facilities where waste data are available only at annual resolution.

Line 611-645: This all seems like generic Scope 3 GHG accounting; is there intended to be anything specific to spas here?

This reviewer is correct to point this out. The Business Operations section applies standard GHG Protocol Scope 3 calculation methods, which are necessarily generic as they follow internationally recognised guidance. The spa-specific content in this section lies in the relevance criteria applied to determine inclusion and exclusion.

Only emission sources that exist because of the facility's operation and visitor hosting are included, and Table S6 explicitly documents categories excluded as irrelevant to spa operation. The earlier consumables, treatments, and cleaning sections contain the spa-specific inventory content. The Business Operations section is intentionally positioned as a modular, standardised component that spa operators can implement without requiring bespoke methodological development for each category.

Responses to Reviewer Comments 2

COMMSSUSTAIN-26-0048A-Z

Reviewer #1 (Remarks to the Author):

The authors have addressed all major concerns raised in the first review in a thorough and convincing manner. The revised manuscript is substantially stronger.

The Monte Carlo uncertainty analysis is a welcome addition, the scope classifications have been corrected throughout, and the anonymisation of the primary facility resolves the promotional tone flagged previously. The shift to monthly averages and the more explicit methodological framing also improve the overall clarity of the work.

The second case study demonstrates transferability effectively, though it is worth briefly acknowledging that a university wellness facility operates under a fundamentally different business model to a commercial spa, which may carry implications.

On reproducibility, the commitment to release the full inventory and codebase via GitHub upon acceptance is appreciated. Making anonymized category-level totals available at the review stage would have been preferable, but this does not affect the overall assessment.

I recommend acceptance.

We thank the reviewer for their constructive comments from the current and previous review rounds and for recommending acceptance.

We agree with the reviewer's point that it's worth acknowledging the difference in business model between the case studies. For this reason, Section 2.2.1 has been renamed "Case Studies as Contrasting Applications". We have added a paragraph into this section explaining that the facilities are chosen to demonstrate the applicability of the methodology and that the resulting footprints reflect both operational efficiency and the breadth of amenities provided and should not be interpreted as a direct ranking of relative sustainability. The implication of this is that cross-facility benchmarking with SPA-DEC requires aligned system boundaries and comparable service definitions. This is also explained in Section 4.1 Goal and Scope.

The data underlying all figures and SPA-DEC output inventory are openly available on Figshare at https://doi.org/10.6084/m9.figshare.32825969, while the SPA-DEC repository, which includes the model library, the scripts that regenerate the figures from the aggregated outputs, a synthetic example dataset that allows the complete pipeline to be run without access to the proprietary facility data, a results

visualisation dashboard, and the per-facility configuration choices used for the two case studies is available on Zenodo at https://doi.org/10.5281/zenodo.21038901.

Reviewer #3 (Remarks to the Author):

Thank you for the revised manuscript and for the detailed point-by-point responses. I appreciate the authors' efforts in addressing the reviewer comments. In general, I feel that my previous concerns have been satisfactorily addressed, and the revisions have improved the clarity and overall quality of the manuscript. Based on the changes made, I have no further major comments and would support the manuscript for publication in its current form.

We thank the reviewer for their constructive comments across earlier review rounds and for supporting publication.

Reviewer #4 (Remarks to the Author):

Thank you to the authors for making revisions which substantially address the reviewers' comments about clarity of the methodology and application of the presented tool, including the uncertainty analysis which is an important addition to the proposed approach.

We thank the reviewer for recognising that the revisions have substantially addressed the previous concerns on methodological clarity and application. We address the two outstanding aspects of methodological positioning and the two specific points below.

The authors clarified that their focus is the paper's methodological contribution, rather than the case studies. With this, I would expect that the method be situated explicitly against the literature/state-of-the-art approaches, and to have specific conclusions about the merits of the proposed method, which I still find is not the case. By this I mean, for example:

(a) alternative methods -- these are added in a new paragraph in general terms but with no references or specifics. There are relevant approaches/examples, perhaps outside of the very narrow spa application, of per-visitor carbon accounting methods e.g. for hotels -- if this is the methodological problem being solved then it deserves explicitness and references. Alternatively if, say, it's the "LCA-aligned emissions-factor selection" suitable for spas which was previously missing, what data is currently available to spa analysts and how should they know that your figures are better?

We thank the reviewer for this comment. We have added a dedicated paragraph to the Introduction that situates SPA-DEC explicitly against the relevant state of the art.

The revised text identifies the established per-visitor and per-guest-night methods in adjacent visitor-economy sectors and cites them directly: the EU Product Environmental Footprint Category Rule for hotel accommodation services and hotel-specific carbon tools such as the Hotel Carbon Measurement Initiative, which apportion whole-property emissions to a guest-night using floor-area and occupancy-based allocation but rely on annual input data rather than temporally resolved emission factors. We distinguish these from the rigorous but facility- or scenario-level life-cycle studies in tourism and aquatic-centre contexts, which are too data-intensive for routine operational use. For spas specifically, we now state plainly that the only published footprints divide annual direct-energy consumption by user numbers, without time resolution, closed-hour treatment, or documented emission-factor provenance.

This directly addresses the reviewer's question of what is currently available to spa analysts and why SPA-DEC's figures are an improvement. The data presently available to the sector is limited to undocumented, energy-only annual averages; SPA-DEC's contribution is to provide a transparent, LCA-aligned, and temporally resolved alternative whose emission factors and allocation rules are documented and reproducible. The paragraph closes by stating the specific methodological position SPA-DEC occupies: the combination of visit-level temporal allocation, an explicit open/closed-hour distinction, and standardised, traceable LCA-aligned emission factors applied at hourly rather than annual resolution, with on-site renewable infrastructure represented through life-cycle factors rather than assumed to be zero.

(b) findings -- if proposing a new method for calculating per-visitor footprints, one might expect comparisons to alternative methods that could have been used -- either to show an improvement over simpler methods, or to show validation against a more sophisticated method. Currently the conclusions focus on the case study specific footprints, with general comments about the need for specific accounting.

We thank the reviewer for this comment, which prompted us to sharpen both the framing of the contribution and the conclusions.

We have rewritten the Conclusion so that it leads with the methodological contribution and its specific merits. Together with the new Introduction paragraph (comment a), the merits of the method are now stated explicitly relative to the alternatives, with references, rather than as general observations.

On the specific suggestion of a numerical comparison against alternative methods, we would note that the established alternatives are not directly commensurable with SPA-DEC at the level of the functional unit. Hotel methods report per guest-night, and the tourism and aquatic-centre LCAs report at facility scale. The one alternative that shares SPA-DEC's per-visit functional unit and uses the same underlying data is

the static annual ratio (total annual emissions divided by annual visitors), and the central methodological argument of the paper is that this single-value approach masks the temporal structure SPA-DEC resolves.

Separately, two specific points:

While it's good to see uncertainty analysis, there is no explanation for the uncertainty values chosen -- this could refer to a standardised method like the pedigree matrix, reference other sources for why these are appropriate uncertainty levels, or give an explanation of how they were estimated.

We thank the reviewer for raising this. The coefficients of variation were assigned by structured expert judgement based on data provenance, following the principle that uncertainty scales inversely with the directness of measurement. Directly logged hourly inputs (electricity, gas, water, and the occupancy denominator) are well constrained and assigned low CVs; secondary emission factors are treated as materially less precise; and annually estimated, default-factor, or spend-based categories are assigned the highest CVs, reflecting their reliance on sparse records and input-output modelling. We have revised the Sensitivity and Uncertainty Analysis section to state this rationale explicitly and have added a supplementary table (Table S1) reporting the per-input CV and its justification, so the basis for each value is transparent and reproducible.

Thank for you for clarifying about PV and geothermal representing embodied emissions amortised over expected system lifetime; I would suggest checking the GHG Protocol definitions for this as I believe the scope 2 guidance includes only emissions at the point of generation, which is in tension with the LCA perspective.

We thank the reviewer for this suggestion. Having reviewed the GHG Protocol definitions as recommended, we confirm that on-site photovoltaic and geothermal generation incurs no point-of-generation emissions, and that the embodied, upstream emissions of this infrastructure are reported separately under Scope 3 using life-cycle emission factors. We have made this explicit in the Results (Scope Breakdown), with a supporting citation to the GHG Protocol. Reporting the operational and embodied components separately keeps the inventory consistent with the GHG Protocol scope definitions while retaining an LCA perspective, the two being complementary rather than alternative treatments of the same emissions. To remove any ambiguity on the operational side, we have additionally clarified that the electricity used to run the geothermal heat-pump system is metered within the facility's purchased electricity and accounted under Scope 2, distinct from the geothermal life cycle factor, so that operational and embodied emissions are not double-counted.

SPA-DEC Responses to Reviewer Comments

Reviewer #1 (Remarks to the Author):

This manuscript introduces SPA-DEC, a dynamic emissions calculator applied to spa and wellness facilities, and represents one of the first sector-specific operational LCA frameworks for this industry. The allocation approach — dividing emissions between visitor-hours and closed-hour maintenance — is methodologically intuitive and a genuine contribution to the field. The case study is well-executed, and the writing is clear throughout. However, several concerns should be addressed before the work can be considered for publication.

We thank the reviewer for their comments and welcome their positive assessment of the allocation methodology and case study. We have addressed each of the concerns raised below in full.

The manuscript contains notable inconsistencies in greenhouse gas scope classification that undermine the methodological rigour of the framework. Fugitive emissions are misclassified: these constitute Scope 1 emissions, not Scope 2. On-site photovoltaic systems, where equipment is purchased rather than leased, are more appropriately categorised under Scope 3 (capital goods) rather than Scope 2. Similarly, company transport is consistent with Scope 3 commuting. These misclassifications recur in the discussion of Figure 2 and should be corrected systematically throughout.

The scope misclassifications have been corrected systematically throughout the revised manuscript, including the discussion of Figure 2 (now Figure 1) and Table 1. Fugitive refrigerant emissions are now correctly classified as Scope 1, reflecting direct releases from owned or controlled equipment. On-site photovoltaic infrastructure, where equipment is purchased rather than leased, has been reclassified as Scope 3 (capital goods), reflecting its embodied upstream manufacturing emissions.

In addition to on-site photovoltaic, we have reclassified the on-site geothermal impact as Scope 3. This is because these emissions relate to the upstream embodied emissions of the energy infrastructure. The Large Wellness Facility uses a binary/closed-loop heat extraction. The geothermal fluid circulates underground, and heat is transferred via a heat exchanger, with no direct venting of geothermal gases. The electricity required for the pump is counted within the electricity consumption (purchased electricity and photovoltaic).

Regarding company transport: we respectfully maintain its classification as Scope 1. Under the GHG Protocol Corporate Standard, fuel combustion from spa-owned

vehicles used in the delivery of visitor services constitutes a direct emission from a controlled source and is therefore Scope 1. Staff commuting is correctly classified as Scope 3 and is reported separately throughout.

The novelty of SPA-DEC lies primarily in its allocation criteria and dynamic temporal resolution; however, this methodological contribution is underexplored relative to the case study results. The manuscript would benefit from a more explicit discussion of how the dynamic electricity grid mix varies seasonally across the study geography, as this is central to the framework's claimed advantage over static approaches.

The methodological contribution of SPA-DEC has been substantially strengthened in the revised manuscript. The Introduction now includes a dedicated paragraph explicitly positioning SPA-DEC's allocation approach relative to two alternative methodologies: the static annual ratio and dynamic hourly allocation without open/closed hour distinction. Then we demonstrate why neither adequately resolves the challenges of visitor-level attribution in spa facilities.

Regarding seasonal grid mix variation, this is now addressed explicitly in the Results. Using hourly grid generation data sourced from the national transmission system operator, the purchased electricity mix was resolved at hourly resolution for each month of 2024. By linking this hourly grid composition data to hourly facility electricity consumption, SPA-DEC applies a temporally resolved emission factor.

Furthermore, the analysis is limited to a single year; multi-year profiles (four or more years) would substantially strengthen the conclusions regarding seasonal patterns and decarbonisation trajectories.

The analysis is validated across two contrasting facilities for the full 2024 calendar year. The inclusion of a second, structurally distinct facility strengthens conclusions regarding seasonal patterns and demonstrates that SPA-DEC's capability is not confined to a single operational context. We acknowledge that multi-year profiles would further strengthen decarbonisation trajectory conclusions, and this is identified as a priority for future work in the revised Limitations section.

No sensitivity analysis is presented. Given the reliance on normalised non-metered inputs (waste, staff travel), a formal uncertainty or sensitivity assessment is essential to substantiate the robustness of the reported results.

A full Monte Carlo uncertainty and sensitivity analysis has been added to the Results. Uncertain inputs are represented as lognormal multipliers with coefficients of variation calibrated by data quality tier, with higher uncertainty assigned to non-metered categories including waste, staff commuting, and fugitive losses. The

analysis yields 95% uncertainty intervals and Spearman rank-correlation tornado diagrams for both facilities, identifying the dominant uncertainty drivers at each site and confirming the robustness of the reported per-visitor footprints.

The full life cycle inventory is not made available, limiting reproducibility. Authors should provide the inventory data as supplementary material in accordance with open science principles.

The full results and figure data underlying the case studies will be provided upon acceptance, enabling direct verification of the reported outputs. The supplementary includes the full methodological inventory: emission factor sources for consumables, treatments, and cleaning chemicals (Table S4); waste treatment emission factors (Table S5); the structure of metered and non-metered input categories (Tables S1–S3); and full case study setup descriptions for both facilities. The underlying facility operational activity data are commercially sensitive and cannot be disclosed in full; however, a more comprehensive inventory containing facility-level emission factors and category-level totals will be made available in full upon acceptance to support open science and reproducibility. The complete SPA-DEC codebase and spreadsheet template will be made publicly available via GitHub upon acceptance.

Total emissions figures (39,000,000–49,000,000 kg CO□e) should be expressed in kt CO□e for readability and consistency with reporting conventions under the GHG Protocol and ISO 14064.

The total emissions figures in the Introduction have been converted from kg to kt CO_2e for readability and consistency with GHG Protocol and ISO 14064 reporting conventions.

Figure 6 presents per-visit footprint data for the eighth day of each month only. The rationale for this choice is not explained; monthly averages would be more representative and should be justified or replaced accordingly.

Figure 6 (now Figure 5) has been revised to present monthly averages across all days of each calendar month for both facilities, providing a more representative picture of seasonal variation in per-visit footprints.

Similarly, the choice of a 4-hour functional unit warrants explicit justification in the Methods section.

We note that the 4-hour and 1.5-hour durations are not the functional unit of the study, which is defined as one visit from entry to exit regardless of duration. These

durations are illustrative standard values reflecting the typical commercial entry period at each facility, used to enable consistent temporal and cross-facility comparison in the figures. A clarifying statement to this effect has been added to the Results. SPA-DEC is not constrained to any fixed duration. The dynamic allocation framework produces a per-visitor footprint for any stay length.

Results would benefit from greater quantitative comparison across emission categories, enabling the reader to assess relative contributions more precisely. The current presentation is predominantly descriptive.

The revised manuscript presents category-level contributions across multiple figures. Figure 1 provides scope-level percentage breakdowns for both facilities. Figure 5 decomposes the per-visit footprint by individual emission source across all months, enabling direct quantitative comparison of category contributions and their seasonal variation. Figure 7 provides Spearman rank-correlation coefficients quantifying the relative influence of each input on output uncertainty. Underlying data will be made publicly available, enabling readers to interrogate category-level contributions in full.

The validation of SPA-DEC on a single facility — albeit a large and well-documented one — limits the generalisability of the framework. The inclusion of additional case studies, whether across distinct spa typologies or contrasting geographies with different grid mixes, climatic profiles, and operational scales, would substantially strengthen the claim that SPA-DEC is scalable and transferable. In its current form, the framework's broader applicability remains an assertion rather than a demonstrated outcome.

The revised manuscript includes a second case study: a university-based swimming pool and wellness zone. This provides contrasting validation context across facility scale, operational complexity, energy mix, and geography. The two facilities differ substantially. The Large Wellness Facility is a large thermal resort with geothermal and photovoltaic integration, a diversified three-scope emission profile, and over 1.6 million annual visitors; the Small Wellness Facility is a compact, two-source facility with a visitor base an order of magnitude smaller. SPA-DEC's core allocation logic is applied without modification across both, demonstrating transferability across contrasting spa typologies and data maturities. The claim of scalability is therefore a demonstrated outcome of the revised manuscript rather than an assertion.

The repeated and prominent framing of Therme Bucharest throughout the manuscript risks lending a promotional tone to what should remain a methodologically focused scientific contribution. The case study should be

introduced once and thereafter treated as an illustrative vehicle for the framework, rather than a subject of sustained attention.

The primary case study facility has been anonymised throughout the revised manuscript and is referred to consistently as "Large Wellness Facility." The facility is introduced once and thereafter treated as an illustrative vehicle for the framework, with the methodological contribution remaining the primary focus throughout.

The work addresses a genuine gap in sectoral LCA coverage and the core allocation methodology is novel within its context. However, the scope misclassifications, absence of sensitivity analysis, limited temporal scope, and underdeveloped methodological narrative currently constrain the manuscript's contribution. Revision addressing these points would substantially improve its suitability for publication.

We thank Reviewer 1 for the constructive and thorough assessment. Each of the concerns (scope misclassifications, absence of sensitivity analysis, limited temporal scope, and underdeveloped methodological narrative) have been addressed in the revised manuscript, as detailed above. We are confident that the revisions substantially strengthen the contribution and its suitability for publication.

<u>Reviewer #2 (Remarks to the Author):</u>

The authors provide a timely and essential contribution to an under-researched sector. The SPA-DEC framework is commendable for its scalability and its ability to translate complex Scope 1, 2, and 3 emissions into a "per-visitor" metric. By normalizing non-metered sources like staff travel and waste alongside real-time energy data, the tool offers a transparent benchmarking system that is highly supportive of industry-wide decarbonization.

We thank the reviewer for their comments and welcome their positive assessment of the scalable and transparent methodology. We have addressed each of the concerns raised below in full.

While the validation at Therme Bucharest provides a strong proof-of-concept, the transition from a footprinting tool to rigorous science requires a deeper dive into the data already captured. How do emissions profiles shift during peak social hours versus maintenance cycles, etc.? Providing this kind of granular data analysis (hourly, daily, and annual patterns) would transform the paper from a case study of one facility into a data set for the wellness industry.

The revised manuscript provides granular temporal analysis across multiple resolutions. Figure 2 presents hourly per-visitor metered consumption emissions across the full year, Figure 3 disaggregates closed-hour maintenance emissions by

source, Figure 5 provides monthly per-visit footprint decompositions by emission category, and Figure 6 presents average daily and monthly patterns by weekday and season. Together these figures distinguish peak operational hours from maintenance cycles and reveal how emissions profiles shift across daily, weekly, and seasonal timescales. The addition of a second case study further extends the dataset beyond a single facility, providing a comparative reference point for the wellness industry.

Because this study is validated on a single, unique facility (Therme Bucharest), its scientific credibility relies on the depth of the data rather than its breadth. By analyzing these high-resolution temporal patterns, the authors could reveal how the Climate Change KPIs on Scope 1, 2 and 3 fluctuates in relation to other critical reporting points like peak water demand or seasonal waste spikes.

The revised manuscript now validates SPA-DEC across two contrasting facilities, addressing the concern about single-facility credibility.

This would allow for a more transparent sustainability report that recognizes the energy-water-waste nexus rather than treating carbon as a standalone variable.

We thank the reviewer for this contribution. We agree this would be a valuable extension of the framework. SPA-DEC's primary scope is GHG accounting aligned with organisational reporting requirements under the GHG Protocol and ISO 14064. Extending to a full nexus analysis incorporating water demand and waste intensity alongside carbon would require additional inventory development and impact characterisation beyond the current framework. Multi-impact assessment is now acknowledged explicitly in the revised Limitations section as a priority for future work.

The scenario modeling (geothermal and PV) provides appropriate best practices for the industry. However, to elevate this framework from a reporting tool to a strategic transition roadmap, the framework should help facility managers navigate the proactive transition.

The positive assessment of the scenario modelling is appreciated. The revised Implementation section now explicitly frames SPA-DEC as a strategic planning tool alongside its reporting function.

<u>Reviewer #3 (Remarks to the Author):</u>

The authors address the lack of sector-wide carbon accounting methods for the spa and wellness industry, which is known to have high energy consumption but limited sustainability measurement frameworks. To fill this gap, they develop and

demonstrate a sector-specific emissions calculator. Further, they present detailed the results for Therme Bucharest, one of the worlds largest spa and wellness centers, which provides insights on the main sources for GHG emissions for spa and wellness centers. While I believe this study presents useful information, I suggest major revisions prior to publication to improve the relevance of the study.

We thank the reviewer for their comments and welcome their positive assessment of the case study. We have addressed each of the concerns raised below in full.

1. Consider broadening the scope and usability of the tool: the authors discuss how the tool can be used across spa centers, but I think it would be interesting if there was some applicability to any other service-based infrastructure such as other leisure facilities, theme parks, etc. Therefore, the authors should clarify the broader scientific contribution of the work, and make modifications to the tool, if possible, to enable wider usage.

The broader applicability of SPA-DEC's allocation framework is now explicitly addressed in the revised manuscript. The Strengths section discusses the transferability of the core allocation logic to a wider class of visitor-economy service facilities, including leisure centres, aquatic facilities, and other service-based infrastructure where operational emissions must be attributed to individual users. This is demonstrated in practice through the inclusion of a second case study, which applies SPA-DEC without modification to the core framework across a fundamentally different operational context. Full adaptation to other sectors would require sector-specific inventory development, but the methodological framework does not present barriers to extension and is designed with this modularity in mind.

2. Need for uncertainty analysis: Given that a portion of impacts (14%) come from the consumption of products, and that this data appears to come from a variety of sources, an uncertainty analysis of these impacts is necessary. As a result, the authors could incorporate discussion of uncertainty in input data, error propagation through the allocation model, and confidence intervals for per-visitor emissions.

A full Monte Carlo uncertainty and sensitivity analysis has been added to the Results (Figure 7). The analysis propagates uncertainty through the allocation model across all input categories, with coefficients of variation calibrated by data quality tier, including higher uncertainty assigned to product consumption and other non-metered categories. The results include 95% confidence intervals for per-visitor emissions and Spearman rank-correlation sensitivity rankings for both facilities, directly identifying the contribution of each input category to total output variance.

3. Additional environmental impact discussions: The authors only report GHG emissions, with the reasoning that this is the most common metric reported in LCA. While this is true, I believe presenting results for other impact categories such as water use, particulate matter emissions, eutrophication impacts etc., would be very valuable. For example, I would expect spas to be water-intensive due to the large heated pools and thermal baths, continuous filtration and water replacement, intensive laundry operations, and extensive cleaning and chemical treatment. Based on the description of the methods, reporting these additional impacts should be straightforward, assuming that all of the inventories were built out in an LCA database such as SimaPro.

We thank the reviewer for this contribution. We agree that multi-impact assessment would be valuable for water-intensive facilities. However, SPA-DEC is designed as an accessible, sector-specific emissions calculator intended for use by facility operators or visitors without specialist LCA software. Conducting the case study analysis within SimaPro or equivalent platforms would undermine this design principle and defeat the purpose of developing a standalone tool. Emission factors for relevant inventory items were derived from Ecoinvent datasets characterised using the IPCC GWP100 method and implemented within SPA-DEC. Extending the framework to additional impact categories such as water use and eutrophication is a clear priority for future development and is now acknowledged in the revised Limitations section.

Reviewer #4 (Remarks to the Author):

This paper presents a carbon-footprinting approach intended for use by spa facilities, together with a case study of the Thermae Bucharest spa. The approach uses partial dynamic allocation of carbon emissions to specific visits (based on energy and water use metered to be consumed within hourly periods matching the visit) together with annual allocation of overheads and closed-hours emissions to all visitors.

We thank the reviewer for their comments. We have addressed each of the concerns raised below in full.

I am unsure where the authors intend the main significance of the paper to lie. The approach seems to be an interesting application of attributional carbon accounting to a particular type of organisation, which is nontrivial due to the different types of energy and purchases involved, but it's not clear in the way it's presented what is novel about this. If the intended contribution is about establishing the methodology that spas should use, there should be greater clarity on the need for this (why are spas different from other patterns that could be copied?), what they key choices are, and why the proposed method is better than alternative approaches that could have been proposed (see e.g. the questions listed below). If the intended contribution is

about the findings from the case study, there should be greater clarity on what the implications of this are and how they can be used to make decisions that were not possible before.

The revised manuscript addresses this directly on two fronts. First, the Introduction now includes a dedicated paragraph explicitly positioning SPA-DEC's methodological contribution relative to two alternative approaches: the static annual ratio and dynamic hourly allocation without open/closed hour distinction. We explain the limitations of each and demonstrate why SPA-DEC's approach is methodologically superior. This clarifies both why spas require a sector-specific tool and why the proposed allocation logic is preferable to simpler alternatives. Second, the Goal and Scope section now states four explicit intended applications of the analysis in accordance with ISO 14040, establishing that the primary contribution is methodological: the framework itself, rather than the findings of any individual case study. The case study results are presented as a demonstration of what the framework makes possible: temporal hotspot identification, renewable scenario evaluation, and visitor-level attribution – none of which are achievable with conventional annual inventory approaches.

The details of the methods can be made more consistent and explicit. Some of the case study results would benefit from more explanation. As per LCA in general, uncertainty and sensitivity to assumptions should be discussed.

The revised manuscript addresses each of these points in full. Methodological consistency and explicitness have been improved throughout the Methods section, case study results are accompanied by expanded quantitative and interpretive discussion, and a full Monte Carlo uncertainty and sensitivity analysis has been added to the Results. Each specific concern is addressed in detail below.

While (with some corrections/clarifications, as below) the paper clearly represents a solid piece of publishable work, I am not sure that it meets the criteria for publication in Communications Earth & Environment, namely that the conclusions are novel (as above, this should be made more explicit what they are and why they are novel), and that "the underlying data are generated and processed at a state-of-the-art level, including tracking of uncertainties" (some level of discussion of uncertainties and sensitivity is essential in this type of analysis, but currently only mentioned in passing).

Both criteria are addressed in the revised manuscript. The novelty of SPA-DEC's methodological contribution is now explicitly established in the Introduction through a direct comparison with alternative approaches, and the Goal and Scope section frames the four intended applications of the analysis in accordance with ISO 14040. A full Monte Carlo uncertainty and sensitivity analysis has been added to the

Results, with uncertainty propagated through the allocation model and reported at state-of-the-art level across both case study facilities.

Line 40: the lack of estimates of *global* GHG footprints of the spa industry doesn't seem relevant to this study

The reference to the absence of global GHG footprint estimates for the spa industry has been removed from the Introduction. The revised text instead focuses directly on the lack of a standardised per-visit emission metric and the absence of consistent boundaries and allocation methods, which is directly relevant to the contribution of this study.

Line 111: this cited study seems to be about cities, and doesn't obviously demonstrate that scope 3 emissions often represent the largest portion of an organisation's footprint?

Reference 34 has been replaced with a more directly applicable source. The revised manuscript cites Ducoulombier (2021), which addresses the importance and scale of Scope 3 emissions specifically in the context of organisational reporting, directly supporting the claim made at this point in the Introduction.

Line 132: should explain how the tool is "aligned with emerging carbon offsetting frameworks"

The revised manuscript now explains the basis of alignment: the mechanism requires verified, visit-level carbon attribution, which is precisely what SPA-DEC's dynamic per-visitor allocation produces.

Figure 1: What does "on-site geothermal" represent that leads to it being the 6th largest category of annual emissions? This seems surprising and worth explaining.

The original Figure 1 has been removed in the revised manuscript; the explanation of the geothermal contribution is now incorporated into the Scope Breakdown section. On-site geothermal appears as the sixth largest emission category despite carrying one of the lowest life cycle emission factors of any energy source. This figure represented the embodied emissions of the geothermal infrastructure amortised over the system lifetime, rather than direct release of geothermal gases. Its material contribution to the annual total reflects the scale of geothermal heat consumption: as the facility's primary thermal energy source, the volume of heat extracted is very large, and even at low emission intensity, the absolute annual total is significant. An explanatory note to this effect has been added to the Results Section 2.2.

Table 1: Why is geothermal four-hourly rather than hourly like the others? How is this accounted for in the methods, which only mention hourly energy consumption?

Table 1 was originally specific to the Large Wellness Facility but has been updated to reflect typical SPA-DEC inputs. This includes hourly resolution for geothermal, consistent with the other metered sources. In the Large Wellness Facility case study, geothermal consumption was recorded at four-hourly intervals. SPA-DEC handles sub-hourly data gaps through uniform temporal disaggregation: four-hourly totals are divided equally across the constituent hours, preserving the overall energy balance while aligning with the hourly allocation framework. This is consistent with SPA-DEC's broader approach to data maturity. The framework is designed to accommodate varying input resolutions without compromising the integrity of the per-visitor allocation, and higher-resolution metering can be substituted directly where available. A clarifying note on this approach has been added to the Temporal Allocation section of the Methods, which also explains where disaggregation is and is not appropriate.

Figure 3: What do the "geothermal" and "PV" emissions represent here? The methods do not explain how they are calculated.

The revised manuscript now explains what geothermal and PV emissions represent in the Results Section 2.2. Both are lifecycle emission factors sourced from Ecoinvent, representing the embodied upstream emissions of the on-site infrastructure, amortised over the system lifetime and expressed per kWh of energy produced or consumed. Neither represents direct combustion emissions.

Line 189: Presumably the time period over which this is done is an important modelling choice, but it's not discussed?

The choice of a one-year aggregation period for non-metered inputs is now explicitly justified in the revised Methods. Annual aggregation aligns with standard GHG reporting cycles under the GHG Protocol and ISO 14064, ensures the normalisation denominator captures the full seasonal range of visitor throughput, and smooths short-term procurement variation that would otherwise introduce noise into per-visitor estimates.

Line 197: The justification for the open/closed hours method is also not clear -- how do you know that you shouldn't assume the closed-hours energy consumption represents a baseline with the marginal per-visitor footprint being the excess above this, for example, rather than 100% of the open-hours energy consumption?

An allocation rationale paragraph has been added to the Methods following the temporal allocation steps. Regarding open-hour allocation: 100% of open-hour energy is attributed to visitors because all energy consumed during opening hours is directly in service of delivering the visitor experience. Regarding closed-hour allocation: SPA-DEC adopts a full-cost approach consistent with the ISO 14040/44 principle that all inputs within the defined system boundary are attributed to the functional unit. The marginal approach of leaving closed-hour baseline energy unattributed would systematically understate the total service footprint and undermine cross-facility comparability. Closed-hour overheads are additionally allocated equally per visitor rather than weighted by duration, as these costs are fixed and independent of stay length.

Line 215: Why is a 4-hour visit used? Are the results sensitive to this?

We note that the 4-hour and 1.5-hour durations are not the functional unit of the study, which is defined as one visit from entry to exit regardless of duration. These durations are illustrative standard values reflecting the typical commercial entry period at each facility, used to enable consistent temporal and cross-facility comparison in the figures. A clarifying statement to this effect has been added to the Results. SPA-DEC is not constrained to any fixed duration. The dynamic allocation framework produces a per-visitor footprint for any stay length. Regarding sensitivity: the per-visit footprint is partially sensitive to duration, as the open-hour metered component scales with stay length, while closed-hour overhead and non-metered components are fixed per visitor regardless of duration.

Line 229: If individual days are so variable in visitor numbers, why is it relevant to present results from arbitrary individual days? The suggestion is that we should infer seasonal patterns from Figure 6, but this is already shown more clearly in the later figures? Why are October and December so high?

Figure 6 (now Figure 5) in the revised manuscript now presents monthly averages across all days of each calendar month for both facilities, replacing the previous single-day snapshots. The elevated footprints in October and December are now explicitly explained in the Results: October reflects declining PV contribution combined with lower visitor numbers, which increases the overhead emissions allocated to each visit; December records the highest per-visit footprint due to peak natural gas demand when geothermal backup requirements are greatest and PV generation is negligible.

Line 260: Isn't the 32% figure based simply on the total annual energy consumption and emissions of the different energy sources? Can you elaborate how the dynamic allocation enables this analysis?

While the reduction could be derived from annual energy totals alone, the dynamic framework resolves this avoided-emissions benefit at daily-aggregated resolution across the year, as shown in Figure 4, revealing that the renewable contribution is most material during summer months when PV generation peaks for the Small Wellness Facility, and during the winter months when the thermal consumption requirement and therefore geothermal contribution is higher for the Large Wellness Facility.

Line 278: Currently, the results do not prove that the approach can "accurately" do this

The revised manuscript no longer contains this claim. The section in which it appeared has been substantially rewritten, and the language now describes what the results demonstrably show: that SPA-DEC's allocation logic is transferable across facility types and yields interpretable outputs at multiple temporal resolutions.

Line 280: "very straightforward" -- is it? If less real-time occupancy or metered data was available, wouldn't that make it difficult to apply your dynamic allocation method?

The revised manuscript no longer contains this claim. The section in which it appeared has been substantially rewritten, and the language now reflects the data requirements of the dynamic allocation method without overstating its ease of application.

Line 296: Rationale for why closed-hour overheads should be allocated per-visitor (not accounting for their duration of visit) isn't clear

This is addressed in the allocation rationale paragraph added to the Methods following the temporal allocation steps. Closed-hour overheads are fixed costs incurred independently of how long any individual visitor stays. Equal per-visitor allocation is therefore the methodologically appropriate choice: weighting by visit duration would imply that longer-staying visitors bear responsibility for a greater share of costs that do not, in practice, vary with their presence or duration.

Line 353: These operational examples all seem to be good examples where having hourly energy consumption/emissions data is indeed useful -- but it's not obvious that

the dynamic allocation per-visitor helps? e.g. reducing peak grid reliance will help reduce emissions regardless of how many visitors are present at the time. The significance of the allocated footprints for different decisions is not fully clear...

We thank the reviewer for this contribution. The significance of the per-visitor dimension for different decisions is addressed in the Dynamic Emissions Accounting section of the Interpretation. The reviewer is correct that hourly energy data supports operational decisions such as peak grid management independently of visitor allocation. The per-visitor dimension adds three things that aggregate facility data cannot provide: a functional unit that is directly comparable across facilities, time periods, and ticket types; visitor-level attribution required for carbon communication and offsetting mechanisms; and the demonstration that the footprint of an individual visit varies materially depending on when it occurs, which is actionable for demand management, visitor incentives, and transparency. These are decisions that require knowing not just total facility emissions but how they are distributed across visits.

Line 391: ... this scenario is switching from retrospective accounting of per-visit footprints, to trying to support a decision (presumably) about when or whether a visitor will come to the spa. It's not obvious that the metric being reported is appropriate for both; if you are recommending its use for this decision can you elaborate on this?

The pre-visit and post-visit uses of SPA-DEC apply the same functional unit and allocation logic — the only difference is that pre-visit estimates use forecast inputs rather than realised metered data. This consistency makes the metric appropriate for both purposes: a visitor comparing footprints for different visit windows is working from the same methodological basis as the post-visit receipt they would receive.

Line 439: the goal and scope should also discuss the goal with respect to the decisions that this analysis aims to support (e.g. in relation to the question about different decisions above)

The Goal and Scope section has been revised to explicitly state four intended applications of the analysis, covering facility-level benchmarking and CSR reporting, operational decarbonisation decision-making, visitor-level carbon communication and offsetting, and the development of a scalable sector-wide framework. These directly correspond to the decisions the analysis aims to support.

Line 449: "gate-to-gate" -- isn't this confusing as it's not the same sense as in "cradle-to-gate"?

The term "gate-to-gate" has been removed from the revised manuscript. The system boundary is described throughout as "from visitor entry to exit," avoiding any confusion with the cradle-to-gate convention used in product LCA.

Line 450: Elsewhere is it not linked to visit duration?

The functional unit is defined as one visit from entry to exit, independent of duration. A clarifying statement has been added to the Results noting that the 4-hour and 1.5-hour durations used in the figures are illustrative values reflecting typical visit lengths at each facility, used solely to enable consistent temporal and cross-facility comparison. SPA-DEC is not constrained to any fixed duration. The dynamic allocation framework produces a per-visitor footprint for any stay length.

Line 499 and following: The equations are not consistent - the later ones seem to overlap with the first ones but using different notation. Open/closed hours are not reflected in Equation 2.

Equation 2 has been revised to explicitly reflect the open/closed hour distinction, and the notation has been harmonised across the equation set and Steps 1–4 to ensure consistency throughout the Methods.

Line 514: This should be elaborated if included.

The sentence has been elaborated in the revised manuscript.

Figure 8: How are "per unique visitor" outputs obtained separately from "normalisation and dynamic emissions allocation"? What does "normalisation" mean here -- it doesn't seem to be referring to LCIA normalisation?

The flow diagram has been revised. "Normalisation and dynamic emissions allocation" has been replaced with "Dynamic visitor attribution and allocation" to avoid confusion with LCIA normalisation. The per-visitor output is now clearly positioned within the Outputs box as the direct result of the allocation step, clarifying that it is not obtained separately from the attribution process but is its primary output.

Line 527: This is inconsistent with equation 2

Equation 2 has been revised to explicitly reflect the open/closed hour distinction, and the notation has been harmonised across the equation set and Steps 1–4 to ensure consistency throughout the Methods.

Line 538: Wrong H

The incorrect H notation has been corrected.

Line 540: Unclear what "the visitors associated with day d" means - it sounds like it should be the calendar day but then you say "added to the next N_d visits". Does it make any difference?

Step 3 (now Step 4) has been written explicitly in the revised manuscript. Closed-hour emissions are aggregated and allocated equally across all unique visitors of the same calendar day. This provides a transparent and physically meaningful allocation rule that is consistent with the full-cost attribution principle applied throughout the framework.

Line 542: How are the outlier thresholds set? Why is it right to reallocate 100% of outliers?

Step 4 (now Step 3) has been clarified in the revised manuscript. Outlier hours are identified using a statistically grounded threshold: open-hour consumption values exceeding three standard deviations above the facility mean. This is consistent with standard anomaly detection practice in environmental monitoring. Excess emissions are capped and transferred to the closed-hour overhead pool rather than fully reclassified, preserving the total emissions balance.

Line 584: "This approach ensures simplicity while methodological rigour," - unclear what this is referring to

The sentence has been rewritten for clarity. The revised text explicitly identifies the approach of treating the per-visitor waste footprint as an annually normalised constant and explains that this balances methodological rigour with practical data requirements, accommodating facilities where waste data are available only at annual resolution.

Line 611-645: This all seems like generic Scope 3 GHG accounting; is there intended to be anything specific to spas here?

This reviewer is correct to point this out. The Business Operations section applies standard GHG Protocol Scope 3 calculation methods, which are necessarily generic as they follow internationally recognised guidance. The spa-specific content in this section lies in the relevance criteria applied to determine inclusion and exclusion.

Only emission sources that exist because of the facility's operation and visitor hosting are included, and Table S6 explicitly documents categories excluded as irrelevant to spa operation. The earlier consumables, treatments, and cleaning sections contain the spa-specific inventory content. The Business Operations section is intentionally positioned as a modular, standardised component that spa operators can implement without requiring bespoke methodological development for each category.

Responses to Reviewer Comments 2

COMMSSUSTAIN-26-0048A-Z

Reviewer #1 (Remarks to the Author):

The authors have addressed all major concerns raised in the first review in a thorough and convincing manner. The revised manuscript is substantially stronger.

The Monte Carlo uncertainty analysis is a welcome addition, the scope classifications have been corrected throughout, and the anonymisation of the primary facility resolves the promotional tone flagged previously. The shift to monthly averages and the more explicit methodological framing also improve the overall clarity of the work.

The second case study demonstrates transferability effectively, though it is worth briefly acknowledging that a university wellness facility operates under a fundamentally different business model to a commercial spa, which may carry implications.

On reproducibility, the commitment to release the full inventory and codebase via GitHub upon acceptance is appreciated. Making anonymized category-level totals available at the review stage would have been preferable, but this does not affect the overall assessment.

I recommend acceptance.

We thank the reviewer for their constructive comments from the current and previous review rounds and for recommending acceptance.

We agree with the reviewer's point that it's worth acknowledging the difference in business model between the case studies. For this reason, Section 2.2.1 has been renamed "Case Studies as Contrasting Applications". We have added a paragraph into this section explaining that the facilities are chosen to demonstrate the applicability of the methodology and that the resulting footprints reflect both operational efficiency and the breadth of amenities provided and should not be interpreted as a direct ranking of relative sustainability. The implication of this is that cross-facility benchmarking with SPA-DEC requires aligned system boundaries and comparable service definitions. This is also explained in Section 4.1 Goal and Scope.

The data underlying all figures and SPA-DEC output inventory are openly available on Figshare at https://doi.org/10.6084/m9.figshare.32825969, while the SPA-DEC repository, which includes the model library, the scripts that regenerate the figures from the aggregated outputs, a synthetic example dataset that allows the complete pipeline to be run without access to the proprietary facility data, a results

visualisation dashboard, and the per-facility configuration choices used for the two case studies is available on Zenodo at https://doi.org/10.5281/zenodo.21038901.

Reviewer #3 (Remarks to the Author):

Thank you for the revised manuscript and for the detailed point-by-point responses. I appreciate the authors’ efforts in addressing the reviewer comments. In general, I feel that my previous concerns have been satisfactorily addressed, and the revisions have improved the clarity and overall quality of the manuscript. Based on the changes made, I have no further major comments and would support the manuscript for publication in its current form.

We thank the reviewer for their constructive comments across earlier review rounds and for supporting publication.

Reviewer #4 (Remarks to the Author):

Thank you to the authors for making revisions which substantially address the reviewers' comments about clarity of the methodology and application of the presented tool, including the uncertainty analysis which is an important addition to the proposed approach.

We thank the reviewer for recognising that the revisions have substantially addressed the previous concerns on methodological clarity and application. We address the two outstanding aspects of methodological positioning and the two specific points below.

The authors clarified that their focus is the paper's methodological contribution, rather than the case studies. With this, I would expect that the method be situated explicitly against the literature/state-of-the-art approaches, and to have specific conclusions about the merits of the proposed method, which I still find is not the case. By this I mean, for example:

(a) alternative methods -- these are added in a new paragraph in general terms but with no references or specifics. There are relevant approaches/examples, perhaps outside of the very narrow spa application, of per-visitor carbon accounting methods e.g. for hotels -- if this is the methodological problem being solved then it deserves explicitness and references. Alternatively if, say, it's the "LCA-aligned emissions-factor selection" suitable for spas which was previously missing, what data is currently available to spa analysts and how should they know that your figures are better?

We thank the reviewer for this comment. We have added a dedicated paragraph to the Introduction that situates SPA-DEC explicitly against the relevant state of the art.

The revised text identifies the established per-visitor and per-guest-night methods in adjacent visitor-economy sectors and cites them directly: the EU Product Environmental Footprint Category Rule for hotel accommodation services and hotel-specific carbon tools such as the Hotel Carbon Measurement Initiative, which apportion whole-property emissions to a guest-night using floor-area and occupancy-based allocation but rely on annual input data rather than temporally resolved emission factors. We distinguish these from the rigorous but facility- or scenario-level life-cycle studies in tourism and aquatic-centre contexts, which are too data-intensive for routine operational use. For spas specifically, we now state plainly that the only published footprints divide annual direct-energy consumption by user numbers, without time resolution, closed-hour treatment, or documented emission-factor provenance.

This directly addresses the reviewer's question of what is currently available to spa analysts and why SPA-DEC's figures are an improvement. The data presently available to the sector is limited to undocumented, energy-only annual averages; SPA-DEC's contribution is to provide a transparent, LCA-aligned, and temporally resolved alternative whose emission factors and allocation rules are documented and reproducible. The paragraph closes by stating the specific methodological position SPA-DEC occupies: the combination of visit-level temporal allocation, an explicit open/closed-hour distinction, and standardised, traceable LCA-aligned emission factors applied at hourly rather than annual resolution, with on-site renewable infrastructure represented through life-cycle factors rather than assumed to be zero.

(b) findings -- if proposing a new method for calculating per-visitor footprints, one might expect comparisons to alternative methods that could have been used -- either to show an improvement over simpler methods, or to show validation against a more sophisticated method. Currently the conclusions focus on the case study specific footprints, with general comments about the need for specific accounting.

We thank the reviewer for this comment, which prompted us to sharpen both the framing of the contribution and the conclusions.

We have rewritten the Conclusion so that it leads with the methodological contribution and its specific merits. Together with the new Introduction paragraph (comment a), the merits of the method are now stated explicitly relative to the alternatives, with references, rather than as general observations.

On the specific suggestion of a numerical comparison against alternative methods, we would note that the established alternatives are not directly commensurable with SPA-DEC at the level of the functional unit. Hotel methods report per guest-night, and the tourism and aquatic-centre LCAs report at facility scale. The one alternative that shares SPA-DEC's per-visit functional unit and uses the same underlying data is

the static annual ratio (total annual emissions divided by annual visitors), and the central methodological argument of the paper is that this single-value approach masks the temporal structure SPA-DEC resolves.

Separately, two specific points:

While it's good to see uncertainty analysis, there is no explanation for the uncertainty values chosen -- this could refer to a standardised method like the pedigree matrix, reference other sources for why these are appropriate uncertainty levels, or give an explanation of how they were estimated.

We thank the reviewer for raising this. The coefficients of variation were assigned by structured expert judgement based on data provenance, following the principle that uncertainty scales inversely with the directness of measurement. Directly logged hourly inputs (electricity, gas, water, and the occupancy denominator) are well constrained and assigned low CVs; secondary emission factors are treated as materially less precise; and annually estimated, default-factor, or spend-based categories are assigned the highest CVs, reflecting their reliance on sparse records and input-output modelling. We have revised the Sensitivity and Uncertainty Analysis section to state this rationale explicitly and have added a supplementary table (Table S1) reporting the per-input CV and its justification, so the basis for each value is transparent and reproducible.

Thank for you for clarifying about PV and geothermal representing embodied emissions amortised over expected system lifetime; I would suggest checking the GHG Protocol definitions for this as I believe the scope 2 guidance includes only emissions at the point of generation, which is in tension with the LCA perspective.

We thank the reviewer for this suggestion. Having reviewed the GHG Protocol definitions as recommended, we confirm that on-site photovoltaic and geothermal generation incurs no point-of-generation emissions, and that the embodied, upstream emissions of this infrastructure are reported separately under Scope 3 using life-cycle emission factors. We have made this explicit in the Results (Scope Breakdown), with a supporting citation to the GHG Protocol. Reporting the operational and embodied components separately keeps the inventory consistent with the GHG Protocol scope definitions while retaining an LCA perspective, the two being complementary rather than alternative treatments of the same emissions. To remove any ambiguity on the operational side, we have additionally clarified that the electricity used to run the geothermal heat-pump system is metered within the facility's purchased electricity and accounted under Scope 2, distinct from the geothermal life cycle factor, so that operational and embodied emissions are not double-counted.
